# The cell cycle protein MAD2 facilitates endocytosis of the serotonin transporter in the neuronal soma

**DOI:** 10.15252/embr.202153408

**Published:** 2023-08-02

**Authors:** Florian Koban, Michael Freissmuth

**Affiliations:** ^1^ Institute of Pharmacology and the Gaston H. Glock Research Laboratories for Exploratory Drug Development, Center of Physiology and Pharmacology Medical University of Vienna Vienna Austria

**Keywords:** endocytosis, mitotic checkpoint, raphe neurons, recycling endosome, serotonin transporter, Cell Cycle, Membranes & Trafficking, Neuroscience

## Abstract

Monoamine transporters retrieve serotonin (SERT), dopamine (DAT), and norepinephrine (NET) from the synaptic cleft. Transporter internalization contributes to the regulation of their surface expression. Clathrin‐mediated endocytosis of plasma membrane proteins requires adaptor protein‐2 (AP2), which recruits cargo to the nascent clathrin cage. However, the intracellular portions of monoamine transporters are devoid of a conventional AP2‐binding site. Here, we identify a MAD2 (mitotic arrest deficient‐2) interaction motif in the C‐terminus of SERT, which binds the closed conformation of MAD2 and allows for the recruitment of two additional mitotic spindle assembly checkpoint (SAC) proteins, BubR1 and p31^comet^, and of AP2. We visualize MAD2, BubR1, and p31^comet^ in dorsal raphe neurons, and depletion of MAD2 in primary serotonergic rat neurons decreases SERT endocytosis in the soma. Our findings do not only provide mechanistic insights into transporter internalization but also allow for rationalizing why SAC proteins are present in post‐mitotic neurons.

## Graphical abstract

Proteins of the spindle assembly checkpoint mediate the endocytosis of the serotonin transporter (SERT) via a direct interaction between MAD2 and SERT.

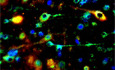

## Introduction

After their release into the synaptic cleft, the monoamine neurotransmitters serotonin, dopamine, and norepinephrine are retrieved by their cognate transporters (serotonin transporter, SERT; dopamine transporter, DAT; norepinephrine transporter, NET), which reside in the presynaptic compartment in the vicinity of the active zone (Qian *et al*, 1995; Hersch *et al*, 1997; Tao‐Cheng & Zhou, 1999; Block *et al*, 2015). Thus, the transporters shape neurotransmission and neuromodulation by limiting diffusion, clearing the synapse, and replenishing the vesicular pool of monoamine neurotransmitters. Their action translates in the regulation of, for example, mood, reward, movement, appetite, or addictive behavior (Kristensen *et al*, 2011). SERT, NET, and DAT are of conspicuous medical relevance because they are the targets of the approved (e.g., antidepressants and methylphenidate) and illicit drugs (e.g., cocaine and amphetamines). It has long been appreciated that the membrane density of transporter molecules is also regulated by their endocytic removal from the cell surface (Qian *et al*, 1997; Daniels & Amara, 1999; Melikian & Buckley, 1999; Melikian, 2004). The biological relevance of monoamine transporter internalization is not fully understood. However, transporter internalization can be triggered by their cognate substrate, amphetamines, antidepressants, or the protein kinase C activator phorbol 12‐myristate 13‐acetate (PMA) (Anderson & Horne, 1992; Daniels & Amara, 1999; Saunders *et al*, 2000; Jayanthi *et al*, 2005; Lau *et al*, 2008; Jorgensen *et al*, 2014). In addition, monoamine transporters also undergo constitutive internalization (Loder & Melikian, 2003; Rahbek‐Clemmensen *et al*, 2014).

During clathrin‐mediated endocytosis, a cage of clathrin triskelia locally assembles around the inner leaflet of the plasma membrane and stabilizes the formation of spherical pits, which ultimately pinch off as vesicles on the intracellular side. Internalization of DAT depends on clathrin (Sorkina *et al*, 2005). SERT endocytosis is less well understood. Clathrin‐mediated endocytosis requires adaptor protein‐2 (AP2), which links endocytic cargo to the clathrin coat. Hence, many cargos (e.g., the transferrin receptor) provide short‐amino‐acid motifs, which directly contact the adaptor protein (Trowbridge *et al*, 1993). Some other cargos rely on additional interacting proteins, which establish contact with AP2 and other components of the endocytic machinery. The most prominent examples are the β‐arrestins, which support the recruitment of G‐protein‐coupled receptors to AP2/clathrin (Laporte *et al*, 1999; Oakley *et al*, 1999). The carboxyl terminus (C‐terminus) of DAT harbors a sequence (^587^FREKLAYAIA^596^), which is required for transporter endocytosis (Holton *et al*, 2005; Sorkina *et al*, 2005). However, this sequence does not conform to a canonical AP2 interaction site. This raises the possibility that DAT and related monoamine transporters rely on one or several auxiliary proteins, which support the interaction with AP2 and the clathrin coat in a manner reminiscent of arrestin‐2 and ‐3.

During the cell cycle, a protein complex—referred to as the spindle assembly checkpoint (SAC)—safeguards correct chromosomal attachment to the mitotic spindle by sequestration of the anaphase‐promoting complex/cyclosome (APC/C) co‐factor CDC20. The small SAC protein MAD2 and its main interactors BubR1 and p31^comet^ play pivotal roles in SAC (Kops *et al*, 2020). Previous research found that, surprisingly, BubR1 interacted with β2‐adaptin (a subunit of AP2) (Cayrol *et al*, 2002) and MAD2 interacted with the C‐terminus of the insulin receptor (O'Neill *et al*, 1997). More recently, the interaction between MAD2 and BubR1 was shown to fulfill a moonlighting function as an endocytic mediator by connecting the insulin receptor (IR) to the clathrin coat (Choi *et al*, 2016, 2019): upon engagement of insulin, insulin receptor‐bound MAD2 releases the inhibitory‐binding partner p31^comet^ and recruits BubR1, which delivers the insulin receptor to AP2/clathrin‐containing structures.

Interestingly, MAD2 is expressed in neuronal tissue of the human brain (O'Neill *et al*, 1997; Uhlen *et al*, 2005, 2015; Yu *et al*, 2020). This raises the question of the role of a cell cycle protein in post‐mitotic cells. In the present study, we show that SERT interacts with the SAC proteins MAD2, BubR1, and p31^comet^; MAD2 binds to a MAD2 interaction motif in the transporter C‐terminus. This binding is contingent on the “closed” conformation of MAD2. All three SAC proteins are expressed in serotonergic dorsal raphe neurons. Depletion of MAD2 in cells disrupted the interaction between SERT and BubR1/AP2 and decreased transporter endocytosis. Accordingly, lentivirus‐mediated depletion of MAD2 in cultured serotonergic rat neurons reduced endocytosis of SERT.

## Results and Discussion

### Neurotransmitter transporters contain putative C‐terminal MAD2 interaction motifs (MIMs)

**Figure 1:**
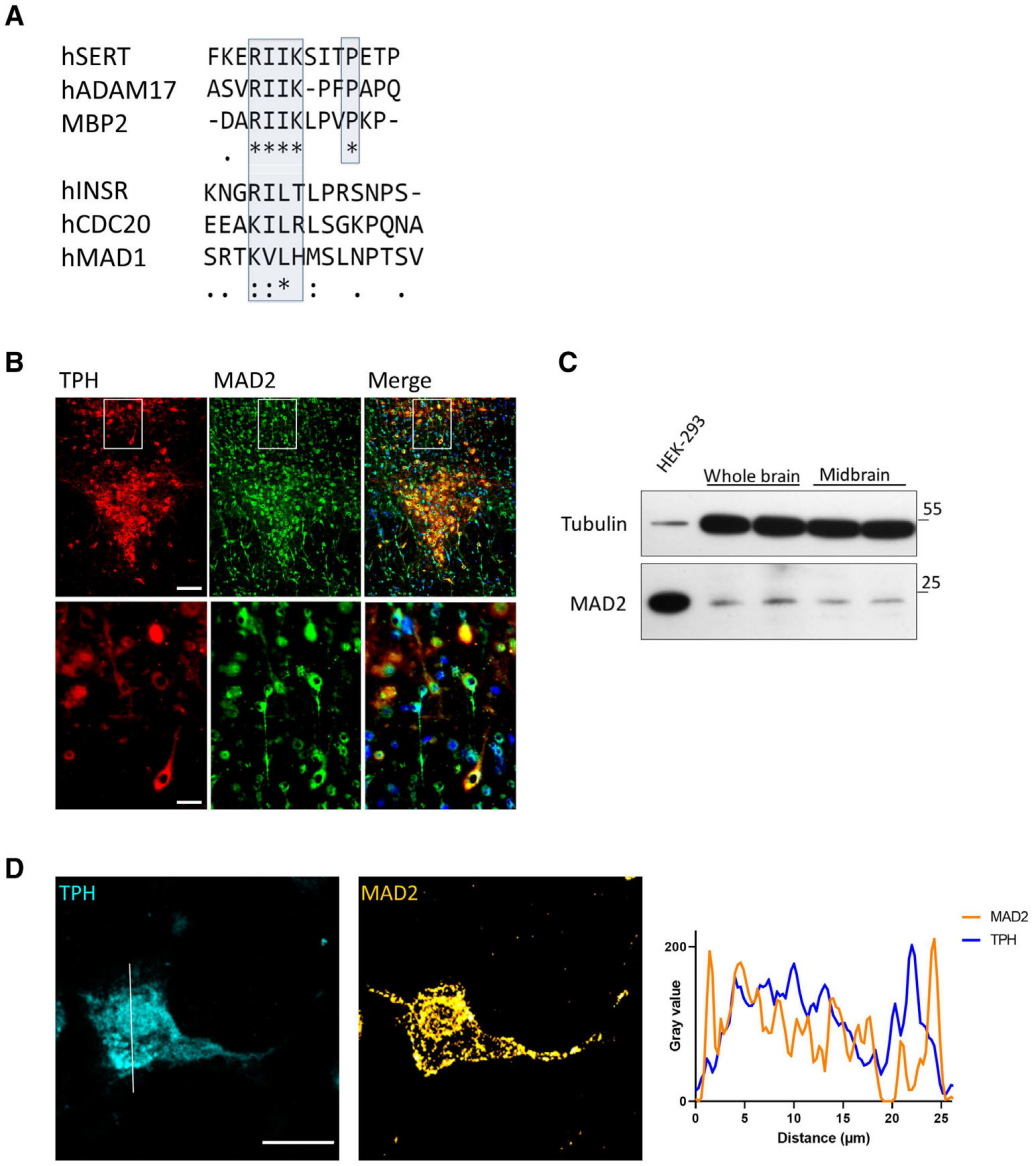
The serotonin transporter C‐terminus harbors a potential MAD2 interaction motif (MIM). MAD2 protein expression in dorsal raphe neurons Clustal Omega alignment of the SERT C‐terminus with previously described MAD2‐interacting proteins. The putative SERT‐MIM and previously described MIMs are highlighted by blue boxes; (*)—fully conserved residue; (:)—residues of strongly similar properties; (.)—residues of weakly similar properties.Cryosections were subjected to immunofluorescence microscopy using appropriate excitation wavelengths and emission filters. White boxes indicate the magnified area in the lower panel. All images were taken as “multiple image alignments.” Scale bars represent 100 and 25 μm in the upper and lower panel, respectively.Whole‐brain and midbrain lysates of adult mice were prepared as outlined under “Materials and Methods.” Total protein (20 μg) was immunoblotted for MAD2 or α‐Tubulin and compared to HEK‐293 cell lysates.Z‐stacks of individual TPH^+^/MAD2^+^ positive neurons were generated by confocal microscopy. Intensity profiles of an average projection were generated using ImageJ/Plot Profile software. Scale bar represents 20 μm. Source data are available online for this figure.

**Figure EV1:**
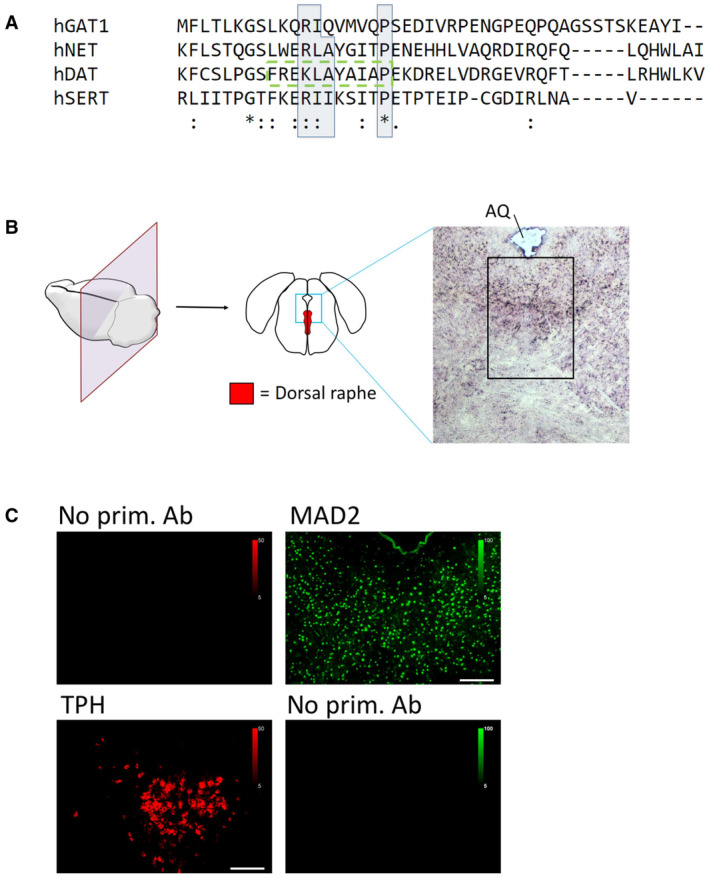
Conservation of transporter C‐terminal MIMs and control stainings for antibodies The C‐termini of indicated transporters were aligned using Clustal Omega software. Conserved residues of candidate MIMs are highlighted by blue boxes; (*)—fully conserved residue; (:)—residues of strongly similar properties; (.)—residues of weakly similar properties. The previously described endocytic motif in DAT is highlighted by the green dashed box.Cryosections of mouse brain were prepared covering the dorsal raphe and stained with hematoxylin/eosin. The black box indicates the approximate area of immunofluorescence images in Figs 1B and 3A and B. AQ = cerebral aqueduct.Mouse brain sections were subjected to immunofluorescence microscopy as described under “Materials and Methods,” with or without polyclonal antibodies for either TPH or MAD2, as indicated. Scale bars represent 100 μm.

The N‐ and C‐termini of SLC6 transporters are more divergent than their hydrophobic cores, but neurotransmitter transporters have conserved elements in their C‐termini (Fig EV1A). MAD2 interaction motifs (MIMs) consist of a core motif of two hydrophobic residues, one basic residue (Arg or Lys) and a third hydrophobic residue followed by one or several prolines (Luo *et al*, 2002). Interestingly, C‐termini of monoamine transporters harbor sequences that resemble MIMs (Fig EV1A, blue boxes). Furthermore, in DAT, this motif resides in a sequence previously shown to regulate transporter internalization (Holton *et al*, 2005; Sorkina *et al*, 2005; Boudanova *et al*, 2008) (Fig EV1A, green dashed box). Importantly, the SERT C‐terminus contains a sequence that is consistent with a candidate MIM. We further corroborated this conjecture by aligning this sequence of SERT with MIMs of several canonical MAD2 binders (Fig 1A). In fact, the sequence in SERT was identical in all critical residues to that of the canonical MAD2 binder ADAM17 (TACE) (Nelson *et al*, 1999; Choi *et al*, 2016) and of the (non‐natural) peptide ligand MBP2 (MAD2‐binding peptide 2) (Luo *et al*, 2002). We, therefore, concluded that SERT harbored a candidate MAD2‐binding motif.

### MAD2 expression in mouse raphe neurons

It is a prerequisite that SERT and MAD2 are endogenously co‐expressed in the same cell if the putative interaction of SERT is of physiological relevance. Accordingly, we examined serotonergic neurons of mouse dorsal raphe nuclei for the presence of MAD2. We identified serotonergic neurons by staining cryosections of mouse brain covering the dorsal raphe (Fig EV1B) by double immunofluorescence with antibodies against tryptophan hydroxylase (TPH) and MAD2 (Fig 1B) (for primary antibody controls, see Fig EV1C). This approach verified the existence of MAD2 within TPH‐positive dorsal raphe neurons. It is obvious that MAD2 was also found in TPH‐negative cells throughout the whole extent of the section. This observation suggests that the protein fulfills tasks in the brain other than a putative interaction with the serotonin transporter. In addition, we confirmed previous data (O'Neill *et al*, 1997) that MAD2 can be found in brain by immunoblotting lysates of mouse whole brain and midbrain. In fact, an immunoreactive band migrating at the same position as MAD2 from HEK‐293 cells was present in extracts prepared from both the whole brain and midbrain (Fig 1C).

Furthermore, the localization of MAD2 in TPH‐positive neurons was explored by laser scanning confocal microscopy along the *z*‐axis (Fig 1D): MAD2 was found in punctate structures in the cytosol and lining the cellular membrane. This distribution is consistent with the possibility that MAD2 interacts with neuronal membrane proteins. As expected, a fraction of MAD2 was located in the nucleus, possibly due to passive diffusion through the nuclear pore complex (NPC) and/or association with the NPC protein Tpr (Lee *et al*, 2008).

### Binding of MAD2 to the candidate C‐terminal MIM

**Figure 2:**
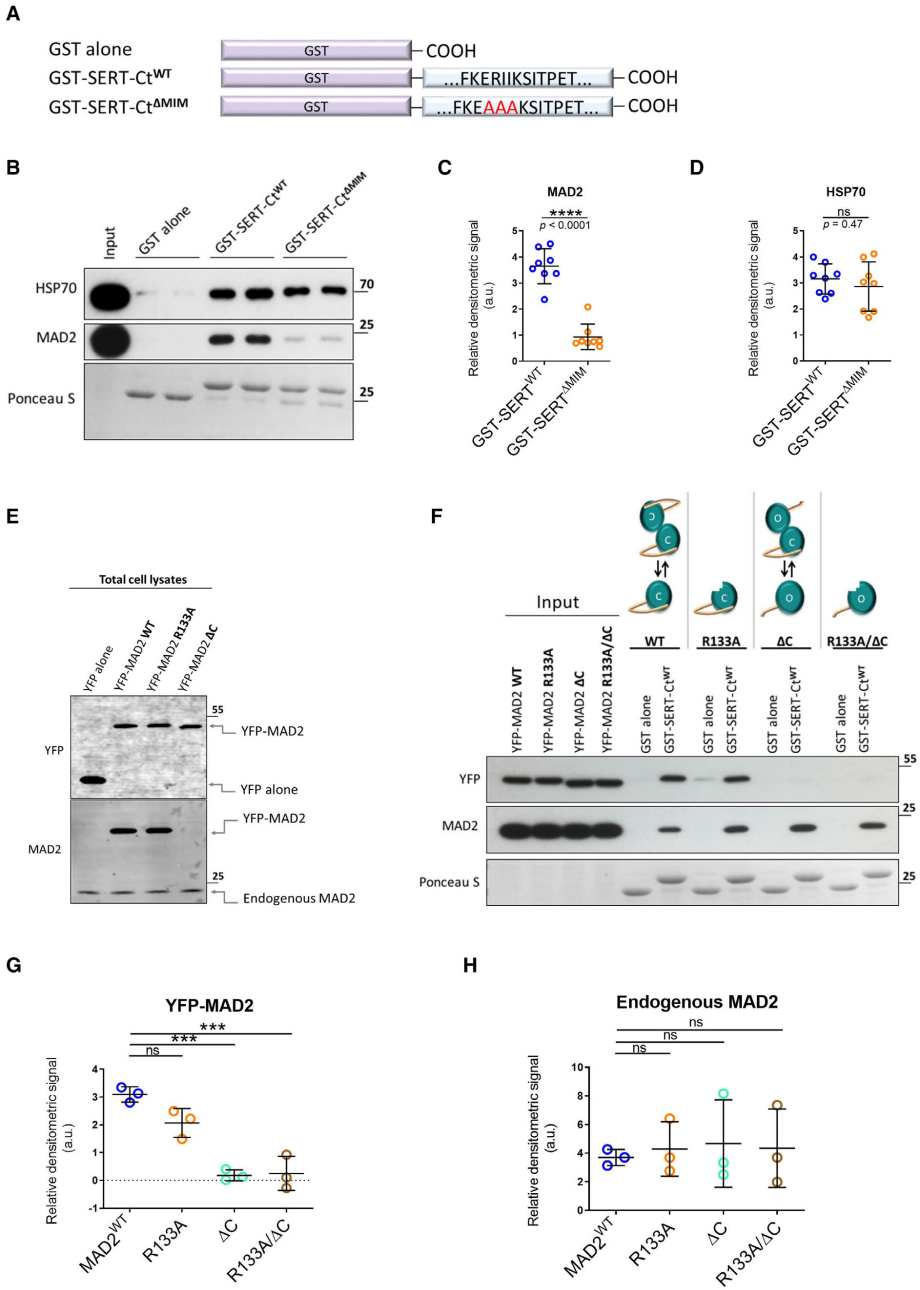
“Closed” MAD2 interacts with monoamine transporter C‐termini at MAD2 interaction motifs AGlutathione S‐transferase (GST)‐tagged serotonin transporter C‐terminus and the derivative RII‐AAA mutant were purified and used in GST‐protein‐based interaction assays.B–DGST pull‐down experiments were conducted in duplicates using the indicated GST fusion proteins. Eluates (30%) were subjected to immunoblotting together with an aliquot (1%) of the input. Blots were stained with Ponceau S, and MAD2 was detected by immunoblotting. Densitometric signals were quantified using ImageJ software. Results were compared using unpaired two‐tailed *t*‐tests; ****P < 0.0001, ns—not significant; exact *P*‐values are indicated; error bars represent SD of eight independent biological replicates (*n* = 8).ETotal cell lysates (5 μg) of indicated transfections were subjected to immunoblotting, using antibodies against YFP and MAD2.F–HYFP‐hMAD2 constructs were generated as outlined under “Materials and Methods.” Whole‐cell lysates of transfected HEK‐293 cells were used for GST pull‐down experiments. One‐way ANOVA with Dunnett's multiple *post hoc* comparison; ***P < 0.001, ns—not significant; error bars represent SD of three independent biological replicates (*n* = 3). Source data are available online for this figure.

We verified an interaction of the C‐terminus of SERT with MAD2 by employing glutathione S‐transferase (GST) fused to the C‐terminus of SERT (Fig 2A). GST and GST fusion proteins immobilized on glutathione‐conjugated beads were incubated with whole‐cell lysates prepared from HEK‐293 cells. The immobilized material was analyzed for the presence of MAD2 by immunoblotting. MAD2 was retained by GST fused to the SERT C‐terminus (GST‐SERT‐Ct) but not by GST alone (Fig 2B).

We examined, if the candidate MIM mediated this interaction, by replacing the Arg‐Ile‐Ile sequence with three alanine residues (RII‐AAA) to generate GST‐SERT‐Ct^ΔMIM^ (Fig 2A, third construct). In fact, the mutation resulted in a significant drop in MAD2 binding (Fig 2B and C). HSP70 binds to a segment in the SERT C‐terminus preceding the MIM (El‐Kasaby *et al*, 2014). Thus, as a control, we compared retrieval of HSP70 by wild type and mutated C‐termini: equivalent amounts were retrieved with both constructs (Fig 2B and D). This observation ruled out that the mutation of the C‐terminus compromised its ability to engage in protein–protein interactions by a non‐specific effect.

### The closed conformation of MAD2 is the interacting state

Mitotic arrest deficient‐2 can adopt two thermodynamically stable conformations, referred to as open and closed (oMAD2 and cMAD2) (Yu, 2006; Mapelli *et al*, 2007). In the latter, the C‐terminal region of MAD2 embraces the MAD2 interaction partner in a “safety belt”‐like manner (Sironi *et al*, 2002). Previously described MAD2 interaction partners (e.g., CDC20, MAD1) and the insulin receptor bind to the closed conformation of MAD2 (Luo & Yu, 2008; Choi *et al*, 2016). It was reasonable to surmise that the interaction between SERT and MAD2 was based on the same principle. We verified this prediction by generating YFP‐tagged MAD2 variants. The YFP‐tag was chosen for two reasons: (i) it adds about 25 kDa to the size of MAD2 (final MW ~48 kDa). Hence, the resulting tagged protein can readily be distinguished from endogenous MAD2 (~23 kDa) on the same immunoblot. (ii) The commercially available MAD2 antibody is directed against the C‐terminus and does not recognize a C‐terminal truncation mutant of MAD2 (Fig 2E). MAD2 requires an intact C‐terminus to adopt the closed conformation. This strict requirement allows for generating a constitutively open MAD2 by deleting the last 10 amino acids (MAD2^ΔC^) (Luo *et al*, 2000). All YFP‐MAD2 constructs can be detected by immunoblotting with an anti‐GFP antibody (Fig 2E).

Closed MAD2 dimerizes with both, another cMAD2 (symmetric dimer) and oMAD2 (asymmetric dimer), whereas ligand‐bound cMAD2 can only form asymmetric dimers (Luo & Yu, 2008; Yang *et al*, 2008). Mutation of arginine at position 133 to alanine (MAD2^R133A^) renders MAD2 dimerization deficient (Sironi *et al*, 2001). This is useful to investigate if binding of MAD2 substrates depends on MAD2 dimerization. MAD2^ΔC/R133A^ was also generated to rule out that open MAD2^ΔC^ indirectly associates with the transporter C‐terminus merely by dimerization with ligand‐bound endogenous MAD2.

YFP‐tagged versions of MAD2 were transiently expressed in HEK‐293 cells and lysates thereof were subjected to GST pull‐down experiments. As evident from Fig 2F and G, equivalent levels of wild‐type YFP‐MAD2 and monomeric YFP‐MAD2^R133A^ were retrieved by GST‐SERT‐Ct. This indicates that the dimeric nature of MAD2 is immaterial for binding SERT. In contrast, both YFP‐MAD2 constructs lacking the C‐terminus failed to bind to GST‐SERT‐Ct (Fig 2F and G). Thus, the closed conformation of MAD2 is required to support its interaction with SERT. In addition, the observations indicate that MAD2 binds to SERT as a monomer because MAD2^ΔC^ was not pulled down concomitantly with endogenous MAD2. Under all conditions, similar levels of endogenous MAD2 were retrieved by GST‐SERT‐Ct (Fig 2F and H).

### BubR1 and p31^comet^ are expressed in raphe neurons

**Figure 3:**
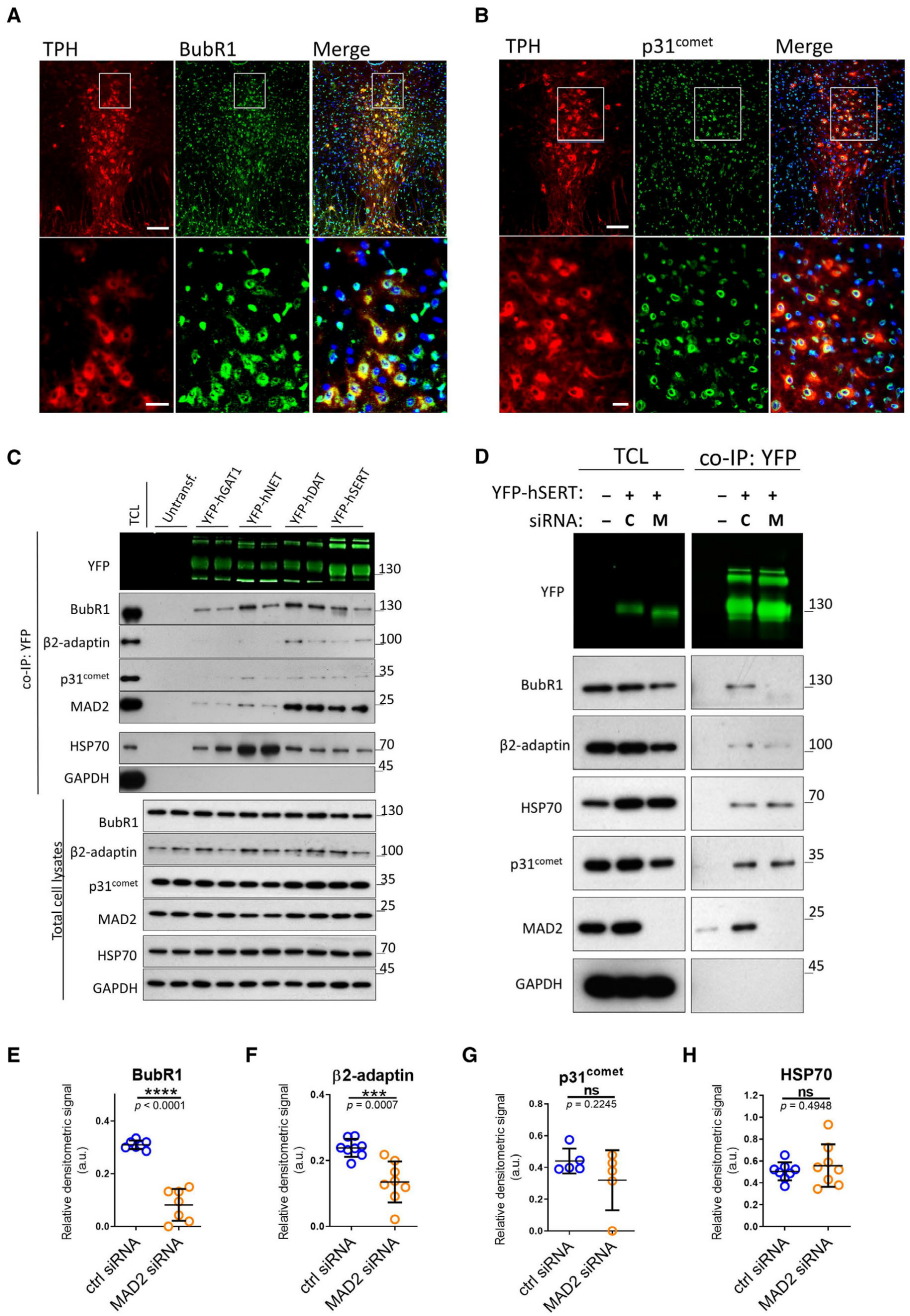
Neurotransmitter transporters associate with β2‐adaptin and the SAC proteins MAD2, p31^comet^, and BubR1, which are expressed in raphe nuclei. Effect of MAD2 depletion on SERT interactions A, BImmunofluorescence of p31^comet^ and BubR1 in mouse dorsal raphe. White boxes indicate the magnified sections in the lower panels. Scale bars represent 100 and 25 μm in the upper and lower panels, respectively.CCo‐immunoprecipitation experiments using lysates from HEK‐293 cells stably expressing the indicated YFP‐tagged transporters. Eluates (20%) as well as 0.1% total cell lysate were analyzed by immunoblotting. The immunoblot for mouse anti‐β2‐adaptin was subsequently used for incubation with rabbit anti‐GFP primary and IRDye 800CW anti‐rabbit secondary antibody. IRDye signals were detected using the LI‐COR Odyssey CLx imaging system. Densitometric signals were quantified using ImageJ software.D–HHEK‐293 cells stably expressing YFP‐hSERT were transfected with indicated siRNAs (“C”—ctrl siRNA; “M”—MAD2 siRNA) and subjected to co‐immunoprecipitation. All signals were normalized to YFP‐SERT. Results were compared using unpaired two‐tailed *t*‐tests; ***P < 0.001, ****P < 0.0001, ns—not significant; calculated *P*‐values are indicated; error bars represent SD of seven (BubR1), eight (β2‐adaptin), five (p31^comet^), and eight (HSP70) independent biological replicates. Source data are available online for this figure.

As part of the spindle assembly checkpoint, MAD2 interacts with numerous proteins. When bound to the insulin receptor, MAD2 recruits the SAC proteins BubR1 and p31^comet^ (Choi *et al*, 2016). We verified the expression of BubR1 and p31^comet^ in dorsal raphe neurons. Both proteins were detected by immunofluorescence microscopy in TPH^+^ serotonergic raphe neurons. In addition—and similar to MAD2—BubR1 and p31^comet^ were not confined to TPH^+^ neurons: they were also seen in the area adjacent to the raphe nucleus (Fig 3A and B). BubR1 was distributed uniformly in the cytosol of TPH^+^ neurons, whereas p31^comet^ was enriched in a region surrounding the nucleus of TPH^+^ neurons, but it was also detectable in the cytosol.

### MAD2 recruits SAC proteins and AP2 to SERT

**Figure EV2:**
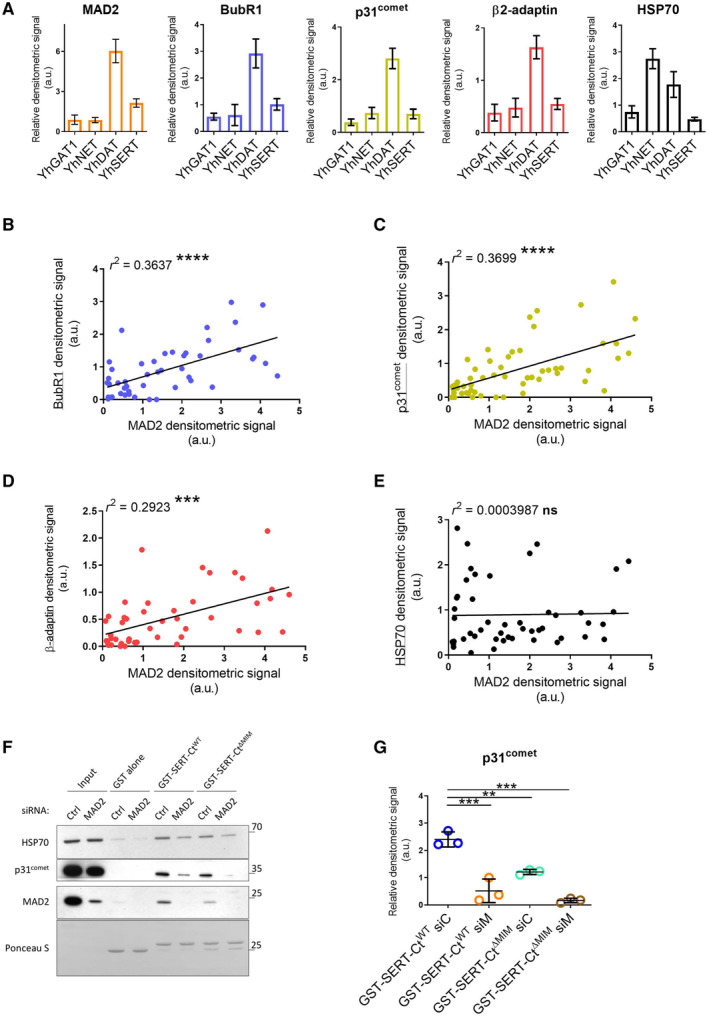
Association of full‐length transporters with BubR1, β2‐adaptin, and p31^comet^ correlates with MAD2 association ADensitometric signals in Fig 3C/co‐IP:YFP were quantified using ImageJ and normalized to their respective transporter signal intensities; error bars represent SEM. Data derive from seven (MAD2), six (BubR1), seven (p31^comet^), six (β2‐adaptin), and six (HSP70) independent biological replicates.B–EScatter plots for the co‐IP of indicated proteins (*y*‐axis) against the co‐IP of MAD2 (*x*‐axis) (cf. Fig 3C); *r* ^2^ indicates the squared linear correlation coefficient; ***P < 0.001, ****P < 0.0001, ns—not significant. Data derive from six (BubR1), seven (p31^comet^), six (β2‐adaptin), and six (HSP70) independent biological replicates.F, GHEK‐293 cells were transfected with the indicated siRNAs and cell lysates were used for GST pull‐down experiments using the indicated GST proteins. One‐way ANOVA with Dunnett's multiple *post hoc* comparison; **P < 0.01, ***P < 0.001; error bars represent SD of three independent biological replicates.

We examined binding of SAC proteins to related neurotransmitter transporters of the solute carrier‐6 (SLC6) family by conducting co‐immunoprecipitation experiments with YFP‐tagged variants of SERT, DAT, NET, and GAT1 (GABA transporter 1). MAD2 was co‐immunoprecipitated with all transporters, albeit at very different amounts (Figs 3C and EV2A). Similarly, variable levels of BubR1 and p31^comet^ were found in the immunoprecipitates. We used complex formation between HSP70 and newly synthesized transporters as a positive control (El‐Kasaby *et al*, 2014, 2019; Kasture *et al*, 2016; Asjad *et al*, 2017): HSP70 was co‐immunoprecipitated by each transporter. As a negative control, we used the abundant cytosolic protein glyceraldehyde 3‐phosphate dehydrogenase (GAPDH): this protein was not detected in the immunoprecipitates. Hence, we conclude that the observed co‐immunoprecipitation of MAD2, BubR1, and p31^comet^ with the transporters reflected a specific association.

If MAD2 facilitates the interaction of the spindle assembly checkpoint (SAC) proteins BubR1 and p31^comet^ (in a manner analogous to the insulin receptor), their levels in co‐immunoprecipitates are predicted to be correlated. This was the case: we observed a statistically significant correlation between the amount of co‐immunoprecipitated p31^comet^ and BubR1 and the amount of transporter‐associated MAD2 (Fig EV2B and C). Furthermore, a correlation was also seen for the co‐immunoprecipitated amount of the AP2 subunit β2‐adaptin and MAD2 (Fig EV2D). In contrast, the levels of co‐immunoprecipitated HSP70 did not show any correlation with the amount of MAD2 (Fig EV2E). Taken together, these observations confirm that neurotransmitter transporters—in particular DAT and SERT—interact with proteins of the spindle assembly checkpoint. The quantitative correlation of co‐immunoprecipitated BubR1, p31^comet^, and AP2 with MAD2 suggests a complex consisting of SAC proteins and proteins of the clathrin endocytic machinery, which may drive endocytosis of neurotransmitter transporters. In this case, elimination of MAD2 ought to cause disassembly of this complex. In fact, siRNA‐mediated knockdown of MAD2 resulted in a substantial reduction of BubR1 co‐immunoprecipitated with SERT (Fig 3D and E) and in a drop by about 50% of β2‐adaptin (Fig 3D and F). The levels of p31^comet^ tended to be reduced but the difference did not reach statistical significance (Fig 3D and G). MAD2 depletion did not affect the interaction between SERT and HSP70 (Fig 3H).

We alternatively examined the consequences of MAD2 knockdown for binding of p31^comet^ in a GST pull‐down experiment (Fig EV2F): siRNA‐mediated depletion of MAD2 from the total cell lysate alone reduced the amount of p31^comet^, which was retained by the C‐terminus of SERT. This effect was further enhanced by the mutation of the MAD2 interaction motif: negligible levels of p31^comet^ were retrieved from MAD2‐depleted lysates by GST‐SERT‐Ct^ΔMIM^ (Fig EV2F and G). Hence, we conclude that MAD2 supports the recruitment of BubR1, p31^comet^, and the endocytic adaptor protein 2 (AP2) to the serotonin transporter.

### MAD2 depletion in HEK‐293 cells blocks SERT accumulation in endocytic compartments

**Figure 4:**
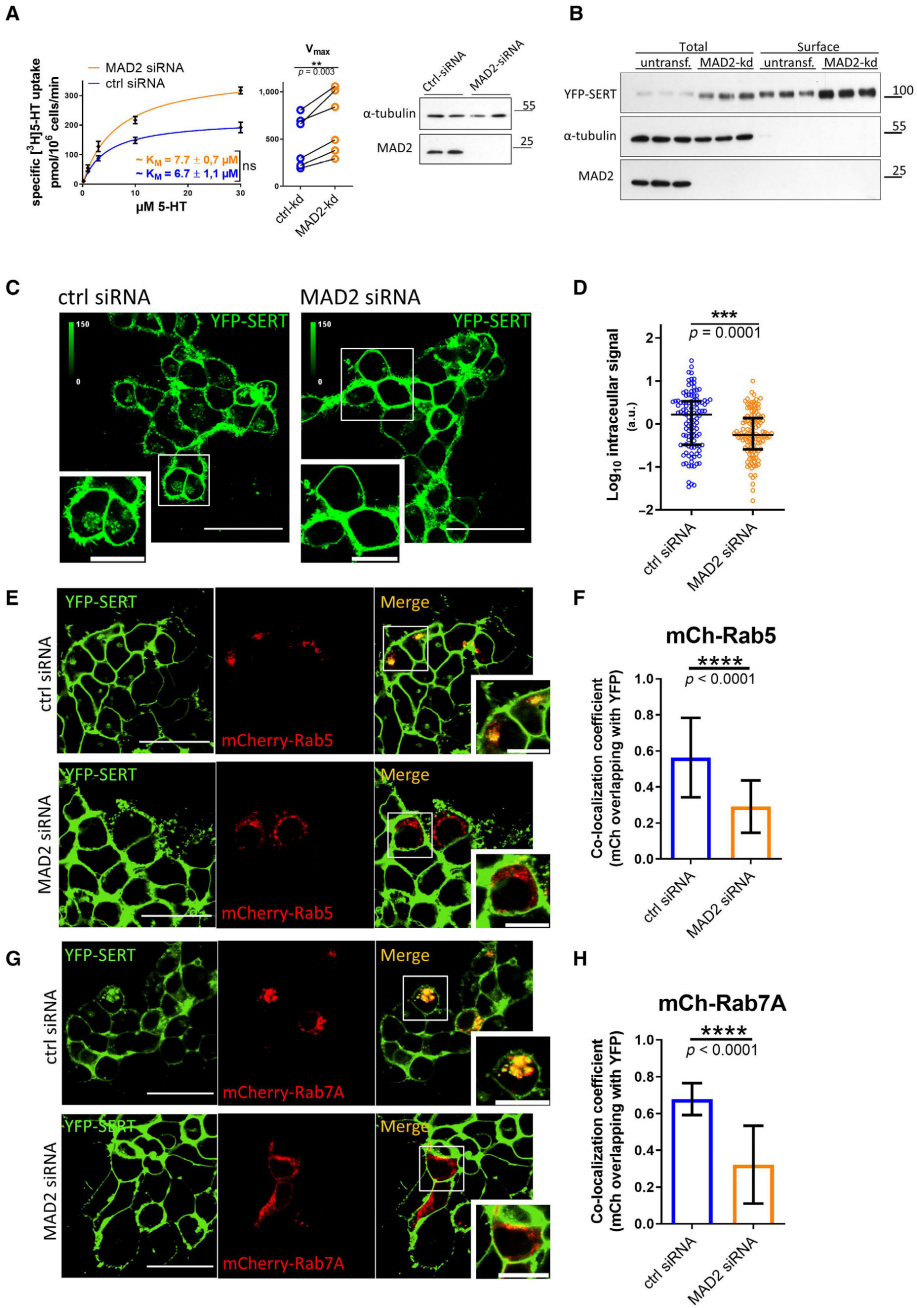
MAD2 depletion concentrates SERT at the surface of HEK‐293 cells and impairs transporter endocytosis ARadioactive substrate uptake was conducted as described under “Materials and Methods.” K_M_ and V_max_ were determined by fitting the data to the equation of a rectangular hyperbola. Representative curves are shown. Error bars represent SEM. For comparison of V_max_, paired two‐tailed *t*‐test was conducted; **P < 0.01, ns—not significant. Data are derived from six independent biological replicates. Lysates were prepared from an aliquot of the cells, which were used for the uptake assay, and subjected to immunoblotting to verify MAD2 knockdown. Immunodetection of α‐tubulin was used as a loading control.BCell surface biotinylation using HEK‐293 cells stably expressing YFP‐hSERT was performed as described under “Materials and Methods.” Whole‐cell lysates and streptavidin‐conjugated fractions (i.e., surface protein) were subjected to immunoblotting.C, DHEK‐293 cells stably expressing YFP‐hSERT were transfected with the indicated siRNAs, imaged, and analyzed as described under “Materials and Methods.” Representative maximum‐intensity projections of Z‐stacks are shown. Scale bars represent 50 and 20 μm in full‐size images and zoomed areas, respectively. Intracellular SERT signal was measured using ImageJ software. The dot plot in Panel (D) shows values (presented as Log_10_ to account for the large variation in the signal) from individual cells (Ctrl siRNA *n* = 112 cells; MAD2 siRNA *n* = 116 cells) deriving from three independent biological replicates, the median, and the interquartile range. The statistically significant difference was assessed by an unpaired two‐tailed *t*‐test; ***P < 0.001.E–HHEK‐293 cells stably expressing YFP‐hSERT were transfected with the indicated siRNA, subsequently co‐transfected with plasmids encoding mCherry‐Rab5 (Panel E) or mCherry‐Rab7A (Panel G), and imaged as described under “Materials and Methods.” Manders' overlap coefficients for the fraction of mCherry‐overlapping YFP were calculated for multiple individual images using ImageJ/JACoP. Number of analyzed images: mCh‐Rab5/ctrl‐siRNA = 38; mCh‐Rab5/MAD2‐siRNA = 33 (Panel F); mCh‐Rab7A/ctrl‐siRNA = 13; and mCh‐Rab7A/Mad2‐siRNA = 14 (Panel H). Statistically significant differences were assessed by unpaired two‐tailed *t*‐test; ****P < 0.0001. Error bars represent SD of three independent biological replicates (*n* = 3). Scale bars represent 50 and 20 μm in full‐size images and zoomed areas, respectively. Source data are available online for this figure.

Based on the data summarized above, we posited a role of MAD2 in driving endocytosis of SERT. This conjecture was explored by depleting MAD2 by siRNA‐mediated knockdown in YFP‐SERT‐expressing HEK‐293 cells: we analyzed the effect by measuring serotonin uptake and by visualizing the subcellular localization of SERT. The velocity of substrate uptake correlates with the surface presence of the transporter. Hence, substrate uptake is predicted to be enhanced, if MAD2‐depletion precludes constitutive endocytosis and thus results in the accumulation of SERT at the cell surface. In fact, MAD2 depletion increased the V_max_ (as a measure of surface transporter density) of serotonin uptake by about 40% (Fig 4A), whereas the K_M_ (as a measure of substrate affinity) was unaltered. Accordingly, we examined SERT total and surface levels in HEK‐293 cells by cell surface biotinylation. Immunoblotting revealed that, after siRNA‐mediated knockdown of MAD2, SERT levels were elevated in both detergent lysates and in the SERT pool, which was at the cell surface and thus accessible to the membrane‐impermeable biotinylation reagent (Fig 4B). Images captured by confocal microscopy revealed intracellular accumulation of SERT in control cells. This intracellular pool of SERT was significantly reduced in MAD2‐depleted cells (Fig 4C and D).

Serotonin transporter undergoes constitutive internalization and preferentially sorts to the late endosomal and lysosomal compartments in Cath.a‐differentiated (CAD) and HEK‐293 cells (Rahbek‐Clemmensen *et al*, 2014). Hence, intracellular SERT‐positive structures observed in HEK‐293 cells may be, in part, of endocytic origin. We identified these structures by transfecting YFP‐SERT‐expressing cells with plasmids encoding mCherry‐Rab5 (an early endosomal marker) and mCherry‐Rab7A (a late endosomal marker). In cells subjected to transfection with control siRNA, intracellular SERT clearly co‐localized with both endosomal markers (upper panels in Fig 4E and G). This was not observed in cells transfected with MAD2 siRNA (lower panels in Fig 4E and G). Quantification of co‐localization over multiple individual images confirmed that YFP‐SERT overlapped with Rab5 or Rab7A to a significantly lower extent in MAD2‐depleted cells (Fig 4F and H). This finding corroborates the concept that MAD2 participates in SERT endocytosis.

### MAD2 facilitates SERT endocytosis in the soma of serotonergic neurons

**Figure EV3:**
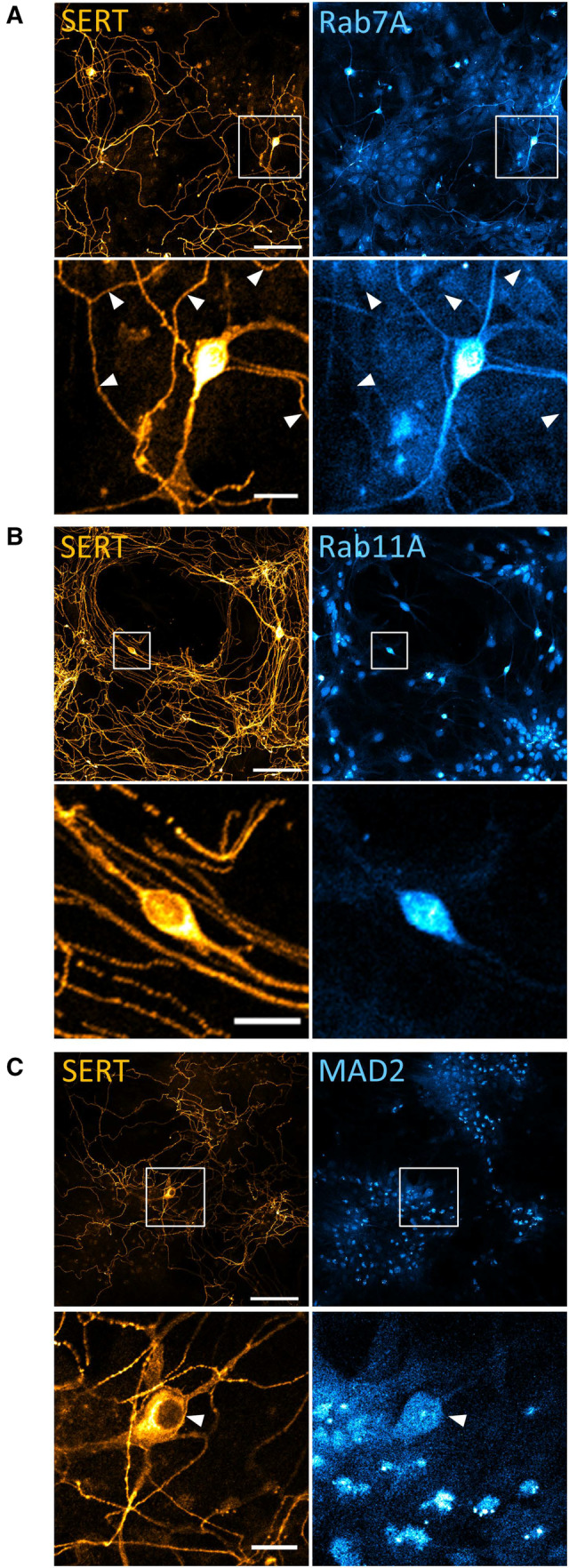
Localization of Rab11A, Rab7A, and MAD2 in cultured rat dorsal raphe neurons A–CPrimary rat dorsal raphe neurons were cultured as described under “Materials and Methods.” After 14 days, cells were fixed in acetone/methanol (1:1) and subjected to immunofluorescence with the indicated antibodies. Confocal images were captured on a Nikon A1 laser scanning confocal microscope at 20× magnification. White boxes in the merged image indicate zoomed area in the lower panels. White arrowheads specify serotonergic extensions, as Rab7A was also detectable in non‐serotonergic neurite extensions. Scale bars represent 100 and 20 μm for 20× image and zoomed area, respectively.

Next, we examined the role of MAD2 in endocytosis of endogenous SERT in cultured rat dorsal raphe neurons. Two lines of evidence indicated that only the neuronal soma qualified as the putative compartment of MAD2‐mediated SERT endocytosis: (i) immunofluorescence staining demonstrated that, in cultured serotonergic neurons, the endosomal markers Rab7A and Rab11A were conspicuously concentrated in the soma and virtually undetectable in distant serotonergic neurite extensions (presumably representing axonal arborizations and dendrites) (Fig EV3A and B). (ii) MAD2 was also confined to the soma (Fig EV3C). Hence, in subsequent experiments, we focused on SERT trafficking in the neuronal soma.

**Figure 5:**
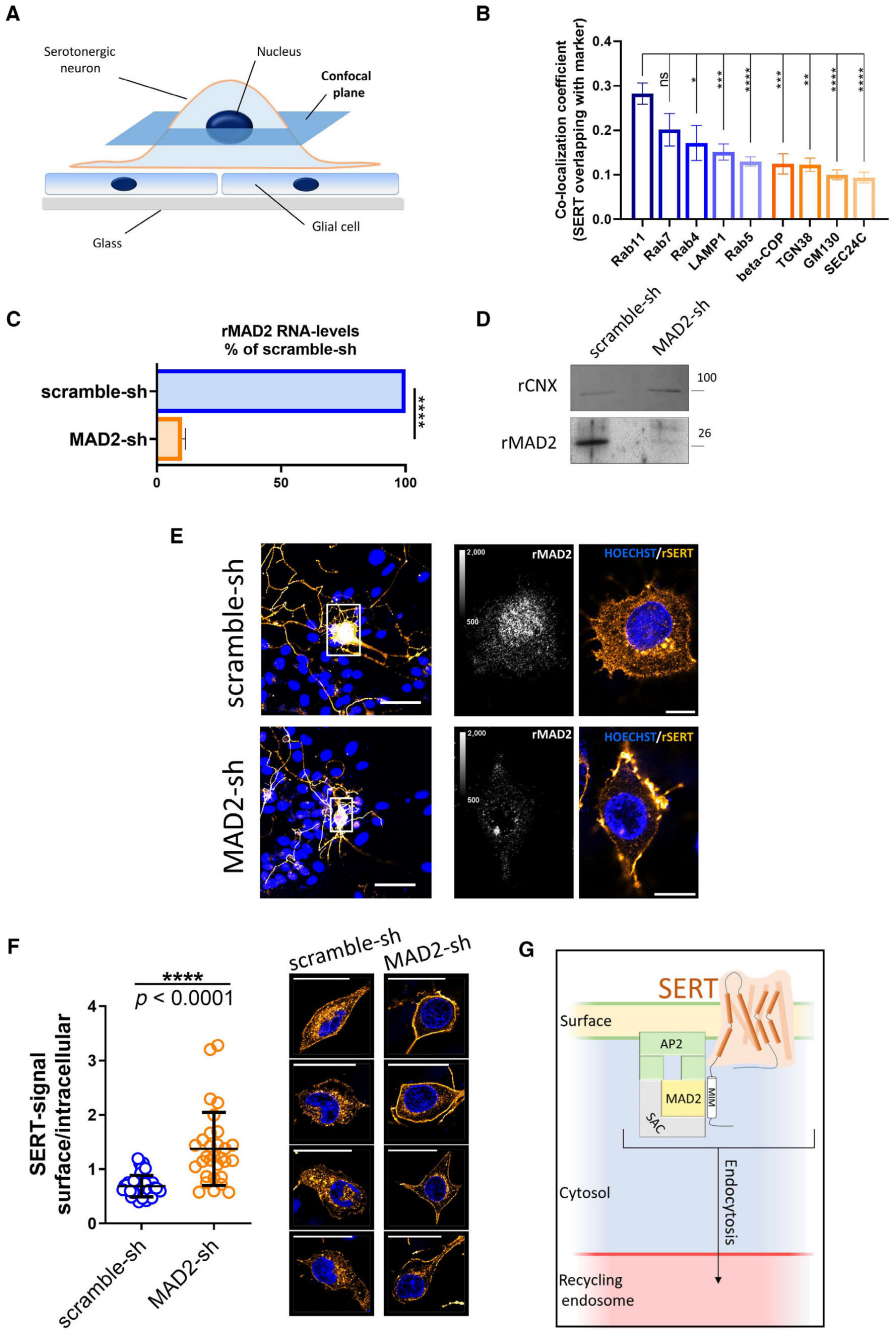
MAD2 depletion in dorsal raphe neurons prevents SERT endocytosis ASchematic representation of the confocal plane (dark blue) selected for visualizing immunoreactivity in somata of serotonergic neurons in Panel (E).BImmunofluorescence images of rat primary dorsal raphe neurons in Figs EV4 and EV5 were analyzed using ImageJ/JACoP software. Bars indicate Manders' overlap coefficients for the fraction of SERT overlapping with the denoted marker protein. One‐way ANOVA and Tukey's multiple‐comparisons test; *P < 0.05, **P < 0.01, ***P < 0.001, ****P < 0.0001. Number of analyzed cells: Rab11A *n* = 20; Rab7A *n* = 11; Rab4 *n* = 8; LAMP1 *n* = 12; Rab5 *n* = 14; beta‐COP *n* = 7; TGN38 *n* = 5; GM130 *n* = 11; SEC24C *n* = 11. Error bars represent SEM.C, DPrimary rat cortical glia cells were cultured as described under “Materials and Methods.” After 14 days, cells were infected with lentiviral particles encoding either scramble shRNA or shRNA directed against MAD2. Five days after infection, cells were subjected to quantitative PCR (*n* = 3 biological replicates) or immunoblotting (*n* = 2 biological replicates). For the latter, the ER resident protein calnexin was used as loading control. For Panel (C), ****P < 0.0001 in an unpaired two‐tailed *t*‐test. Error bars represent SD.EPrimary rat dorsal raphe neurons were cultured as described under “Materials and Methods.” After 14 days, cells were infected with lentiviral particles encoding either scramble shRNA or shRNA directed against MAD2. Five days after infection, cells were fixed in acetone/methanol (1:1) and subjected to immunofluorescence with the indicated primary and secondary antibodies. Confocal images were captured on a Nikon A1 laser scanning confocal microscope at either 20× (Fig 5E, left panel) or 60× (Fig 5E, right panel) magnification; representative images are shown. Due to imaging conditions, the optical section thickness is increased at 20× compared to 60× magnification. Calibration bars in images showing rMAD2 represent arbitrary fluorescence units. Scale bars represent 50 and 10 μm for 20× and 60× magnification, respectively.FSurface and intracellular SERT signal from scramble‐ and MAD2‐shRNA‐infected neurons (*n* = 40 and *n* = 30, respectively) was quantified within regions of interest (ROIs) using ImageJ software, and the resulting ratios were compared using an unpaired two‐tailed *t*‐test, the *P*‐value was below 0.0001 (*n* = 4 biological replicates). Error bars represent SD. The right part provides four additional representative images of both scramble‐shRNA and MAD2‐shRNA expressing serotonergic neurons (SERT: orange, nuclei: blue). Scale bars represent 30 μm.GModel of SERT endocytosis in somata of serotonergic neurons: MAD2 (in its closed conformation) binds a MAD2 interaction motif (MIM) in the SERT C‐terminus and forms a complex with other spindle assembly checkpoint proteins (SAC) and adaptor protein 2 (AP2). Subsequent endocytosis delivers SERT to the recycling endosome. Source data are available online for this figure.

**Figure EV4:**
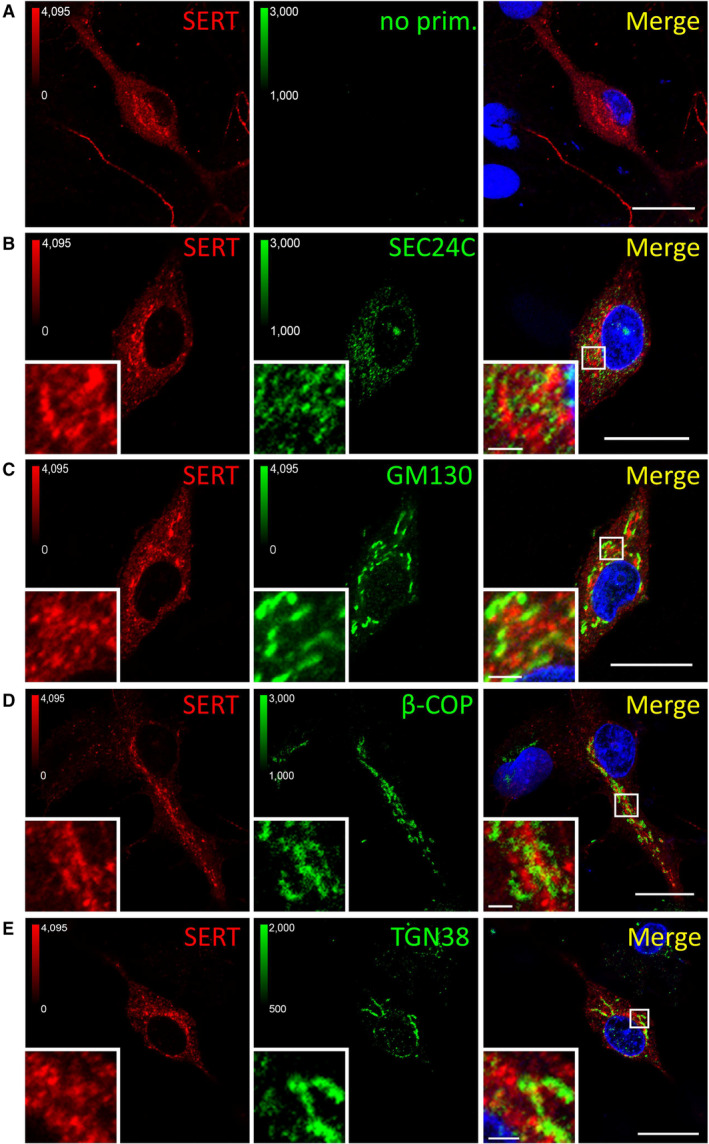
Co‐localization between SERT and markers of the secretory pathway A–EPrimary rat dorsal raphe neurons were cultured and fixed as for Fig EV3 and subjected to immunofluorescence with the indicated antibodies. Confocal images were captured on a Nikon A1 laser scanning confocal microscope at 60× magnification. White boxes in merged image indicate zoomed area in the lower left corner. Scale bars represent 20 μm in full‐size images and 2 μm in zoomed areas.

**Figure EV5:**
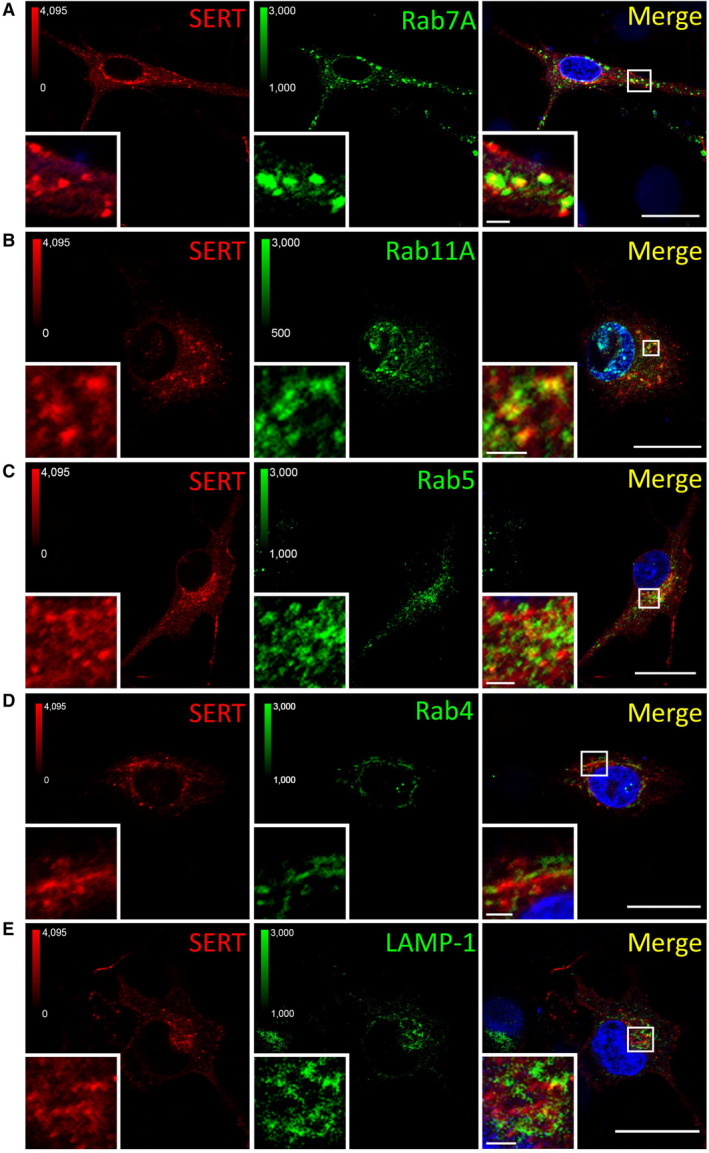
Co‐localization between SERT and endocytic markers A–EPrimary rat dorsal raphe neurons were cultured and imaged as in Fig EV4, using the indicated primary antibodies. Scale bars represent 20 and 2 μm in full‐size images and zoomed areas, respectively.

We visualized the distribution of SERT immunofluorescence by capturing confocal images at high magnification: when focusing on the z‐plane running through the midsection of serotonergic somata (Fig 5A), we found the bulk of SERT intracellularly (Fig EV4A) and barely co‐localized with markers of the secretory pathway, that is, SEC24C (ER exit sites, Fig EV4B), GM130 (*cis*‐Golgi, Fig EV4C), β‐COP (mainly not only *cis*‐ but also medial and *trans*‐Golgi, Fig EV4D), and TGN38 (*trans*‐Golgi, Fig EV4E). In contrast and consistent with previous findings (Rahbek‐Clemmensen *et al*, 2014), SERT co‐localized with Rab7A (late endosome, Fig EV5A), Rab11A (“long loop” recycling endosome, Fig EV5B), to a lesser degree with Rab5 (early endosome, Fig EV5C) and Rab4 (early and “short loop” recycling endosome, Fig EV5D), and barely with LAMP‐1 (lysosome, Fig EV5E). Taken together, these data show that, in rat dorsal raphe neurons, a substantial fraction of intracellular SERT resides in endocytic compartments (Fig 5B), which support recycling rather than degradation.

We addressed the role of MAD2 in endocytosis of endogenous SERT by infecting neuronal cultures with lentiviral particles encoding either MAD2‐specific or scrambled shRNA. The suitability of this knockdown approach was first verified in a culture of pure rat cortical glial cells: depletion of the target gene product was documented by quantitative PCR and immunoblotting, which demonstrated a substantial reduction in MAD2 mRNA (Fig 5C) and protein (Fig 5D) 5 days after infection. We stress that high amounts of glial cells are present in and required for neuronal cultures. Hence, at the level of the mass culture, it is not possible to quantify the effect of lentivirally encoded shRNA on the bulk level of neuronal MAD2. Subsequently, dorsal raphe cultures were virally infected and immunofluorescence of SERT and MAD2 was visualized by confocal microscopy (Fig 5E). At low magnification, SERT immunoreactivity was seen in the soma and neurite extensions. This distribution was comparable in cells infected to express scrambled shRNA and MAD2‐directed shRNA (Fig 5E, left‐hand top and bottom images). If the neuronal soma was examined at higher magnification, depletion of MAD2 was evident in neurons exposed to the lentivirus encoding the MAD2‐shRNA (cf. second set of images in Fig 5E). Like in uninfected neurons (Figs EV4 and EV5), somatic SERT was mainly present intracellularly in neurons, which had been infected with the lentivirus‐encoding scrambled shRNA (Fig 5E, third top image). In contrast, in serotonergic neurons, which had been depleted of MAD2, the bulk of SERT immunoreactivity was localized at the somatic surface (Fig 5E, third and fourth bottom images). The shift from the intracellular pool to the cell surface was statistically significant (Fig 5F). Given that intracellular SERT mainly derives from the endocytic system, this finding proves the role of MAD2 in endocytosis of somatic SERT in mammalian serotonergic neurons.

Serotonin transporter (and all related neurotransmitter transporters) exert their eponymous action, which is the retrieval of released neurotransmitter, in the presynaptic specialization. Recent evidence indicates that DAT undergoes regulated endocytic recycling in synaptic boutons (Kearney *et al*, 2023). However, it remains to be shown that presynaptic SERT does undergo endocytosis. If this is the case, internalization is very likely to be independent of MAD2 because all neurite extensions—and thus the axonal compartment—were devoid of MAD2 (Fig EV3). Hence, alternative pathways are likely involved. Previously, flotillin‐1 (Flot1) was shown to support regulated endocytosis of DAT required for endocytosis (Cremona *et al*, 2011). SERT also interacts with Flot1 (Reisinger *et al*, 2019). Flot1‐mediated endocytosis is clathrin independent (Glebov *et al*, 2006) (and thus presumably AP2/MAD2 independent). It is therefore conceivable that regulated endocytosis of presynaptic SERT also occurs via a Flot1‐dependent mechanism.

A detailed study on dopaminergic neurons showed that striatal and axonal DAT were predominantly localized at the plasma membrane and that endolysosomal compartments were virtually absent from striatal axons. In contrast, DAT was readily detectable in recycling endosomes of midbrain somatodendritic regions (Block *et al*, 2015). These data are in line with our results on SERT in cultured dorsal raphe neurons. The currently available evidence supports the conclusion that MAD2‐dependent regulation of SERT in the neuronal soma is of biological relevance. In fact, a recent study describes endocytosis as a key mechanism for the maintenance of neuronal polarity (Eichel *et al*, 2022): axonal surface proteins, which aberrantly diffuse into the soma, and conversely somatodendritic surface proteins, which enter the axon, are efficiently endocytosed to preserve the neuronal architecture. A mechanism is readily conceivable, by which MAD2 mediates endocytosis of somatic SERT followed by its trafficking to the recycling endosome (Fig 5G) in order to support its rerouting into the axon.

## Materials and Methods

### Animals

Pregnant Sprague–Dawley rats and C57BL/6 mice were provided by Charles River (Sulzfeld, Germany). Neonatal rat pups of either sex were killed by decapitation and adult mice were killed by decapitation under deep anesthesia with isoflurane in full accordance with all rules of the Austrian animal protection law (see http://www.ris.bka.gv.at/Dokumente/BgblAuth/BGBLA_2012_I_114/BGBLA_2012_I_114.pdf) and the Austrian animal experiment by‐laws (see http://www.ris.bka.gv.at/Dokumente/BgblAuth/BGBLA_2012_II_522/BGBLA_2012_II_522.pdf) which implement European law (DIRECTIVE 2010/63/EU; see http://eur‐lex.europa.eu/LexUriServ/LexUriServ.do?uri=OJ:L:2010:276:0033:0079:en:PDF) into Austrian law. The responsible animal welfare body is the Ethics Committee of the Medical University of Vienna for Research Projects Involving Animals.

### Protein sequence alignments

Protein sequences were aligned using Clustal Omega software (Sievers *et al*, 2011).

### Cell culture

Generation of HEK‐293 cells stably expressing N‐terminally YFP‐tagged human isoforms of SERT (YFP‐hSERT), DAT (YFP‐hDAT), and NET (YFP‐hNET) was described previously (Mayer *et al*, 2016; Niello *et al*, 2019). HEK‐293 cells stably expressing YFP‐hGAT1 were generated accordingly. In brief, cells were transfected with YFP‐hGAT1 using jetPRIME (114‐15, Polyplus‐Transfection). Stably expressing cells were selected in the presence of geneticin (250 μg ml^−1^) and enriched using fluorescence‐activated cell sorting. All cell lines were maintained in humidified atmosphere at 37°C 5% CO_2_ in antibiotic‐free high‐glucose Dulbecco's modified Eagle's medium (DMEM) supplemented with 10% fetal bovine serum. Generation of the plasmid‐encoding YFP‐hGAT1 is described in Section “Plasmids, molecular cloning, and mutagenesis.”

### Antibodies

#### Immunofluorescence

*Primary antibodies*: rabbit‐anti‐MAD2 (1:200; ab70385, abcam; or 1:500 ab70383, abcam); sheep‐anti‐tryptophan hydroxylase/TPH (1:150; ab32821, abcam); rabbit‐anti‐BubR1 (1:100; A300‐386A, Bethyl Laboratories Inc.); rabbit‐anti‐P31^comet^ (murine) (1:100; provided by Dr. Hongtao Yu; UT Southwestern, TX, USA); mouse‐anti‐SERT (H‐45) (1:20; raised and provided by Dr. Egon Ogris, Max Perutz Labs, Vienna, Austria (Montgomery *et al*, 2014)); rabbit‐anti‐SEC24C (1:100; provided by Dr. Randy Schekman, UC Berkeley, CA, USA); rabbit‐anti‐GM130 (1:200; ab52649, abcam); rabbit‐anti‐β‐COP (1:100; PA1‐061, Invitrogen); rabbit‐anti‐TGN38 (1:100; OST00228G‐500UG, Osenses) rabbit‐anti‐Rab7 (1:100; 9367S, Cell Signaling); rabbit‐anti‐Rab5 (1:100; 3547S; Cell Signaling); rabbit‐anti‐Rab11A (1:100; 71‐5300; Invitrogen); rabbit‐anti‐Rab4 (1:100; PA3912, Invitrogen); and rabbit‐anti‐LAMP1 (1:100, L1418; Sigma‐Aldrich). *Secondary antibodies*: Alexa Fluor 488 donkey‐anti‐rabbit IgG (1:500; A21206, Invitrogen); Alexa Fluor 555 donkey‐anti‐sheep IgG (1:500; A21436, Invitrogen); Alexa Fluor 555 donkey‐anti‐mouse (1:500; A31570, Invitrogen); and Alexa Fluor 647 goat‐anti‐rabbit (1:500; A32733, Invitrogen).

#### Immunoblotting

*Primary antibodies*: rabbit‐anti‐MAD2 (1:2,500; ab70385, abcam); mouse‐anti‐αTubulin DM1A (1:2,000, T9026‐100UL, Sigma‐Aldrich); mouse‐anti‐HSP70 (1:3,000; ab47455, abcam); rabbit‐anti‐GFP (1:5,000; ab290, abcam); rabbit‐anti‐BubR1 (1:1,500; A300‐386A, Bethyl Laboratories Inc.); mouse‐anti‐βActin (1:2,000; A0760‐40, USBiological Life Sciences); mouse‐anti‐Adaptin β (1:500; 610382, BDbiosciences); mouse‐anti‐GAPDH (1:2,000; sc‐47724, SantaCruz Biotechnology); rabbit‐anti‐p31^comet^ (human) (1:1,000; provided by Dr. Hongtao Yu; UT Southwestern, Dallas, Texas, USA); and rabbit‐anti‐CNX (1:2,000; ab22595, abcam). *Secondary antibodies*: HRP‐linked anti‐mouse IgG (1:5,000; 7076S, Cell Signaling); HRP‐linked anti‐rabbit IgG (1:5,000; 7074S, Cell Signaling); and IRDye 800CW goat‐anti‐Rabbit IgG (1:5,000; 925‐32211, Li‐Cor).

### Tissue microscopy

Male C57BL/6 mice (10 weeks of age) were killed by decapitation under deep anesthesia with isoflurane. Brains were excised, covered with Tissue‐Plus O.C.T compound (4583, Scigen), snap frozen in liquid N_2_, and stored at −80°C. Cryosections (10 μm), covering the dorsal raphe nuclei, were prepared on a cryostat, immediately subjected to immunostaining or frozen at −80°C. Sliced tissue was fixed in acetone/methanol (1:1) for 15 min at −20°C, and embedding matrix was removed in ddH_2_O. Slides were attached to Shandon cover plates (Thermo Scientific) and inserted into a Shandon Sequenza holder. After washing in TBS (20 mM Tris–HCl, pH = 7.6; 150 mM NaCl), tissue was blocked in 5% normal goat serum + 5% normal donkey serum for 1 h at room temperature. Subsequently, tissue was incubated with indicated primary antibodies (as outlined under “Antibodies”) at 4°C overnight. After three wash steps in TBS, sections were incubated in fluorescent secondary antibodies for 1 h at room temperature, together with Hoechst 33342 (14533, Sigma Aldrich) at 1:3,000 for nuclear staining. All blocking and antibody dilutions were prepared in Dako antibody diluent (Agilent). After three wash steps in TBS, slides were mounted in Fluoromount‐G Mounting Medium (00‐4958‐02, Invitrogen) and covered with glass coverslips. Fluoromount‐G solidified overnight under brass weights (~100 g per slide).

Alternatively, tissue sections were stained with hematoxylin/eosin (H&E) using a standard protocol (publicly available from the Adler lab, Johns Hopkins University).

Mounted tissue sections were imaged on an Olympus AX70 epifluorescence microscope using a magnification of 10‐ and 20‐fold for H&E stainings and for immunofluorescence, respectively. Adjacent single images were acquired in order to afford multiple image alignments (MIA) arranged by Olympus Cell^P software. In addition, identical slides were imaged on a Zeiss LSM 510 laser scanning confocal microscope equipped with Plan‐Apochromat 63×/1.4 oil‐immersion objective. Either single optical planes were acquired or Z‐stacks with a slice thickness of 0.4 μm. Images were analyzed using ImageJ/Plot Profile software.

### Immunoblotting of mouse brain proteins

Male C57BL/6 mice (23 weeks of age) were killed by decapitation under deep anesthesia with isoflurane and their brains were excised. For immunoblots of midbrain proteins, the respective area was separated from the residual brain. Tissues were homogenized in 2 ml co‐IP lysis buffer (20 mM Tris–HCl, pH = 7.6; 150 mM NaCl; 1 mM EDTA; 10% glycerol; 1% Nonidet P40 Substitute; protease inhibitor cocktail [Roche]; PhosSTOP [Roche]) using a Dounce homogenizer. Lysates were cleared at 16,100 *g* for 30 min at 4°C. Protein concentrations were measured by dye binding (Coomassie Brilliant Blue G‐250) and diluted to 1 mg ml^−1^ in co‐IP lysis buffer. Samples (20 μg) were analyzed by standard SDS–PAGE and immunoblotting.

### GST Protein purification and GST pull‐down

GST proteins were purified from transformed XL10‐Gold *Escherichia Coli* as described previously (El‐Kasaby *et al*, 2014). For GST protein‐based interaction assays, 25 μg GST proteins were prebound to glutathione Sepharose (20 μl packed resin per sample) in 200 μl TBS‐T for 2 h at room temperature under end‐over‐end rotation. Meanwhile, adherent HEK‐293 cells on confluent 10 cm dishes (approx. 9 × 10^6^ cells per dish) were lysed in 450 μl ice‐cold co‐IP lysis buffer. Lysates were cleared at 16,100 *g* at 4°C for 30 min. The supernatant was recovered and used for GST pull‐down assays.

Immobilized GST‐proteins were washed once in 500 μl co‐IP lysis buffer and incubated with cleared HEK‐293 lysate (150 μl ≙ ~3 × 10^6^ cells per sample) for 1 h at room temperature. Associated proteins were washed three times in 500 μl ice‐cold TBS‐T and eluted in 50 μl conventional 2× Laemmli SDS–PAGE sample buffer at 90°C for 10 min. In the same step, 50 μl of a 1:10 dilution of the cleared lysate was denatured by adding 50 μl 2× sample buffer. Samples were analyzed by standard immunoblotting or frozen at −80°C.

### Plasmids, molecular cloning, and mutagenesis

For YFP‐hMAD2, 600 ng total RNA of human primary mesenchymal stem cells were reversely transcribed using the RevertAid RT Reverse Transcription Kit (K1691, Thermo Scientific) according to the manufacturer. Subsequently, the sequence encoding for human MAD2 was amplified by PCR using primers flanked by restriction sites for BamHI and HindIII (BamHI_MAD2_rv: 5′‐GTACGTGGATCCTCAGTCATTGACAGGAATTTTGTAGG‐3′; HindIII_MAD2_fw: 5′‐GTACGTAAGCTTATGGCGCTGCAGCTCTCCC‐3′). PCR product (2 μg) and pEYFP‐C1 vector DNA (2 μg) were digested with BamHI‐HF and HindIII‐HF (New England Biolabs) according to the manufacturer. Digested DNA was cleared from restriction enzymes using the NucleoSpin Gel and PCR Clean‐up kit (740609.250, Macherey‐Nagel). Insert DNA (50 ng) was ligated with vector DNA (150 ng) using the Fast‐Link DNA Ligation Kit (LK0750H, Lucigen). XL10‐Gold ultracompetent cells were transformed with the ligation product and streaked out on agar plates containing Kanamycin. Purified plasmid from resulting colonies was analyzed by control digestions with BamHI and HindIII and positive clones were confirmed by sequencing (LGC Genomics).

For YFP‐hGAT1, DNA was amplified by PCR using primers flanked by restriction sites for HindIII and KpnI (HindIII_hGAT1_fw: 5′‐GACTGTAAGCTTTGGCGACCAACGGCAGCAAGGT‐3′; KpnI_hGA T1_rv: 5′‐GACTGTGGTACCCTAGATGTAGGCCTCCTTGCTGGTGG‐3′). Subsequent steps were identical to YFP‐hMAD2 cloning (see above) with KpnI‐HF (New England Biolabs) replacing BamHI‐HF. Digested PCR product (150 ng) was ligated with digested pEYFP‐C1 (150 ng).

Mutations were introduced by use of the QuikChange Lightning Site‐Directed Mutagenesis Kit (Agilent Technologies) according to the manufacturer using the Agilent QuikChange Primer Design tool for primer design.

Generation of lentiviral plasmids is outlined under “Materials and Methods; Lentivirus production.”

### Co‐immunoprecipitation

HEK‐293 cells stably expressing indicated YFP‐tagged transporters were grown in 15 cm dishes to ~80% confluency. Dishes were placed on ice and washed once in ice‐cold TBS. Ice‐cold co‐IP lysis buffer (500 μl) was applied, material scraped off the dish, and transferred into 1.5 ml Eppendorf tubes. Cell lysis was completed at 4°C under end‐over‐end rotation for 30 min. Lysates were cleared at 16,100 *g* at 4°C for 30 min. Meanwhile, GFP‐Trap agarose (gta‐100, Chromotek) (~12.5 μl packed resin per sample) was equilibrated in co‐IP lysis buffer. Cleared lysates were incubated with GFP‐Trap agarose for 90 min at 4°C under end‐over‐end rotation. Agarose was washed thrice in 500 μl co‐IP lysis buffer. Associated proteins were eluted in 50 μl 2× Laemmli SDS‐PAGE sample buffer for 15 min at 60°C. In the same step, 50 μl of a 1:10 dilution of the cleared lysate was denatured by adding 50 μl 2× sample buffer. Samples were analyzed by western blotting or frozen at −80°C.

For co‐IPs after siRNA‐mediated MAD2‐knockdown, 3 × 10^6^ HEK‐293 cells stably expressing YFP‐SERT in 10 cm dishes were transfected with siRNAs as outlined below. Two days after transfection, cells were used for co‐IP experiments as described above.

### siRNA transfection

HEK‐293 cells in different culture formats were transfected with three target‐specific siRNAs against human MAD2 (sc‐35837, Santa Cruz Biotechnology) or with siRNA against luciferase (Dharmacon) as a control. Lipofectamine RNAiMax transfection reagent (13778150, Invitrogen) was used for complex formation at a ratio of 3 pmol siRNA/μl. The final siRNA concentration in the culture medium was 10 nM. Gene silencing was allowed to proceed for 48 h.

### Cell culture confocal microscopy

HEK‐293 cells (1.25 × 10^4^/well) stably expressing YFP‐hSERT were seeded into eight‐well μ‐Slides (80826, ibidi). On the following day, cells were transfected with siRNA as outlined above. Two days after siRNA transfection, cells were imaged on a Zeiss LSM 510 laser scanning confocal microscope equipped with a 63× oil immersion objective, as described previously (Koban *et al*, 2015). The relative intracellular YFP‐SERT signal (RIS) was analyzed using ImageJ/polygonal selection software and calculated as:

$$\mathrm{RIS}=\log10\;\frac{\text{intracellular}\;\mathrm{YFP}\;\text{intensity}}{\text{area}}$$

One day after siRNA transfection, HEK‐293 cells stably expressing YFP‐hSERT were transfected with plasmids encoding mCh‐Rab5 and mCh‐Rab7A (25 ng per well) using 0.2 μl Lipofectamine 2000 transfection reagent (11668019, Invitrogen). Cells were imaged on the following day. Co‐localization was quantified using the JACoP plugin for ImageJ (Bolte & Cordelieres, 2006) for calculating the Manders' co‐localization coefficient (Manders *et al*, 1993) corresponding to the fraction of mCherry overlapping with YFP.

### Radioactive substrate uptake

YFP‐SERT‐expressing HEK‐293 cells (5 × 10^4^/well of a 48‐well plate) were transfected with siRNA as outlined above. Two days after transfection, cells were washed in Krebs‐HEPES buffer containing glucose (KHB; 10 mM HEPES, pH = 7.3; 120 mM NaCl; 3 mM KCl; 2 mM CaCl_2_; 2 mM MgCl_2_; 2 mM glucose monohydrate). Subsequently, cells were incubated for 1 min with [^3^H]5‐HT ranging from 0.2 to 30 μM (specific activity progressively diluted from 40 cpm fmol^−1^ to 267 cpm pmol^−1^). Non‐specific uptake was determined in the presence of 10 μM paroxetine. The cells were washed with 0.5 ml ice‐cold KHB and lysed in 1% SDS. The thus released radioactivity was measured by liquid scintillation counting. Specific [^3^H]5‐HT uptake was calculated as pmol/10^6^ cells/min. Cell number and MAD2 content were determined from wells seeded on the same plate by counting in a hemocytometer and by immunoblotting to verify the efficiency of siRNA‐mediated knockdown, respectively.

### Surface biotinylation

YFP‐SERT‐expressing HEK‐293 cells (6 × 10^5^ per 6 cm dish) were transfected with siRNA as outlined above. After 2 days, cells were washed in PBS containing 1 mM MgCl_2_ and 0.1 mM CaCl_2_ (PBS^2+^). Washed cells were incubated with PBS^2+^ containing 2 mg ml^−1^ Pierce Premium Grade Sulfo‐NHS‐SS‐Biotin (PG82077; Thermo Scientific) for 30 min on ice. The reaction was quenched twice for 15 min with 100 mM glycine in PBS^2+^ and cells were washed thrice in TBS. Cells were lysed in 0.3 ml RIPA‐buffer (20 mM Tris–HCl, pH = 7.6; 150 mM NaCl; 1 mM EDTA; 1% Triton X‐100; 0.1% SDS; 0.5% sodium deoxycholate; protease inhibitor cocktail; phosSTOP). Lysates were cleared at 16,000 *g* for 30 min at 4°C. High‐capacity streptavidin agarose (20357, Thermo Scientific) was equilibrated in RIPA buffer. An aliquot (30 μl) of the cleared lysate was saved; the remaining lysate was incubated with streptavidin agarose (~25 μl packed resin/sample) overnight at 4°C under end‐over‐end rotation. Agarose was washed thrice in 0.5 ml RIPA buffer lacking sodium deoxycholate and protein/phosphatase inhibitors. Immobilized biotinylated proteins were eluted in 0.1 ml 2× Laemmli SDS–PAGE sample buffer at 90°C for 10 min. Concomitantly, the aliquot of the lysate was denatured after addition of 30 μl 2× sample buffer. Samples were either analyzed directly by western blotting or stored at −80°C.

### Lentivirus production

Forward and reverse primers (5′‐phosphorylated) encoding short hairpin RNAs (shRNAs), either targeting rat MAD2L1 mRNA or a random sequence not present in rat (scramble), were purchased from Microsynth.

Scramble‐sh fw: 5′‐TGACGAACGCATAGACGCATGATTCAAGAGATCATGCGTCTATGCGTTCGTCTTTTTTC‐3′
Scramble‐sh rv: 5′‐TCGAGAAAAAAGACGAACGCATAGACGCATGATCTCTTGAATCATGCGTCTATGCGTTCGTCA‐3′
MAD2‐sh fw: 5′‐TGCTGGTTTATACTGACAAAGTTTCAAGAGAACTTTGTCAGTATAAACCAGCTTTTTTC‐3′
MAD2‐sh rv: 5′‐TCGAGAAAAAAGCTGGTTTATACTGACAAAGTTCTCTTGAAACTTTGTCAGTATAAACCAGCA‐3′

Notably, the final primer pairs contain a blunt end and an XhoI‐compatible cohesive end for downstream cloning into the pLL3.7 lentiviral transfer plasmid.

Forward and reverse primers (50 μM) were annealed in the presence of 20 mM Tris–HCl, pH = 7.7, 50 mM NaCl, and 1 mM EDTA by placing the reaction (50 μl) at 95°C for 2 min. Thereafter, the reaction was allowed to cool down within ~1 h (by switching off the heating).

Lentiviral vector (pLL3.7) was kindly provided by Dr. Yoav Ben Simon, Allen Institute, Seattle, WA, USA. Originally, pLL3.7 was a gift from Luk Parijs [Addgene plasmid # 11795; http://n2t.net/addgene:11795; RRID: Addgene_11795] (Rubinson *et al*, 2003) which was enzymatically digested with XhoI and HpaI (New England Biolabs) and dephosphorylated at the 5′‐ends using shrimp alkaline phosphatase (New England Biolabs) following the instructions by the manufacturer. This digestion opens the lentiviral vector just downstream of the mouse U6 promoter site, required for shRNA expression.

Subsequently, 12 fmol of digested and dephosphorylated pLL3.7 were ligated with 25 pmol annealed primer in a T4 DNA Ligase (M0202, New England Biolabs) reaction for 90 min at room temperature followed by inactivation at 65°C for 10 min. Ligation products were amplified in XL10‐Gold *E. coli* and confirmed by sequencing.

For virus production, shRNA‐encoding plasmids were transfected together with psPAX2 (a gift from Didier Trono [Addgene plasmid # 12260; http://n2t.net/addgene:12260; RRID:Addgene_12260]) and pMD2.G (a gift from Didier Trono [Addgene plasmid # 12259; http://n2t.net/addgene:12259; RRID:Addgene_12259]) into LentiX™ 293T cells (Allen Institute, Seattle, WA, USA; kindly provided by Dr. Yoav Ben Simon) by polyethylenimine (PEI) transfection. For a 10 cm dish, 8.6 μg psPAX2, 2.6 μg pMD2.G, and 8 μg shRNA‐encoding plasmid were mixed in 0.5 ml serum‐free DMEM and 60 μl PEI‐reagent (1 mg ml^−1^; sc‐360988A, Santa Cruz Biotechnology) was mixed with 440 μl serum‐free DMEM. Solutions were combined and complex formation allowed for 15 min. The whole reaction (1 ml) was dropped onto 80% confluent LentiX™ 293T cells growing in 10 ml DMEM/10% FBS/1 mM sodium pyruvate without antibiotics. On the following 2 days, medium was changed to 4 ml medium containing non‐essential amino acids (11140050, Gibco). Virus‐enriched supernatants (4 ml) from both days were harvested, pooled, and centrifuged at 55,000 *g*. The virus was resuspended in incomplete neuronal medium (neurobasal A, 1% heat‐inactivated calf serum, 0.4 mM glutamine, 50 μM kynurenic acid, w/o B27, w/o 5‐FDU, and w/o GDNF) to reach a 30‐fold higher concentration than in the original supernatants. Virus was aliquoted, frozen, and stored at −80°C.

The effect of the lentiviruses was verified by quantitative PCR (qPCR) and immunoblotting. In brief, rat glial cells were cultured for 2 weeks as described below and infected with scrambled and MAD2‐shRNA‐encoding lentiviral particles. After 5 days, cells were lysed to either extract RNA using an RNA extraction kit (EM09.2‐050, blirt; according to the manufacturer) or to prepare whole‐cell lysates in co‐IP lysis buffer for immunoblotting as described above. For qPCR, RNA was reversely transcribed using the RevertAid RT Reverse Transcription Kit (K1691, Thermo Scientific) according to the manufacturer. The resulting cDNA was used for quantification of rat MAD2L1‐ and rat βActin‐RNA using Maxima SYBR Green/ROX qPCR Master Mix (2×) (K0222, Thermo Scientific) and target‐specific qPCR primer pairs.

### Primary glial and neuronal culture

Cortical glial cell precultures were prepared from 1‐ to 3‐day‐old Sprague–Dawley rat pups. Rats were decapitated and their brains were placed immediately in ice‐cold PBS. The meninges were removed and two lateral pieces of the cortex were cut from the whole brain. Chopped tissue was digested in papain for 15 min at 37°C. Papain was removed by serial transfer into 3 × 3 ml of glial cell medium (DMEM/10% FCS + penicillin–streptomycin). Cells were singularized in 3 ml glial cell medium by resuspension with a regular pipette tip and by pipette tips pierced with a 23G or 25G hypodermic needle. Dissociated cells were pelleted for 5 min at 800 *g* and resuspended in medium. Cells were grown in 12‐well dishes containing sterile PDL‐ and laminin‐coated 15 mm glass coverslips. When cells reached 20% confluence (usually after 2 days), medium was changed to glial cell medium containing 5‐fluorodeoxyuridine (5‐FDU) in order to inhibit further cell proliferation. Such glial cultures were maintained for 1–4 weeks as feeder layer for primary neuronal cultures. On the day before neuronal culture, medium was exchanged to complete neuronal medium (neurobasal A, 2% B27, 1% heat‐inactivated calf serum, 0.4 mM glutamine, 50 μM kynurenic acid, 6.7 μg ml^−1^ 5‐FDU, and 10 ng ml^−1^ glia‐derived neurotrophic factor [GDNF]), resulting in glial‐conditioned neuronal medium the next day. On the day of neuronal culture, sterile glass cylinders (inner diameter ~6 mm) were placed in the middle of the 15 mm glass cylinders in order to limit the area of neuronal cell adhesion.

Dorsal raphe neurons were prepared from newborn Sprague–Dawley within 24 h after birth. Rats were decapitated and their brains were placed immediately in ice‐cold PUCK‐KYN buffer (137 mM NaCl, 5.4 mM KCl, 1.1 mM Na_2_HPO4 × 2 H_2_O, 1.1 mM KH_2_PO_4_, 6.1 mM glucose, and 1 mM kynurenic acid; pH = 7.3). The brain area containing the dorsal raphe nucleus was excised and stored in ice‐cold PUCK‐KYN, until the dissection of all brains was completed (≤ 30 min). Chopped dorsal raphe tissue was digested for 15 min at 37°C in papain, which was subsequently removed by serial transfer into 3 × 3 ml of fresh neuronal medium. Cells were singularized in 3 ml neuronal medium by resuspension as outlined above. Dissociated cells were pelleted for 5 min at 800 g resuspended in complete neuronal medium, counted, and diluted to 10^6^ cells ml^−1^. Cells (10^5^ per well) were dropped onto glial‐conditioned medium inside the glass cylinders, which were removed 2 h after seeding. Neurons were allowed to differentiate for 2 weeks before immunofluorescence or viral infection. For viral infection, concentrated virus (20 μl per well) was pipetted into the neuronal medium on top of the neurons. After 5 days, neurons were subjected to immunofluorescence staining.

### Immunofluorescence of dorsal raphe neurons

Uninfected or virus‐infected neurons on 15 mm coverslips were fixed in acetone/methanol at −20°C. After washing in PBS (3 × 5 min) on ice, coverslips were transferred onto parafilm inside a tissue culture dish. Non‐specific antibody binding sites were blocked in blocking/permeabilization buffer (PBS/0.1% saponin/5% donkey serum) for 1 h at room temperature. Antibody dilutions were prepared in antibody dilution buffer (PBS/0.1% saponin/1% BSA) at concentrations indicated in section “Antibodies” and cells were incubated at 4°C overnight. After washing in PBS (3 × 5 min), secondary antibodies were applied in antibody dilution buffer for 1 h at room temperature in the dark. Cells were washed in PBS (1 × 10 min, 3 × 5 min) with Hoechst 33342 (0.2 μg ml^−1^) present in the first wash step. Coverslips were mounted onto glass slides in 85% glycerol/15% PBS and sealed with nail polish. Images were captured on a Nikon A1 laser scanning microscope using a 20× air or 60× oil objective. Images were analyzed using ImageJ software.

### Statistical analysis

The statistical analysis was performed using GraphPad Prism software. *P*‐values below 0.05 were considered significant. The specific statistical tests as well as the exact *P*‐values are indicated in the figure legends. The number (*n*) of experiments in the figure legends indicates biological replicates.

## Author contributions

**Florian Koban:** Conceptualization; formal analysis; investigation; methodology; writing – original draft; writing – review and editing. **Michael Freissmuth:** Formal analysis; funding acquisition; writing – original draft; writing – review and editing.

## Disclosure and competing interests statement

The authors declare that they have no conflict of interest.

## Supporting information



Expanded View Figures PDF

PDF+

Source Data for Figure 1

Source Data for Figure 2

Source Data for Figure 3

Source Data for Figure 4

Source Data for Figure 5

## Data Availability

This study includes no data deposited in external repositories.
