# Supplementary material for: The cell cycle protein MAD2 facilitates endocytosis of the serotonin transporter in the neuronal soma
Source: EMBO Rep. 2023 Aug 2;24(10):e53408. doi: 10.15252/embr.202153408 (PMC10561363; doi:10.15252/embr.202153408)
Supplement: Supplementary file 1 — Expanded View Figures PDF [file EMBR-24-e53408-s007.pdf]

Expanded View Figures

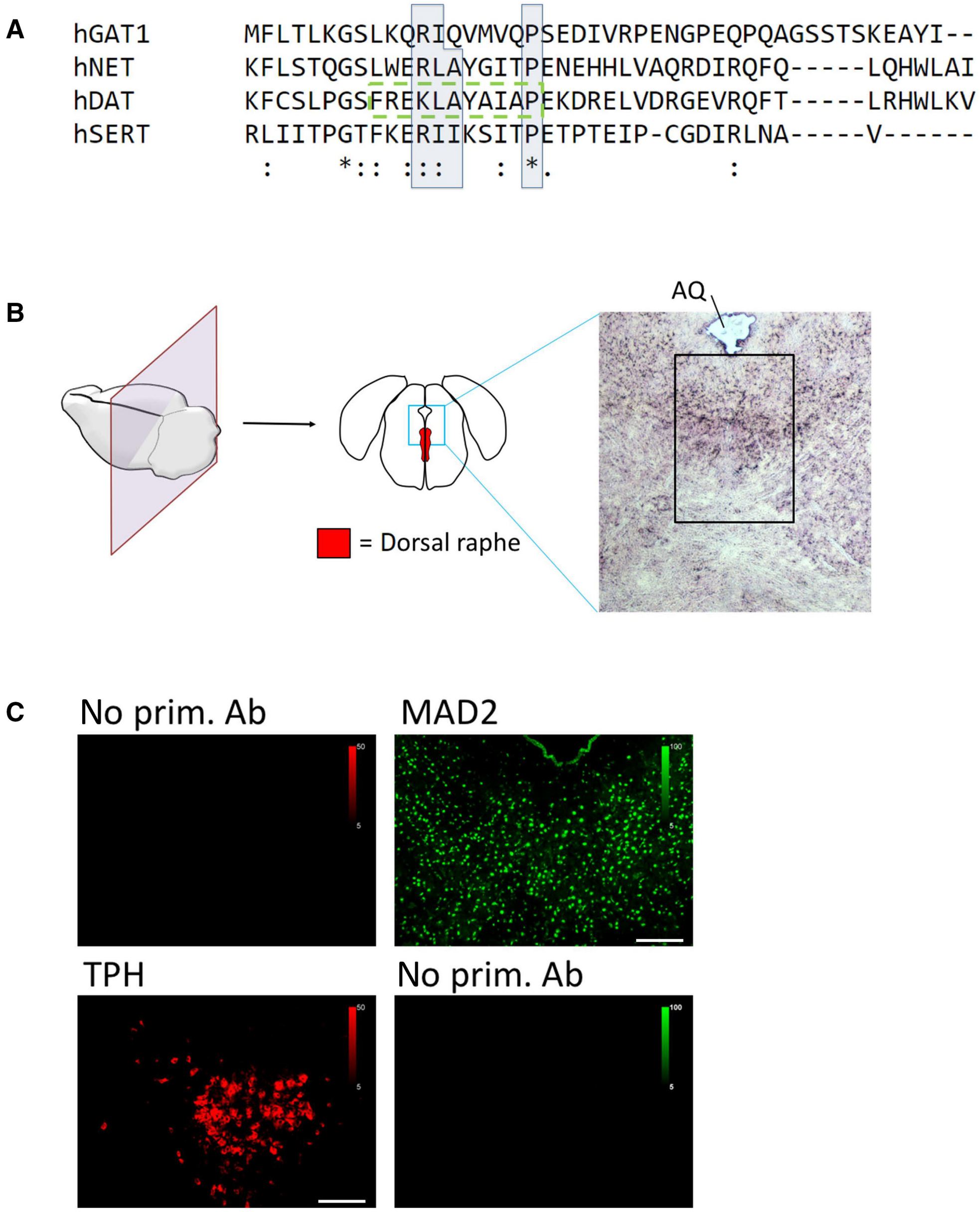

Figure EV1. Conservation of transporter C-terminal MIMs and control stainings for antibodies.

- A The C-termini of indicated transporters were aligned using Clustal Omega software. Conserved residues of candidate MIMs are highlighted by blue boxes; (*)—fully conserved residue; (:)—residues of strongly similar properties; (.)—residues of weakly similar properties. The previously described endocytic motif in DAT is highlighted by the green dashed box.
- B Cryosections of mouse brain were prepared covering the dorsal raphe and stained with hematoxylin/eosin. The black box indicates the approximate area of immunofluorescence images in Figs 1B and 3A and B. AQ = cerebral aqueduct.
- C Mouse brain sections were subjected to immunofluorescence microscopy as described under "Materials and Methods," with or without polyclonal antibodies for either TPH or MAD2, as indicated. Scale bars represent 100 µm.

© 2023 The Authors

Figure EV2. Association of full-length transporters with BubR1, β2-adaptin, and p31^{comet} correlates with MAD2 association.

A Densitometric signals in Fig 3C/co-IP:YFP were quantified using ImageJ and normalized to their respective transporter signal intensities; error bars represent SEM. Data derive from seven (MAD2), six (BubR1), seven (p31^{comet}), six (β2-adaptin), and six (HSP70) independent biological replicates.

B–E Scatter plots for the co-IP of indicated proteins (*y*-axis) against the co-IP of MAD2 (*x*-axis) (cf. Fig 3C); r^2 indicates the squared linear correlation coefficient; ***$P < 0.001$, ****$P < 0.0001$, ns—not significant. Data derive from six (BubR1), seven (p31^{comet}), six (β2-adaptin), and six (HSP70) independent biological replicates.

F, G HEK-293 cells were transfected with the indicated siRNAs and cell lysates were used for GST pull-down experiments using the indicated GST proteins. One-way ANOVA with Dunnett's multiple *post hoc* comparison; **$P < 0.01$, ***$P < 0.001$; error bars represent SD of three independent biological replicates.

 © 2023 The Authors

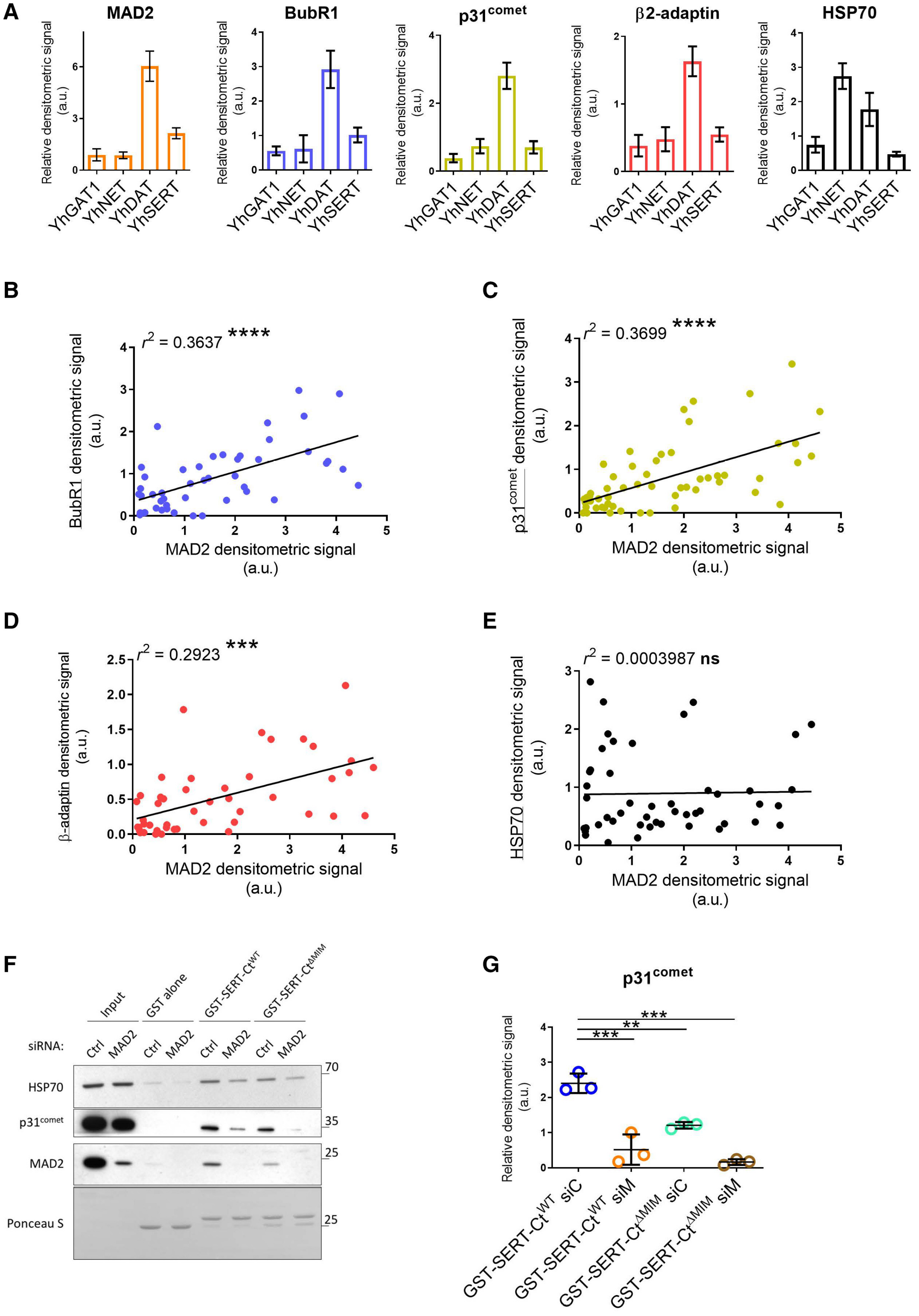
A
MAD2
BubR1
p31comet
β2-adaptin
HSP70
Relative densitometric signal (a.u.)
YhGAT1
YhNET
YhDAT
YhSERT
B
r2 = 0.3637 ****
BubR1 densitometric signal (a.u.)
MAD2 densitometric signal (a.u.)
C
r2 = 0.3699 ****
p31comet densitometric signal (a.u.)
MAD2 densitometric signal (a.u.)
D
r2 = 0.2923 ***
β-adaptin densitometric signal (a.u.)
MAD2 densitometric signal (a.u.)
E
r2 = 0.0003987 ns
HSP70 densitometric signal (a.u.)
MAD2 densitometric signal (a.u.)
F
Input
GST alone
GST-SERT-CtWT
GST-SERT-CtΔMIM
siRNA:
Ctrl
MAD2
HSP70
p31comet
MAD2
Ponceau S
70
35
25
25
G
p31comet
Relative densitometric signal (a.u.)
***
**
***
GST-SERT-CtWT siC
GST-SERT-CtWT siM
GST-SERT-CtΔMIM siC
GST-SERT-CtΔMIM siM

Figure EV2.

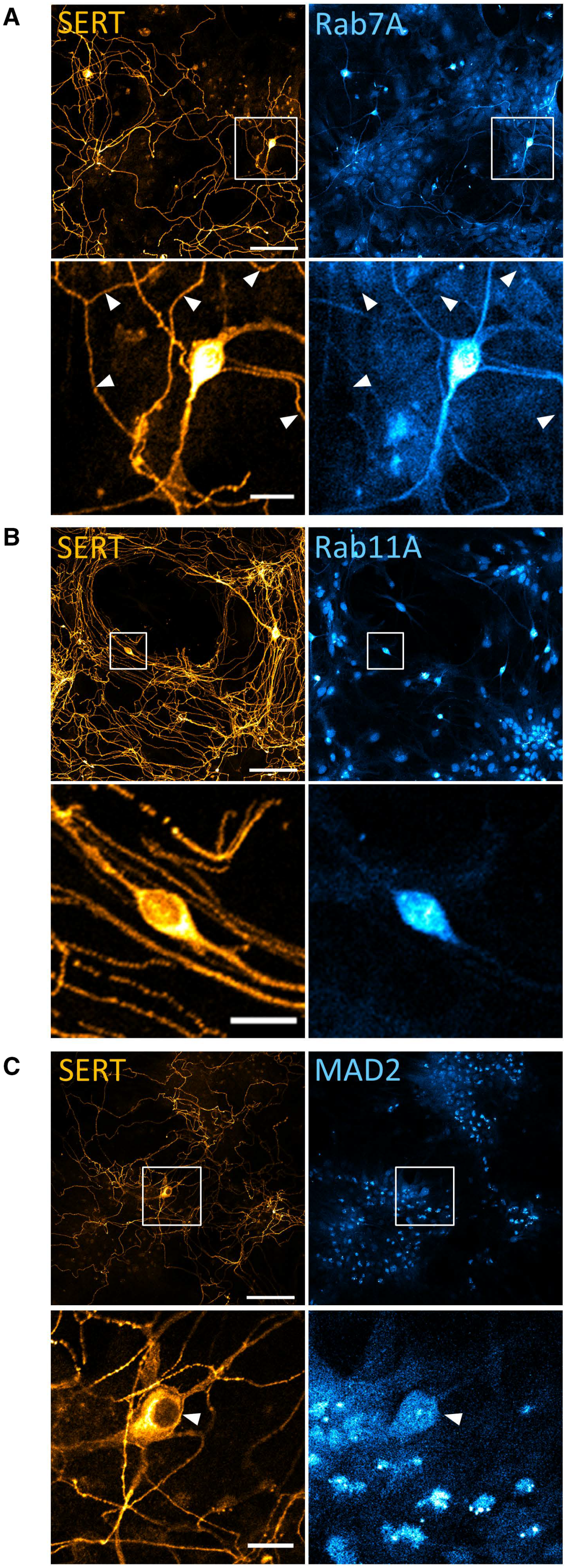

Figure EV3. Localization of Rab11A, Rab7A, and MAD2 in cultured rat dorsal raphe neurons.

A–C Primary rat dorsal raphe neurons were cultured as described under "Materials and Methods." After 14 days, cells were fixed in acetone/methanol (1:1) and subjected to immunofluorescence with the indicated antibodies. Confocal images were captured on a Nikon A1 laser scanning confocal microscope at 20× magnification. White boxes in the merged image indicate zoomed area in the lower panels. White arrowheads specify serotonergic extensions, as Rab7A was also detectable in non-serotonergic neurite extensions. Scale bars represent 100 and 20 μm for 20× image and zoomed area, respectively.

 © 2023 The Authors

Figure EV4. Co-localization between SERT and markers of the secretory pathway.

A–E Primary rat dorsal raphe neurons were cultured and fixed as for Fig EV3 and subjected to immunofluorescence with the indicated antibodies. Confocal images were captured on a Nikon A1 laser scanning confocal microscope at 60× magnification. White boxes in merged image indicate zoomed area in the lower left corner. Scale bars represent 20 μm in full-size images and 2 μm in zoomed areas.

© 2023 The Authors

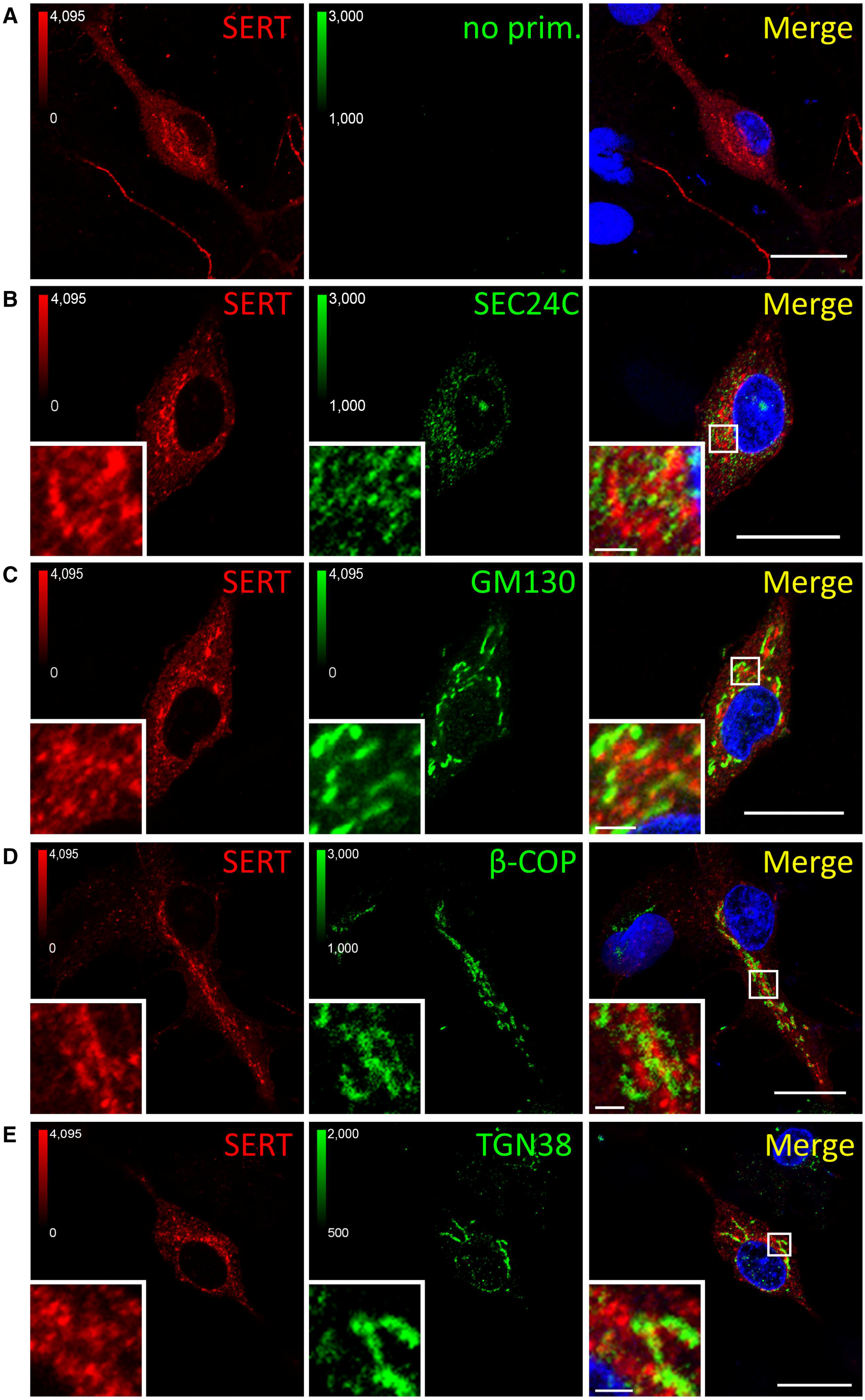

Figure EV4.

© 2023 The Authors

Figure EV5. Co-localization between SERT and endocytic markers.

A–E Primary rat dorsal raphe neurons were cultured and imaged as in Fig EV4, using the indicated primary antibodies. Scale bars represent 20 and 2 μm in full-size images and zoomed areas, respectively.

© 2023 The Authors

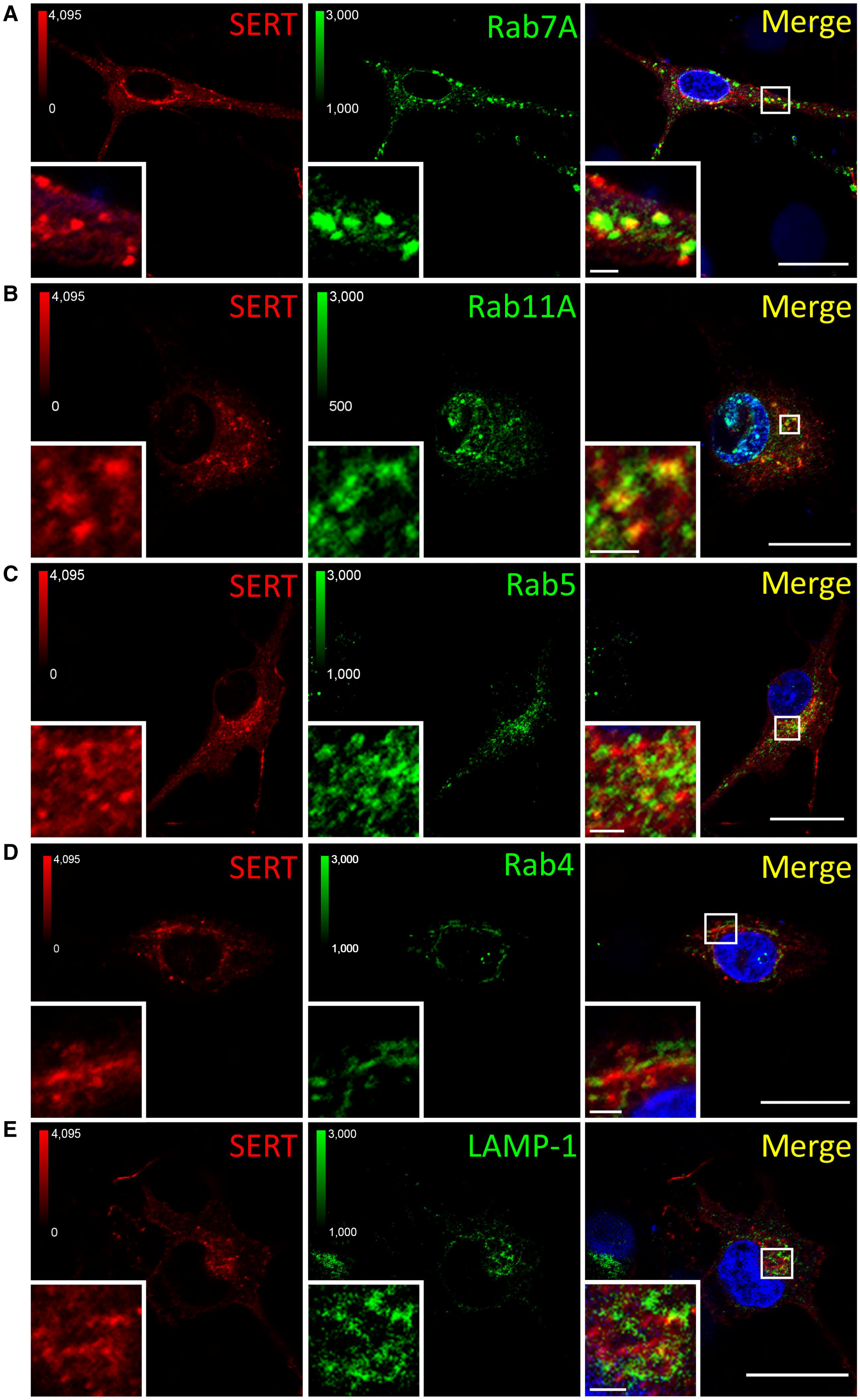

Figure EV5.

© 2023 The Authors
