# Supplementary figures and images for: The cell cycle protein MAD2 facilitates endocytosis of the serotonin transporter in the neuronal soma
Source: EMBO Rep. 2023 Aug 2;24(10):e53408. doi: 10.15252/embr.202153408 (PMC10561363; doi:10.15252/embr.202153408)

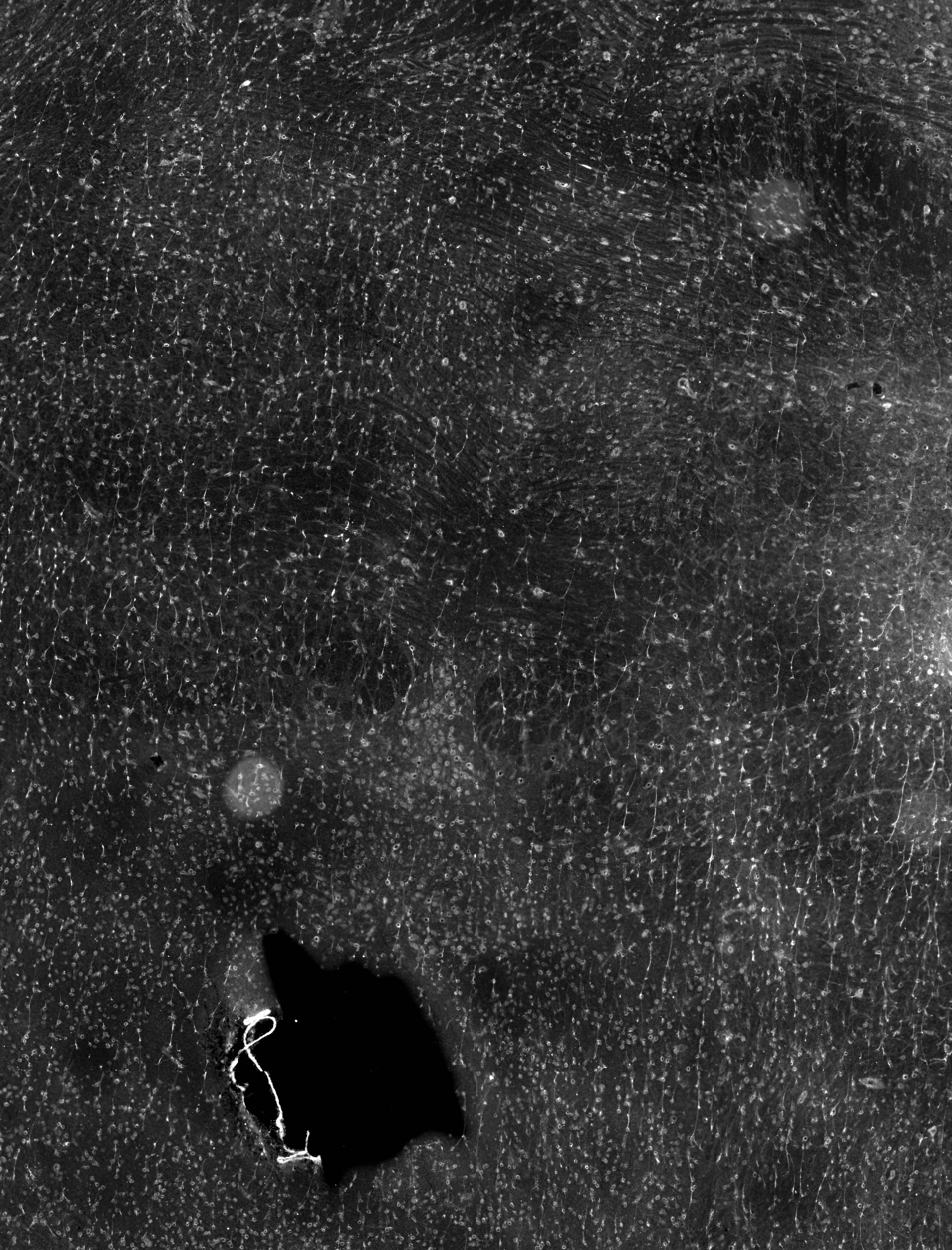

Supplement: Supplementary file 3 — Source Data for Figure 1 [file EMBR-24-e53408-s003.zip › Figure 1/1B/Fig 1B; MAD2 signal, original orientation.jpg]

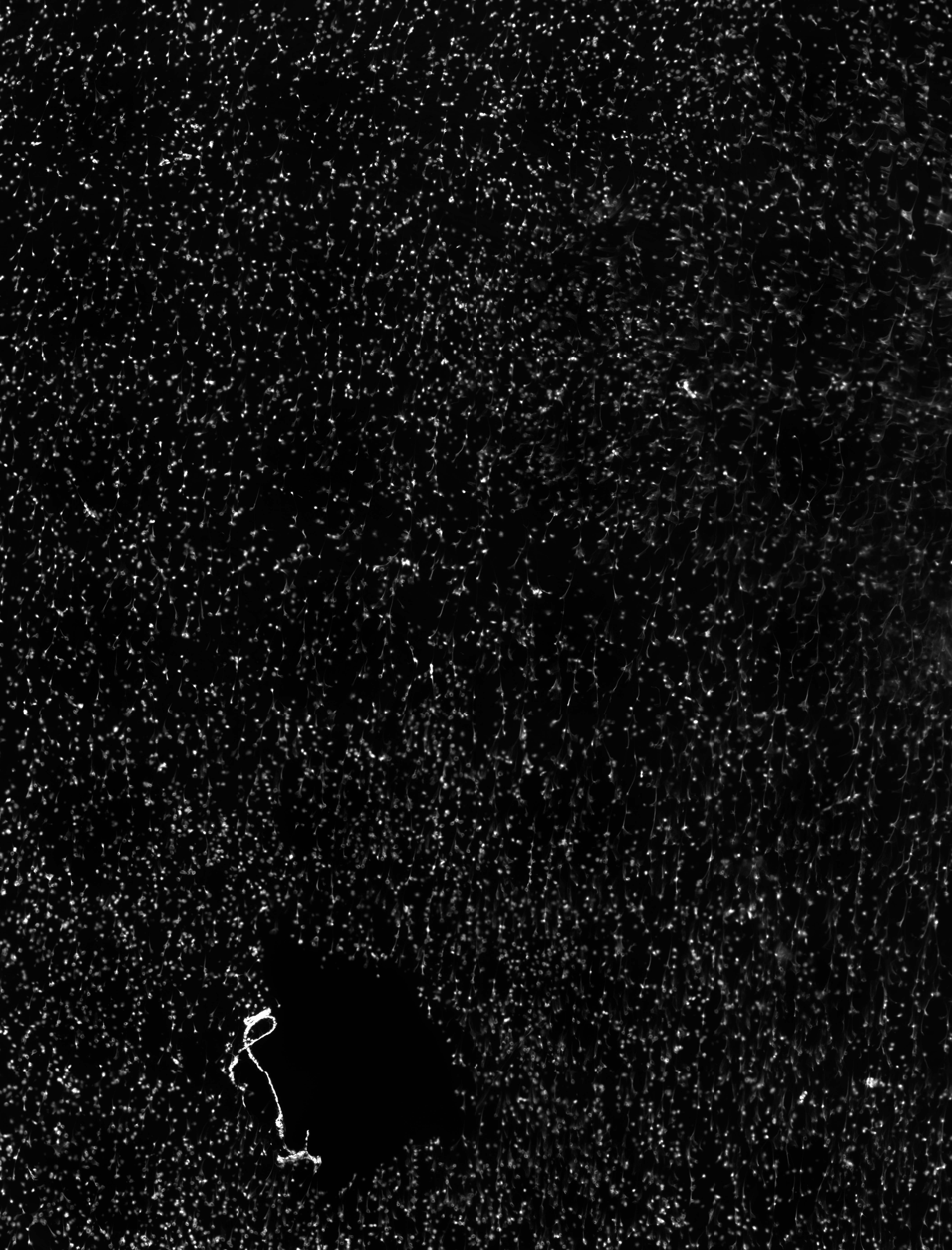

Supplement: Supplementary file 3 — Source Data for Figure 1 [file EMBR-24-e53408-s003.zip › Figure 1/1B/Fig 1B; Hoechst signal, original orientation.jpg]

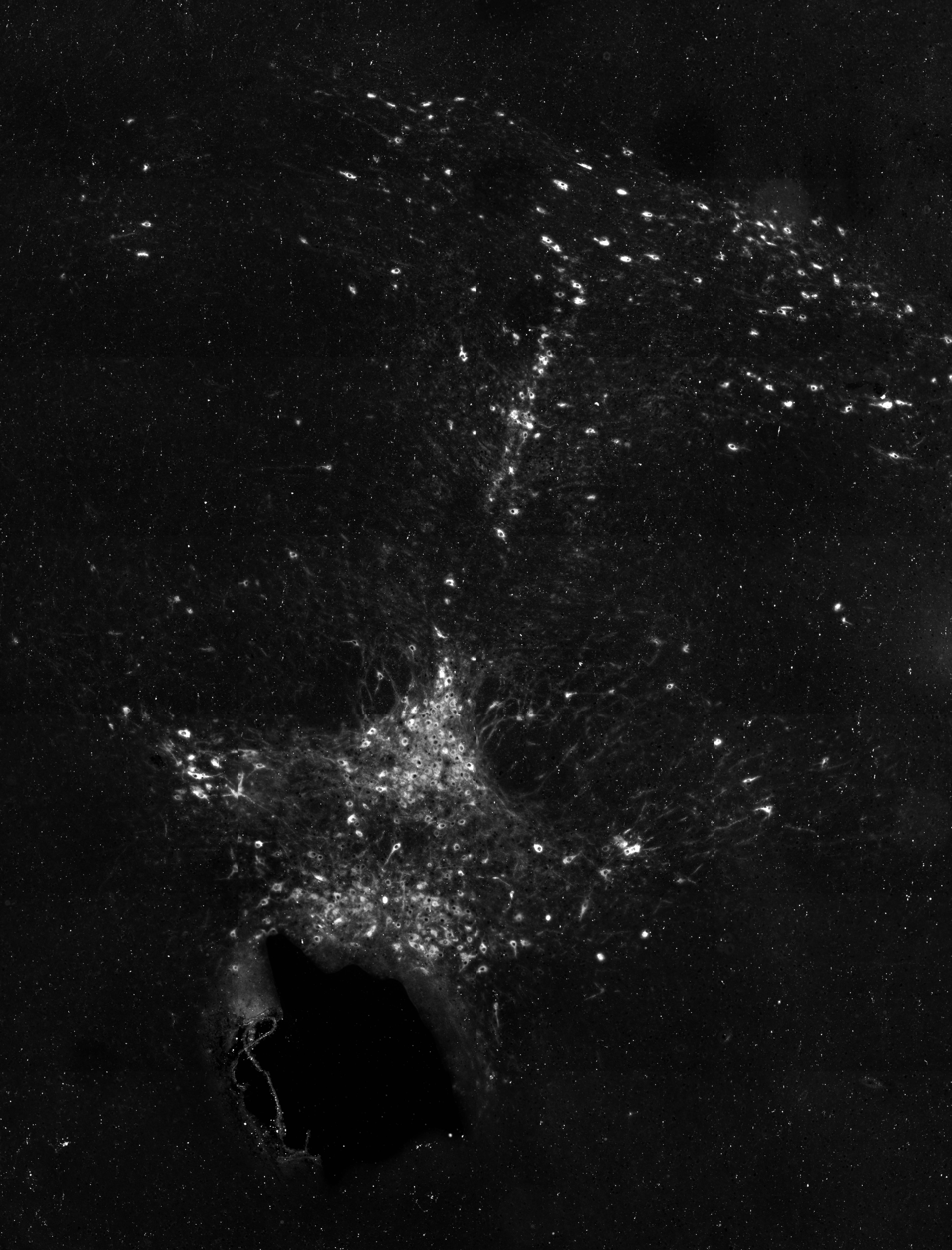

Supplement: Supplementary file 3 — Source Data for Figure 1 [file EMBR-24-e53408-s003.zip › Figure 1/1B/Fig 1B; TPH signal, original orientation.jpg]

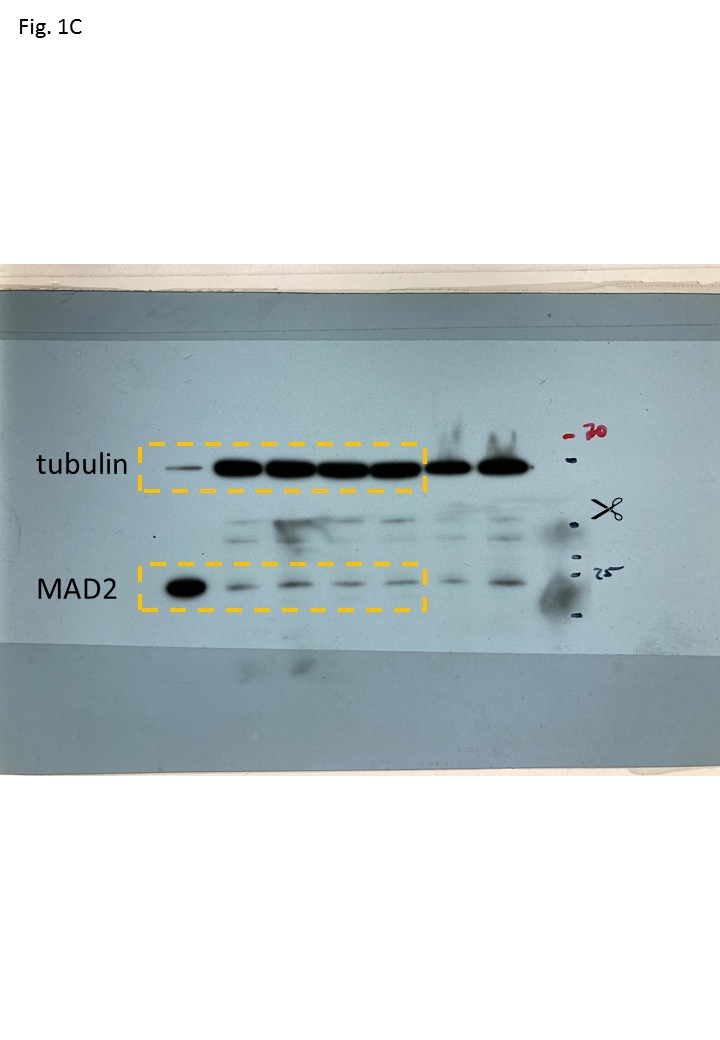

Supplement: Supplementary file 3 — Source Data for Figure 1 [file EMBR-24-e53408-s003.zip › Figure 1/1C/Immunoblot MAD2 + tubulin in mouse brain.tif]

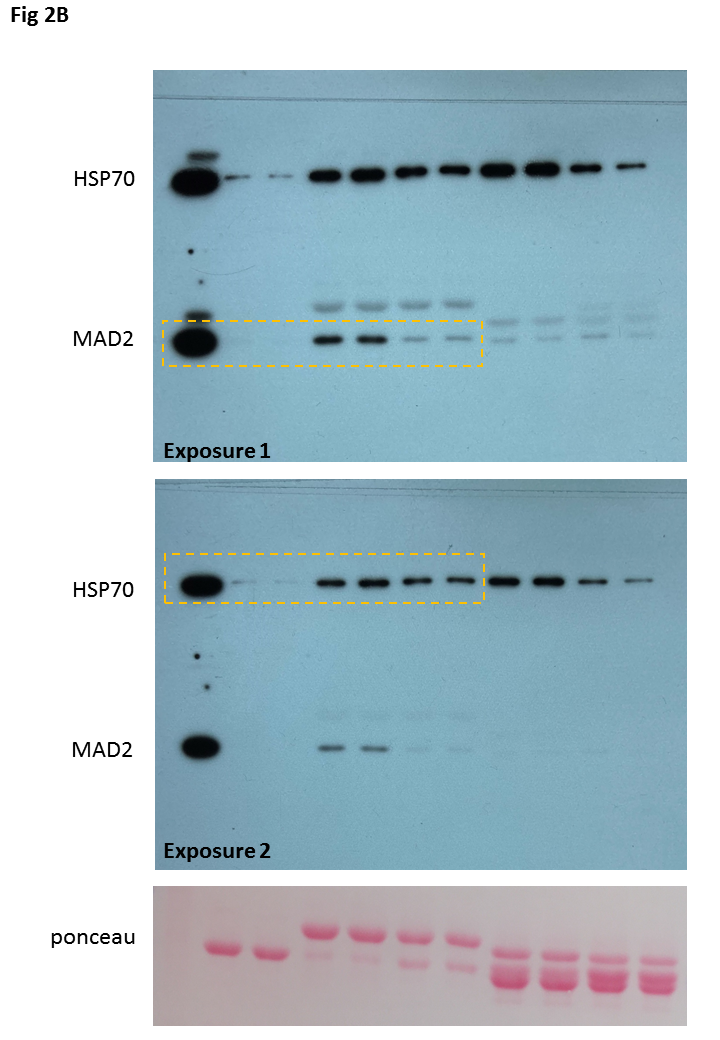

Supplement: Supplementary file 4 — Source Data for Figure 2 [file EMBR-24-e53408-s008.zip › Figure 2/2B/Immunoblot MAD2 + HSP70 + ponceau.TIF]

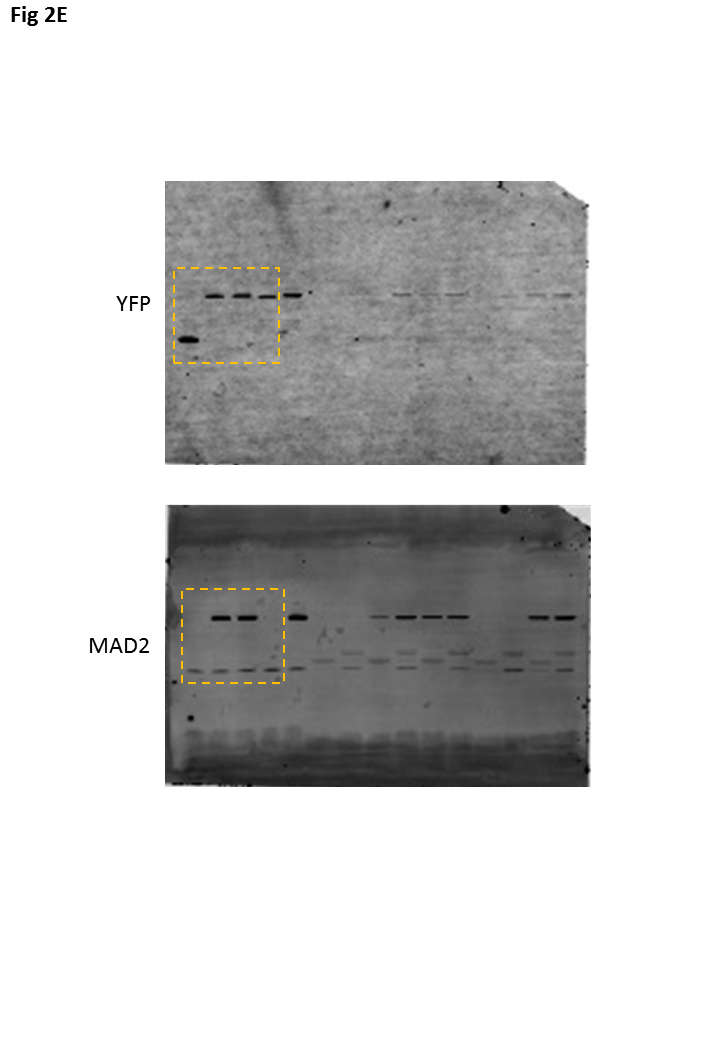

Supplement: Supplementary file 4 — Source Data for Figure 2 [file EMBR-24-e53408-s008.zip › Figure 2/2E/Immunoblot anti-YFP and anti-MAD2.TIF]

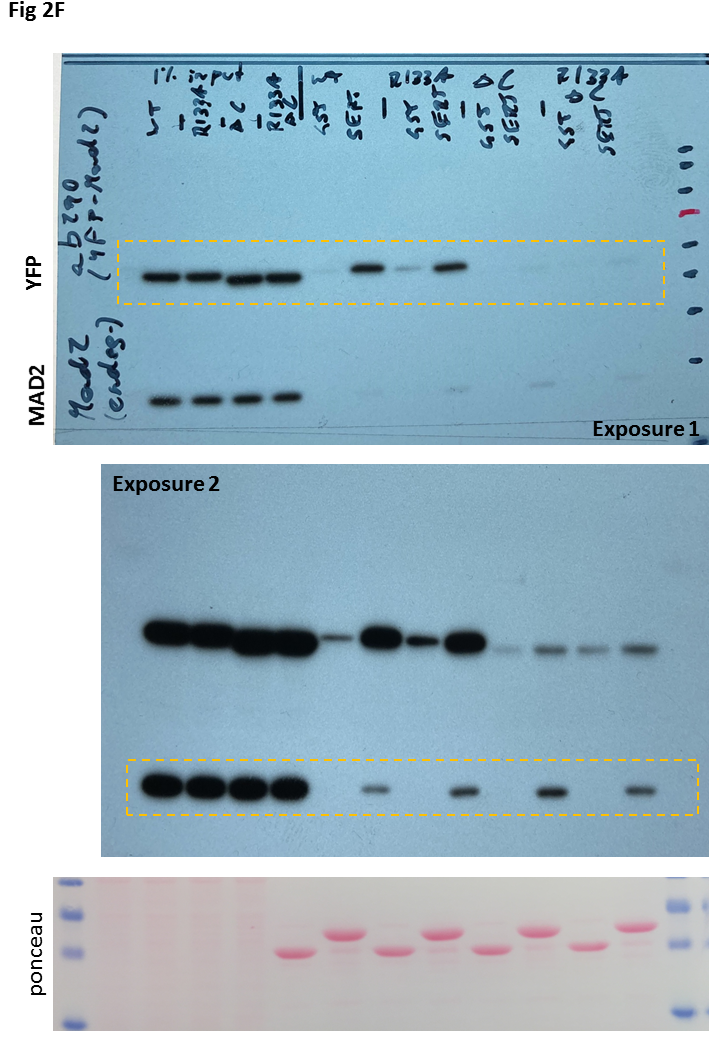

Supplement: Supplementary file 4 — Source Data for Figure 2 [file EMBR-24-e53408-s008.zip › Figure 2/2F/Immunoblot anti-MAD2, anti-YFP, ponceau.TIF]

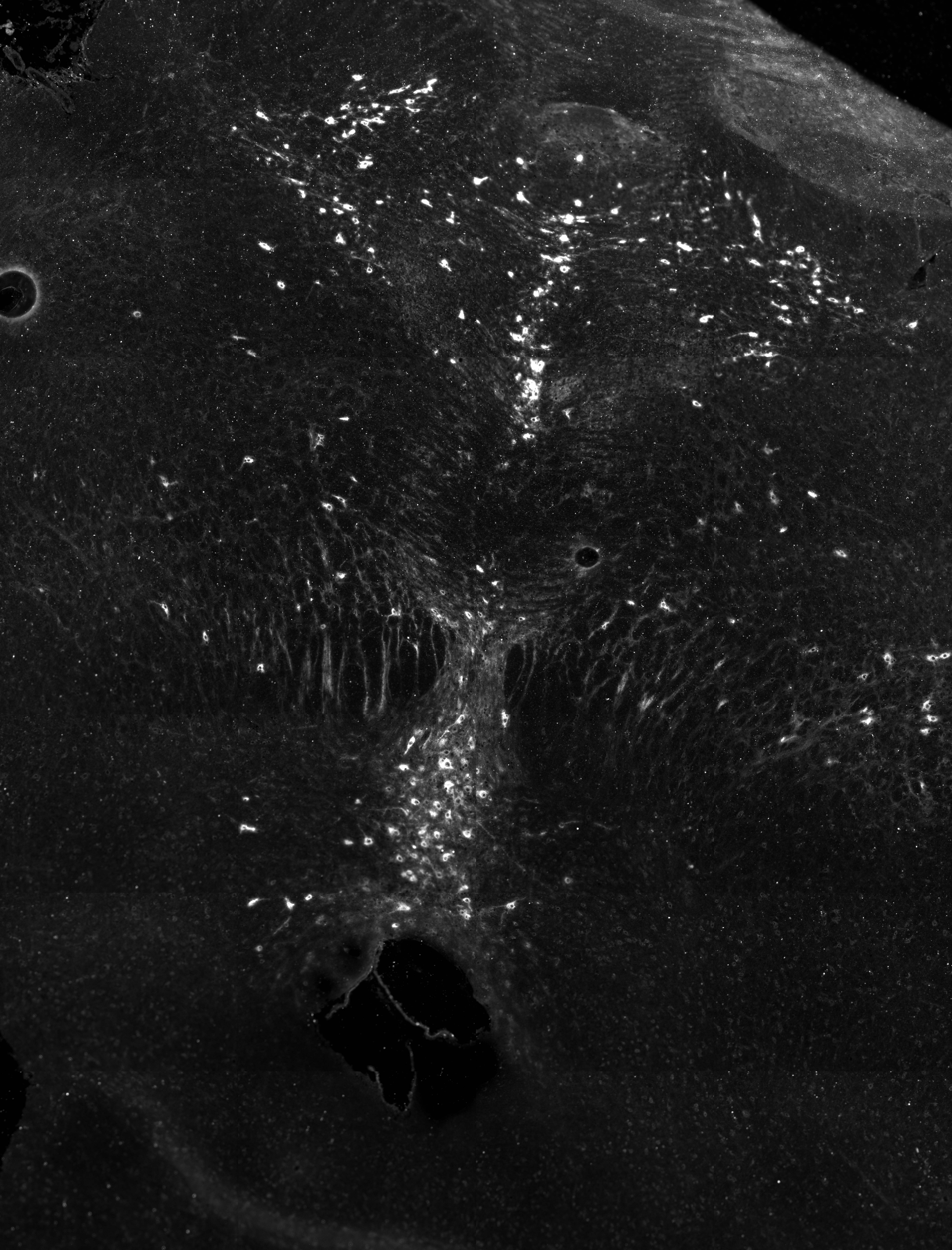

Supplement: Supplementary file 5 — Source Data for Figure 3 [file EMBR-24-e53408-s005.zip › Figure 3/3B/Fig 3B; TPH signal, original orientation.jpg]

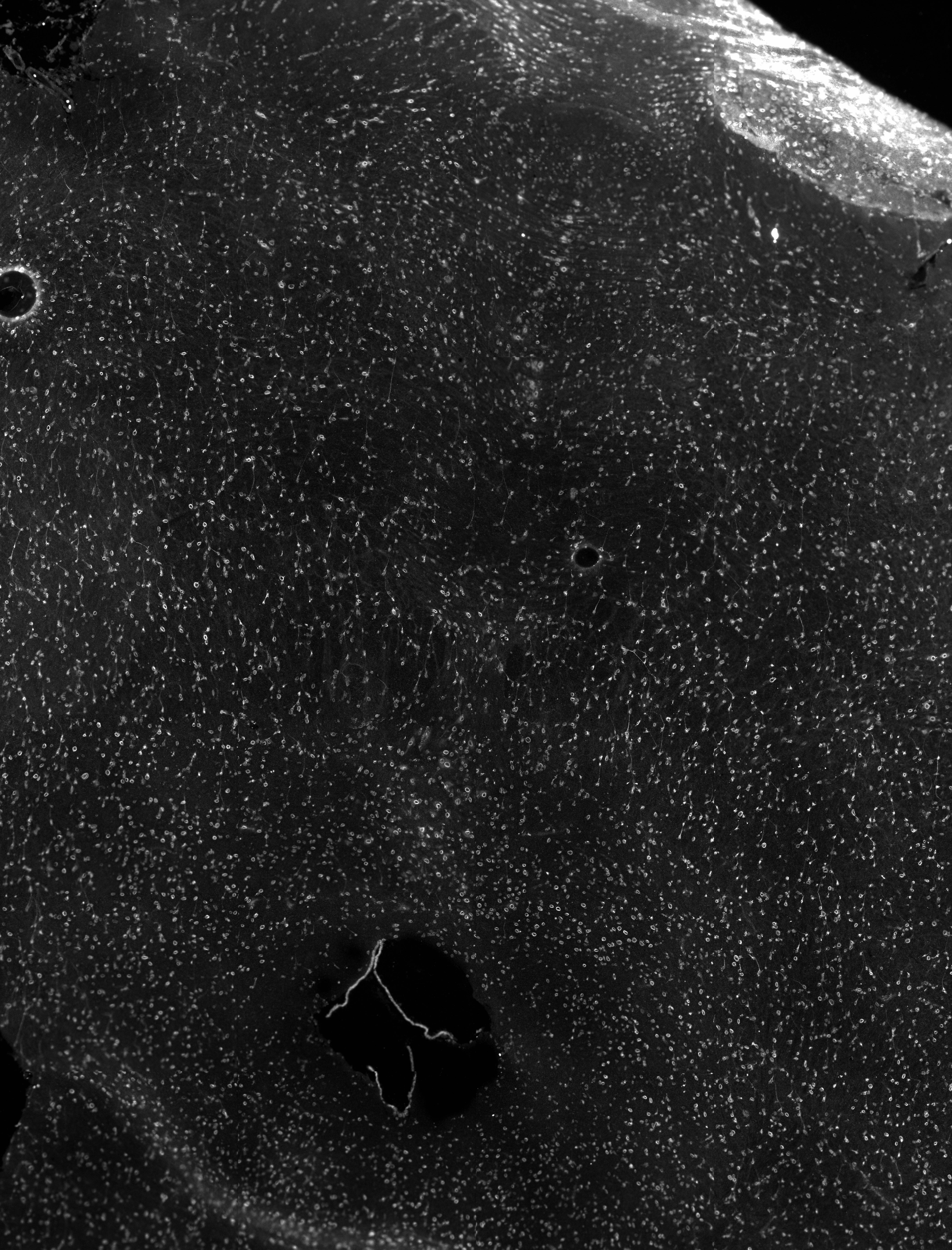

Supplement: Supplementary file 5 — Source Data for Figure 3 [file EMBR-24-e53408-s005.zip › Figure 3/3B/Fig 3B; p31comet signal, original orientation.jpg]

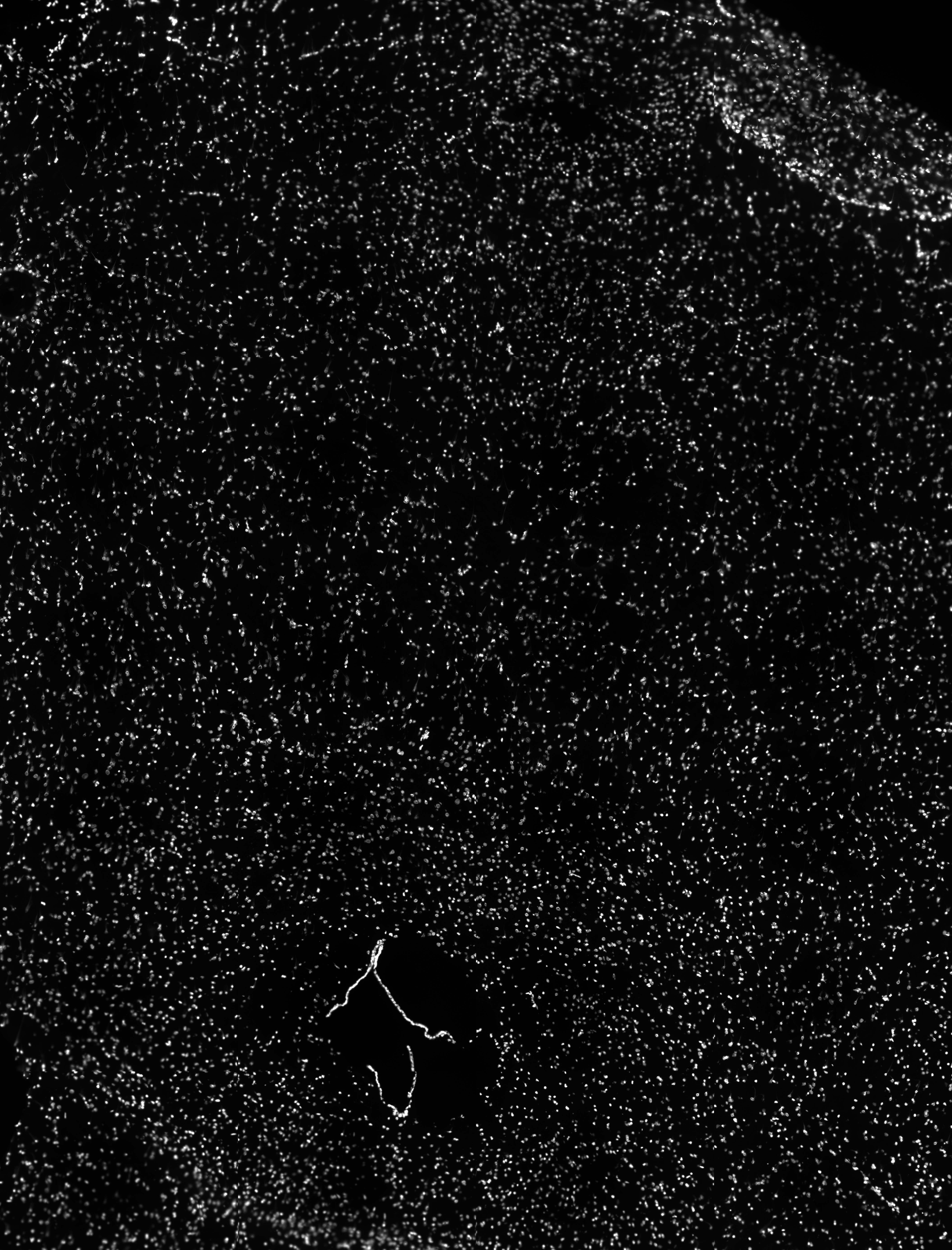

Supplement: Supplementary file 5 — Source Data for Figure 3 [file EMBR-24-e53408-s005.zip › Figure 3/3B/Fig 3B; Hoechst signal, original orientation.jpg]

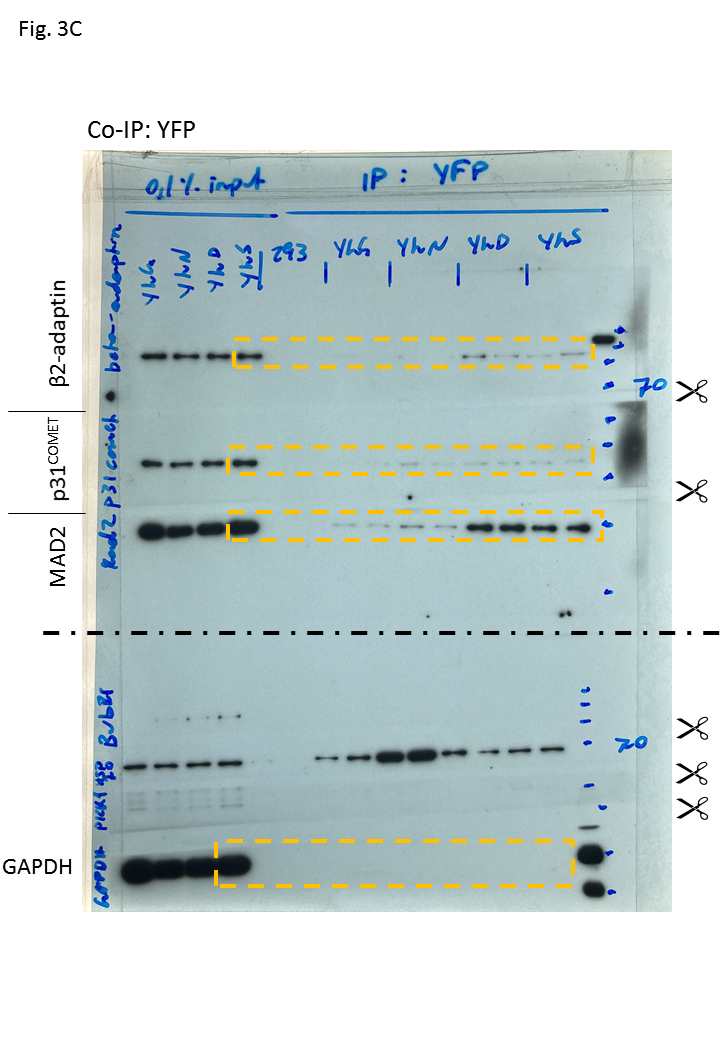

Supplement: Supplementary file 5 — Source Data for Figure 3 [file EMBR-24-e53408-s005.zip › Figure 3/3C/Immunoblot coIP-YFP for beta2-adaptin, p31comet, MAD2 and GAPDH (2).TIF]

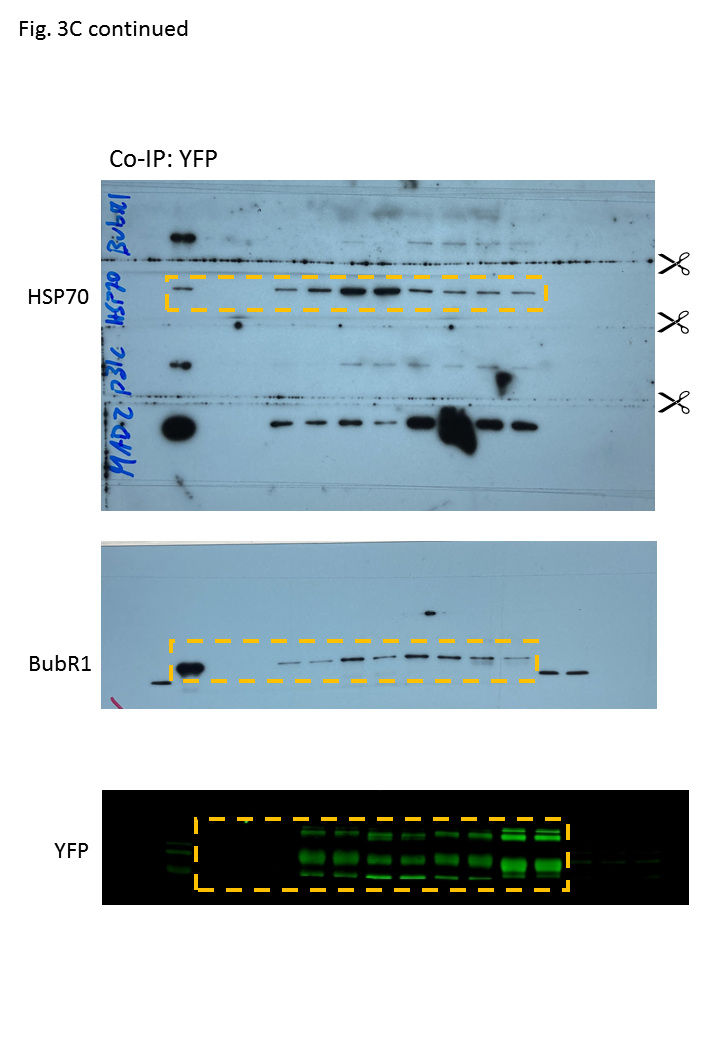

Supplement: Supplementary file 5 — Source Data for Figure 3 [file EMBR-24-e53408-s005.zip › Figure 3/3C/Immunoblot coIP-YFP for HSP70, BubR1 and YFP-SERT (2).TIF]

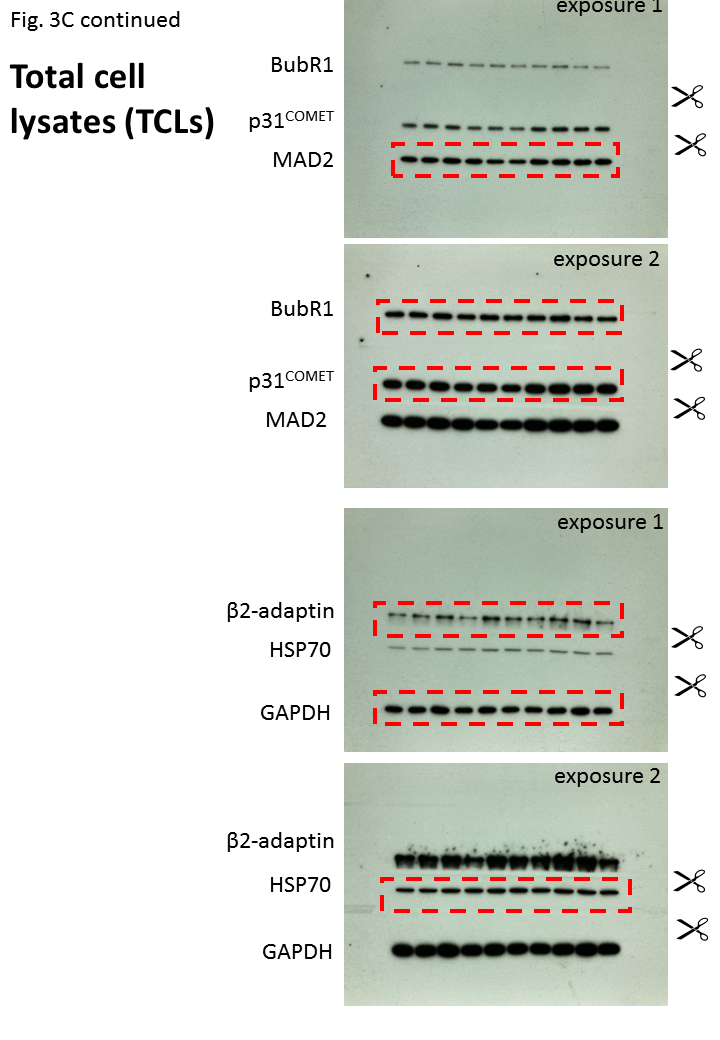

Supplement: Supplementary file 5 — Source Data for Figure 3 [file EMBR-24-e53408-s005.zip › Figure 3/3C/Immunoblot total cell lysates for all target proteins.TIF]

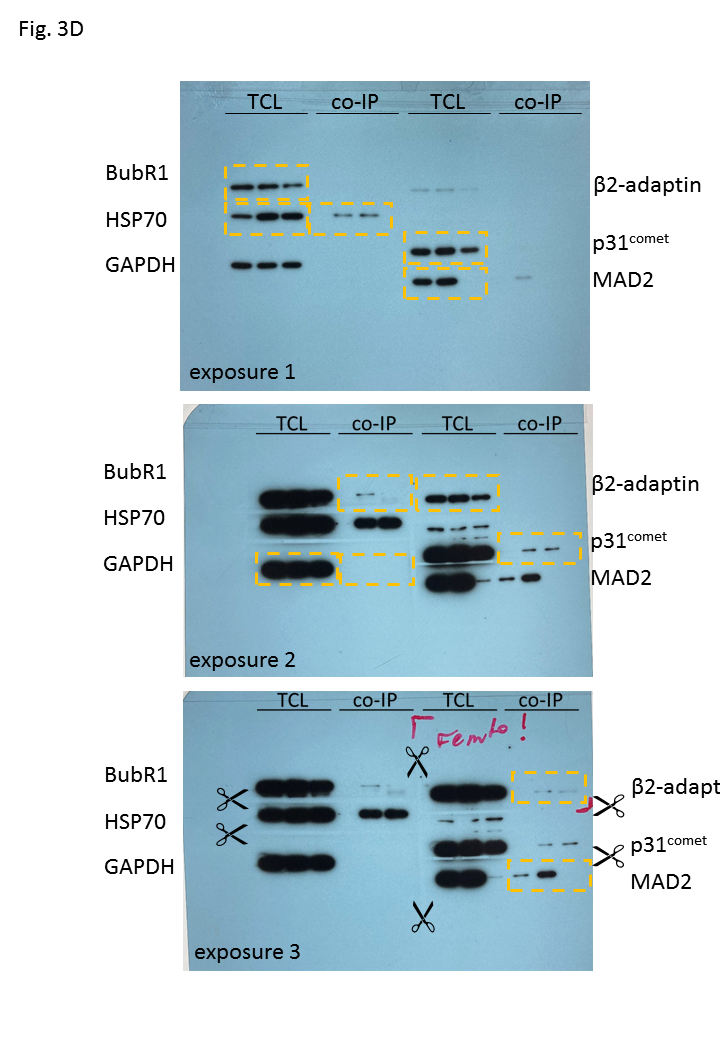

Supplement: Supplementary file 5 — Source Data for Figure 3 [file EMBR-24-e53408-s005.zip › Figure 3/3D/Immunoblot for all target proteins.TIF]

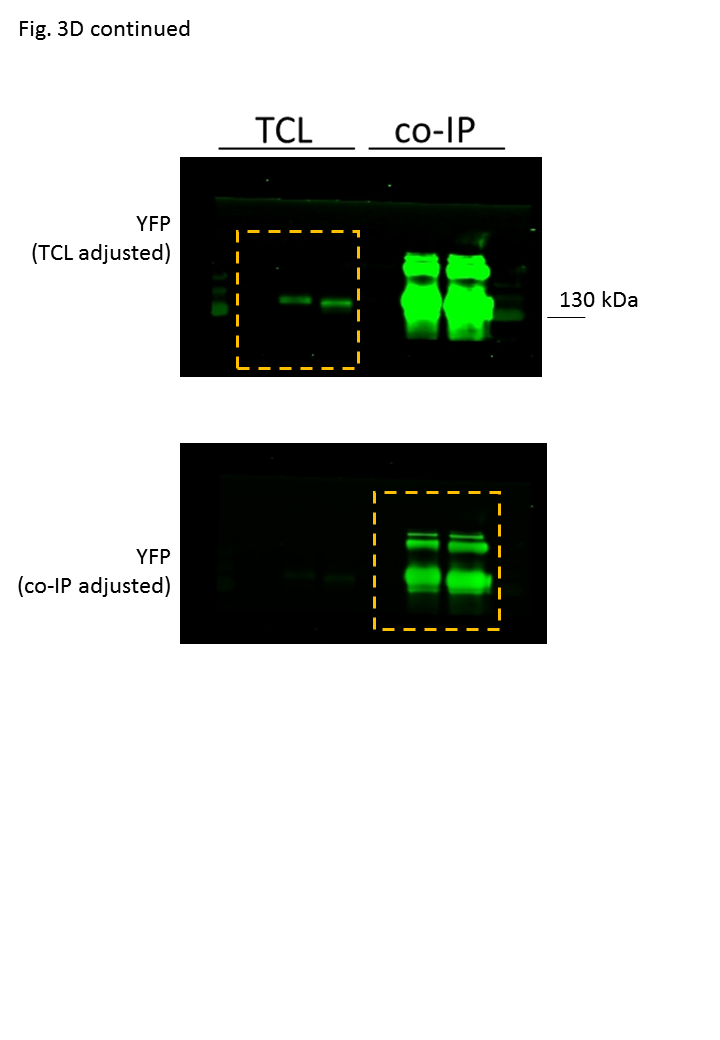

Supplement: Supplementary file 5 — Source Data for Figure 3 [file EMBR-24-e53408-s005.zip › Figure 3/3D/Immunoblot YFP-SERT.TIF]

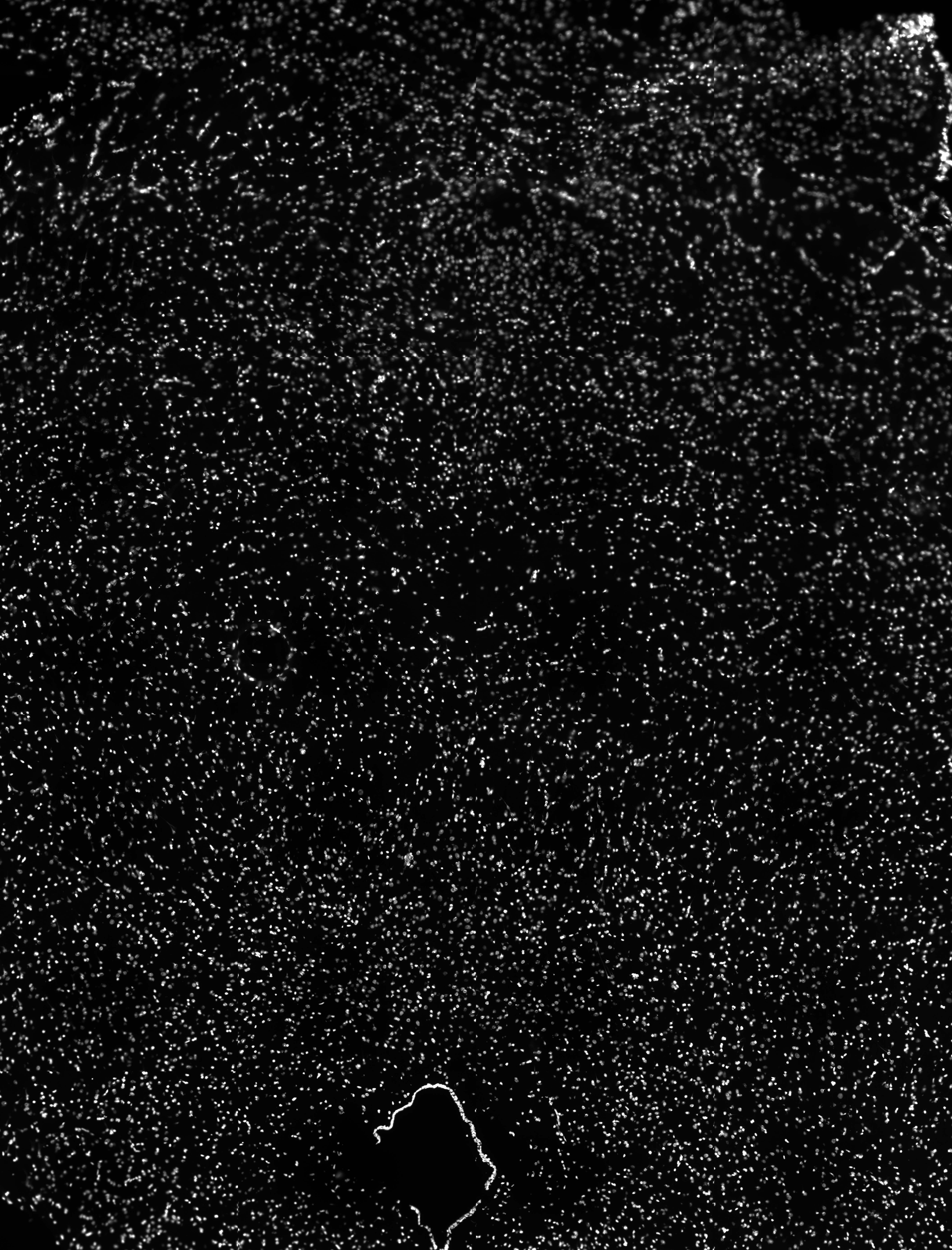

Supplement: Supplementary file 5 — Source Data for Figure 3 [file EMBR-24-e53408-s005.zip › Figure 3/3A/Fig 3A; Hoechst signal, original orientation.jpg]

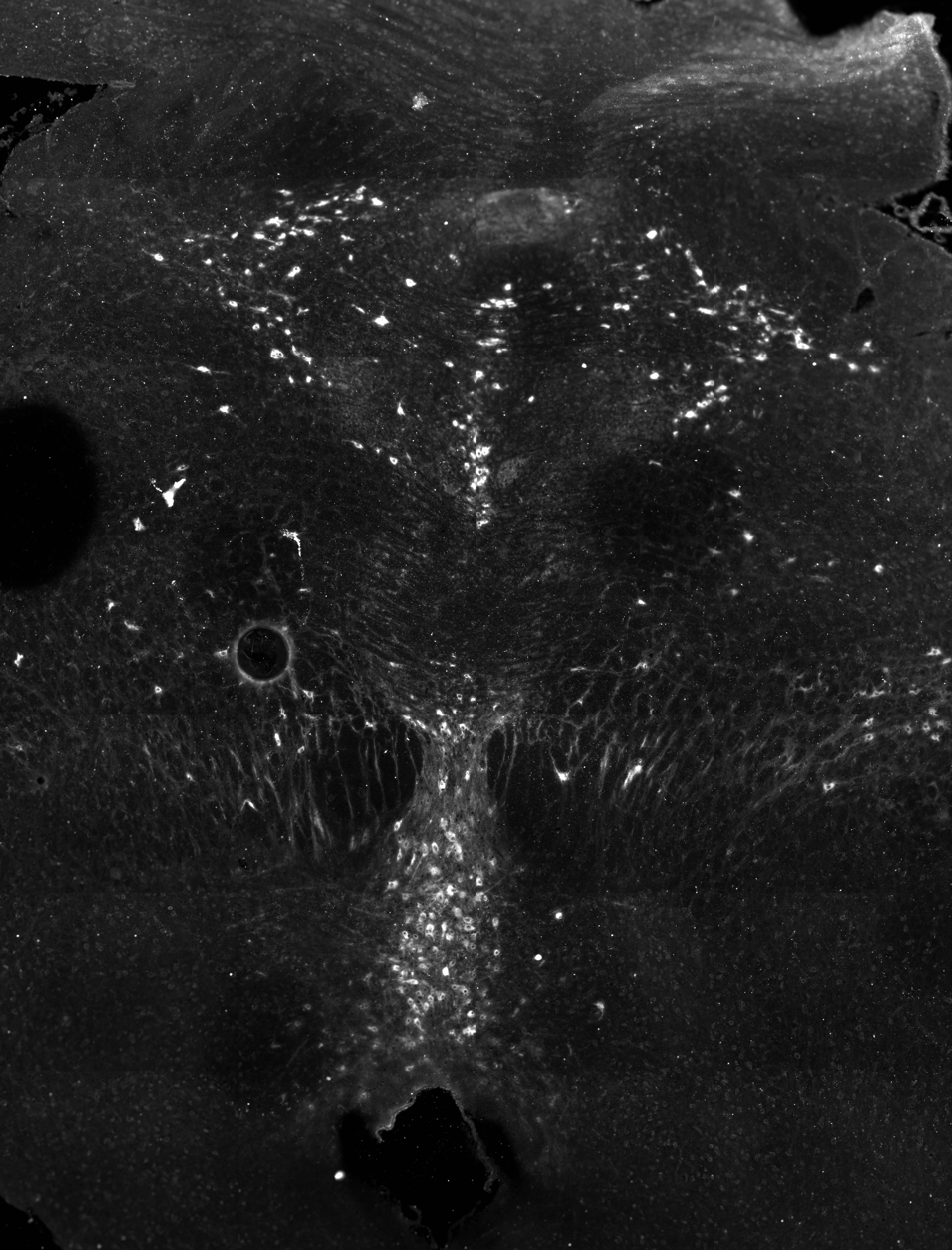

Supplement: Supplementary file 5 — Source Data for Figure 3 [file EMBR-24-e53408-s005.zip › Figure 3/3A/Fig 3A; TPH signal, original orientation.jpg]

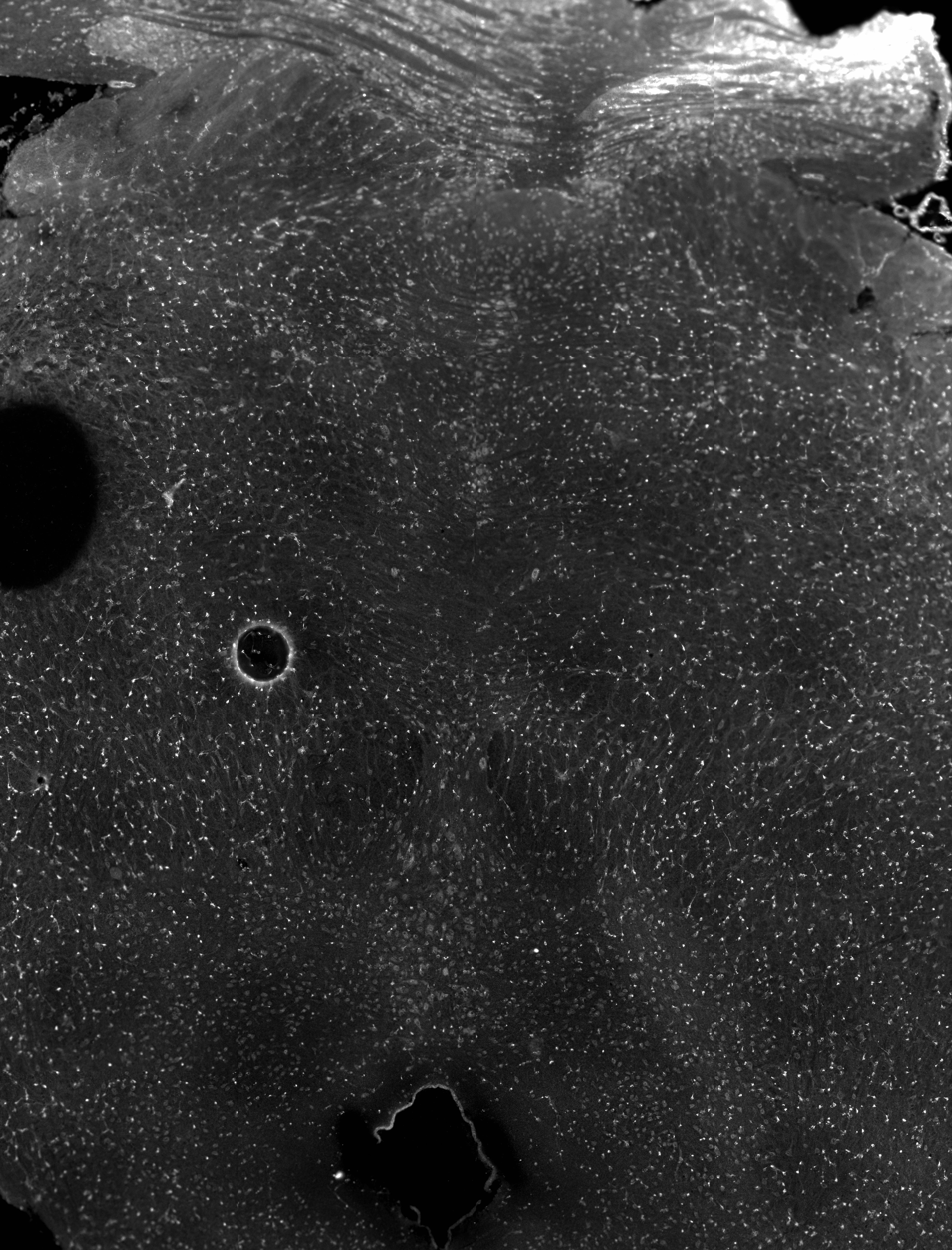

Supplement: Supplementary file 5 — Source Data for Figure 3 [file EMBR-24-e53408-s005.zip › Figure 3/3A/Fig 3A; BubR1 signal, original orientation.jpg]

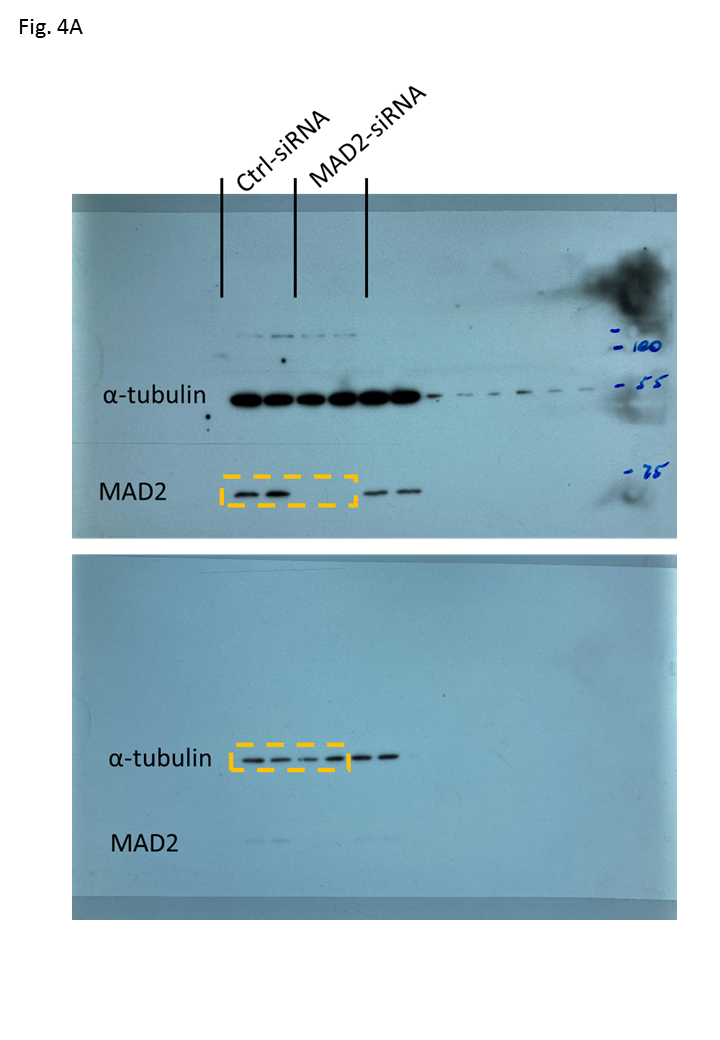

Supplement: Supplementary file 6 — Source Data for Figure 4 [file EMBR-24-e53408-s004.zip › Figure 4/4A/Immunoblot MAD2 + tubulin.tif]

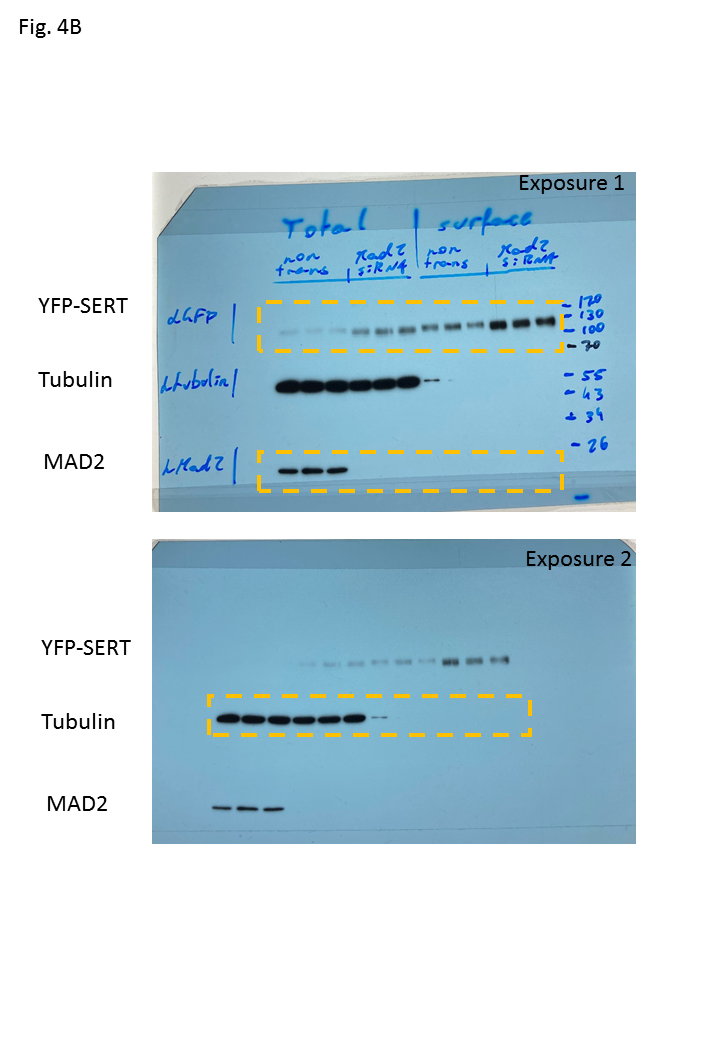

Supplement: Supplementary file 6 — Source Data for Figure 4 [file EMBR-24-e53408-s004.zip › Figure 4/4B/Immunoblot YFP-SERT surface biotinylation.tif]

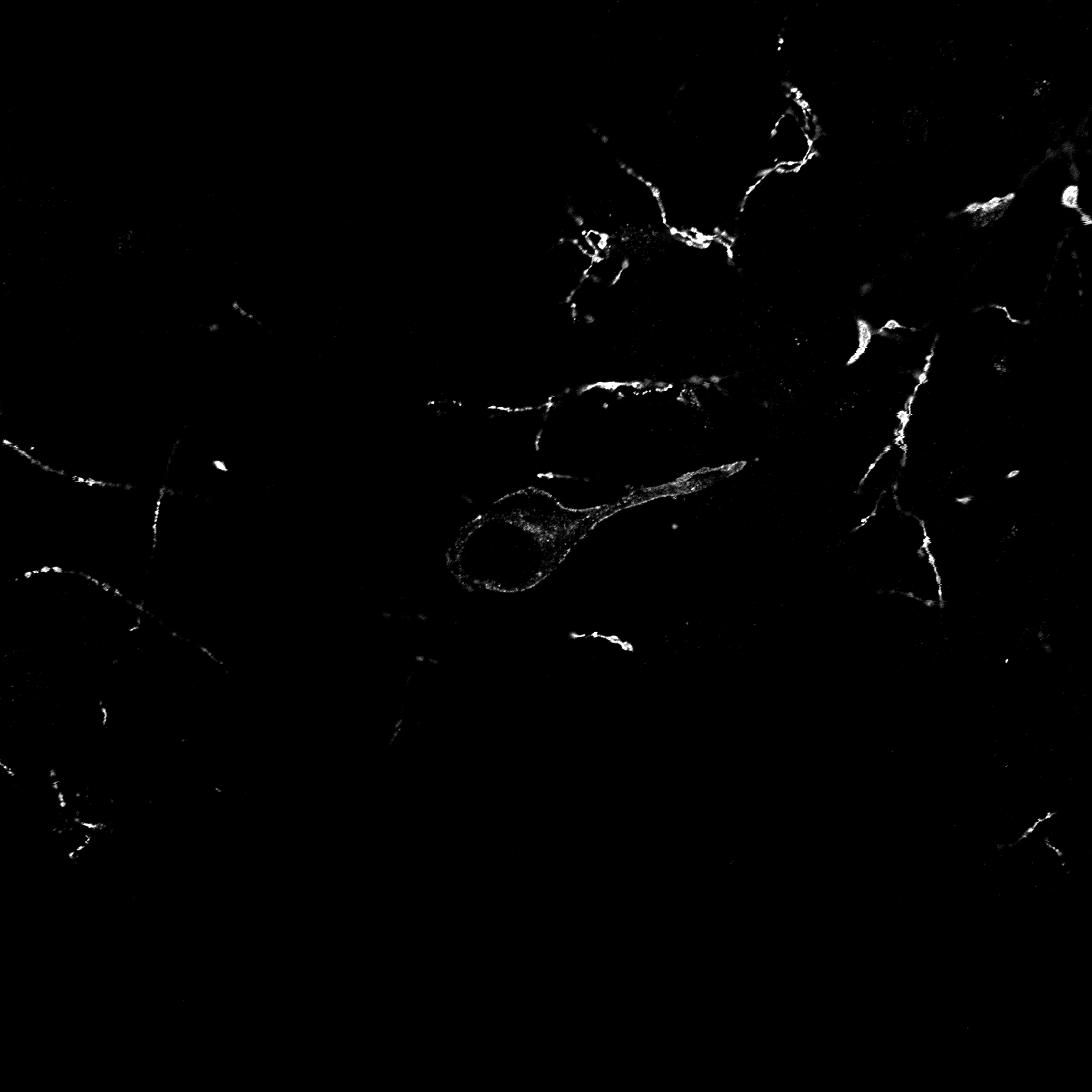

Supplement: Supplementary file 7 — Source Data for Figure 5 [file EMBR-24-e53408-s006.zip › Figure 5/5F/Fig 5F; 60x MAD2-shRNA, SERT-signal example image 4.tif]

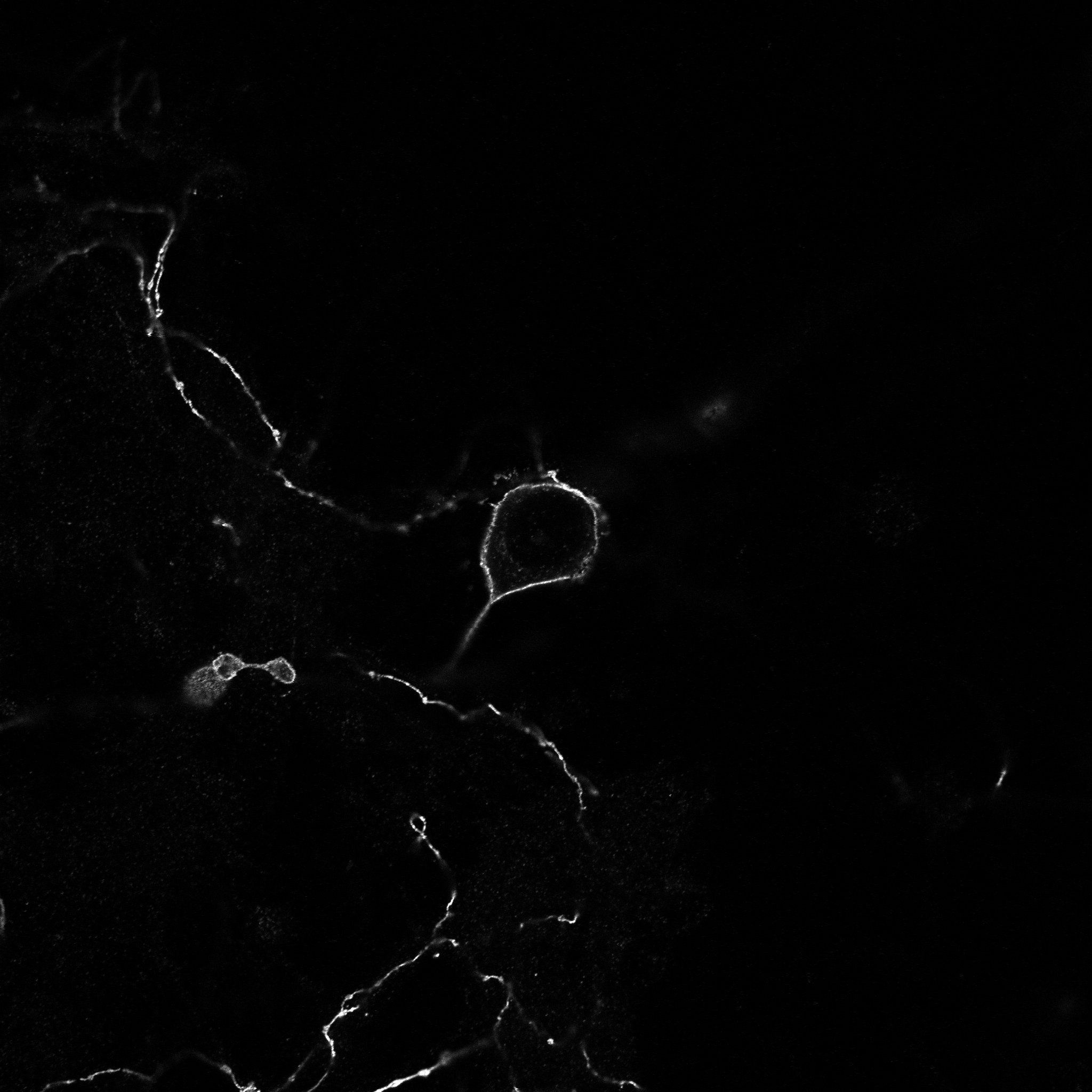

Supplement: Supplementary file 7 — Source Data for Figure 5 [file EMBR-24-e53408-s006.zip › Figure 5/5F/Fig 5F; 60x MAD2-shRNA, SERT-signal example image 1.tif]

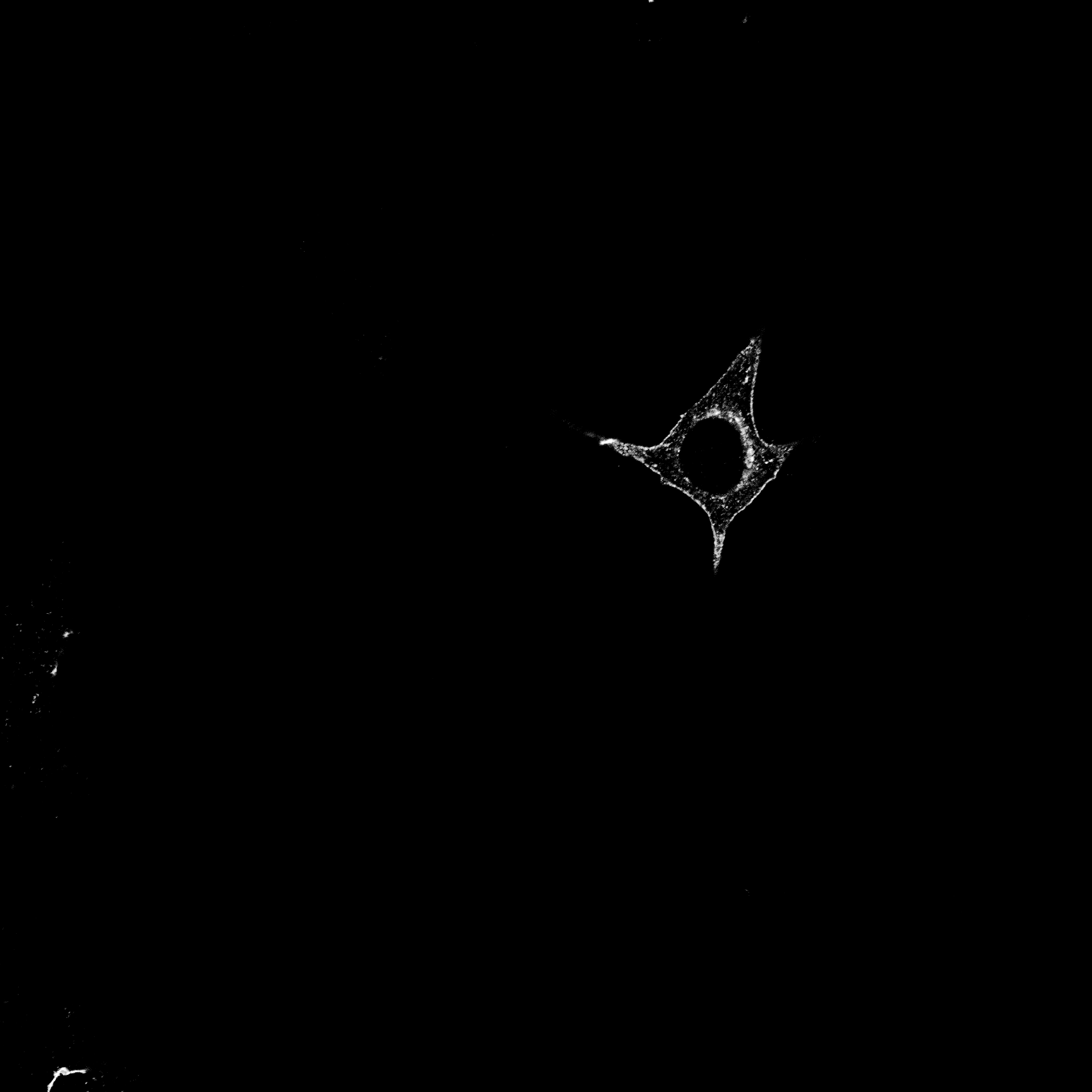

Supplement: Supplementary file 7 — Source Data for Figure 5 [file EMBR-24-e53408-s006.zip › Figure 5/5F/Fig 5F; 60x MAD2-shRNA, SERT-signal example image 3.tif]

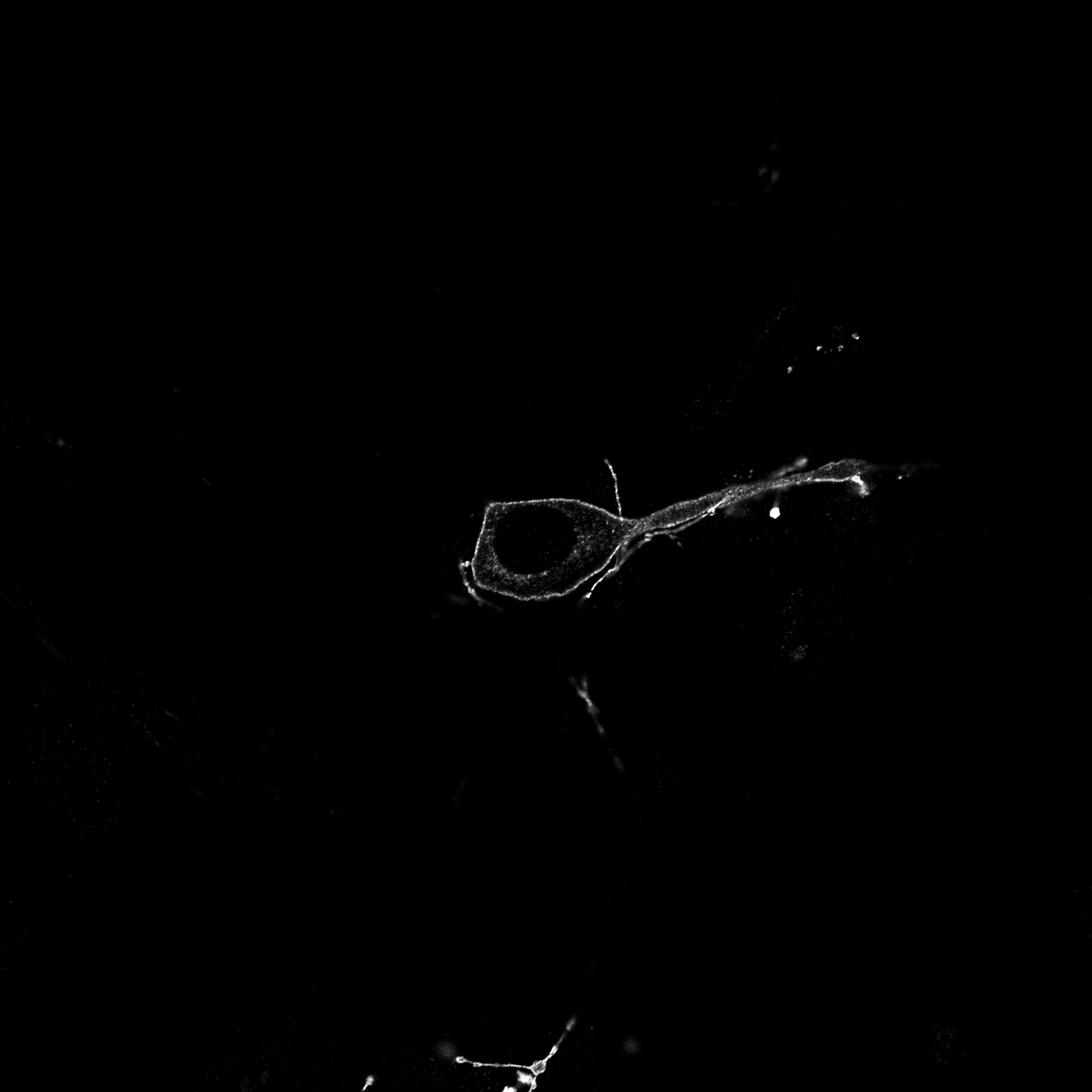

Supplement: Supplementary file 7 — Source Data for Figure 5 [file EMBR-24-e53408-s006.zip › Figure 5/5F/Fig 5F; 60x MAD2-shRNA, SERT-signal example image 2.tif]

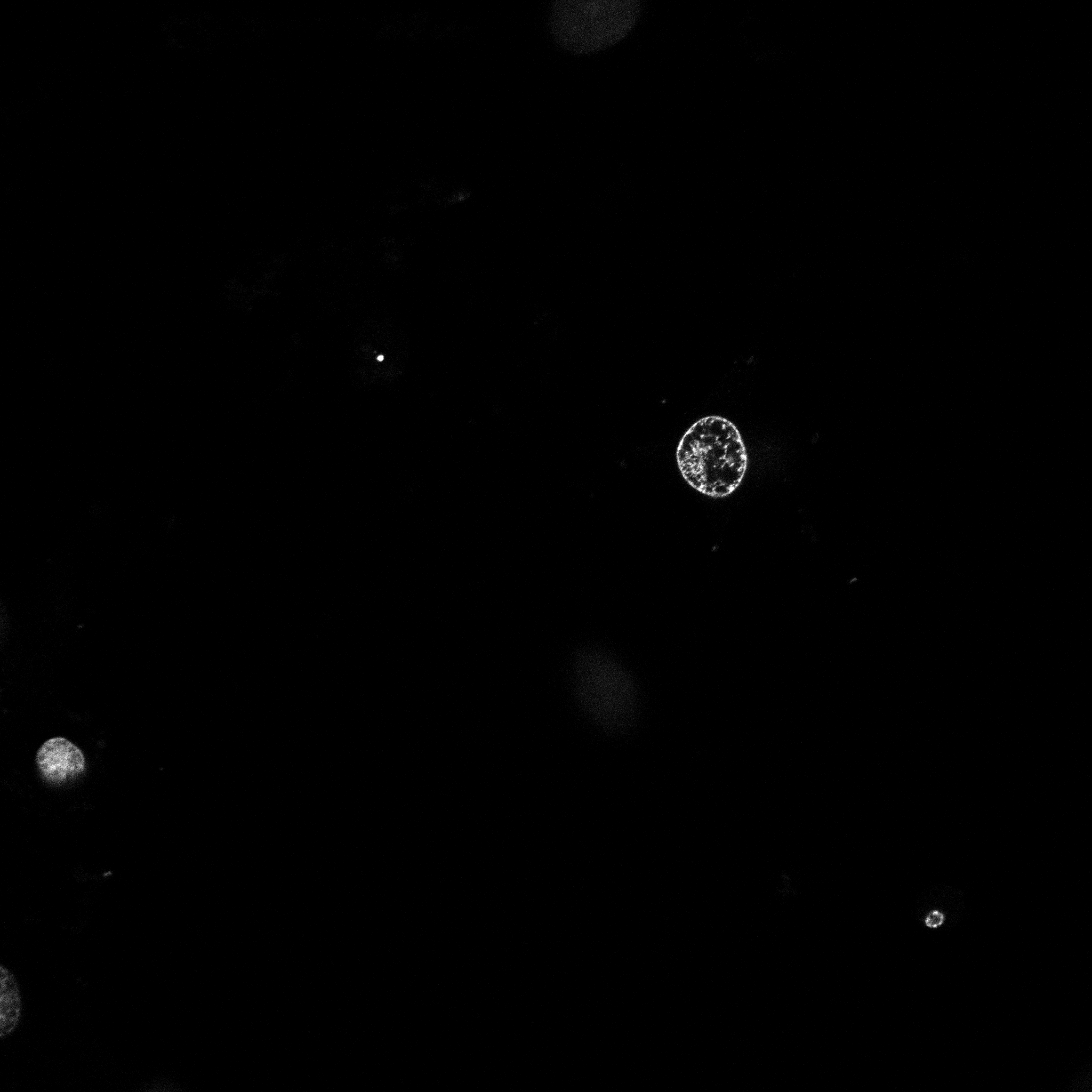

Supplement: Supplementary file 7 — Source Data for Figure 5 [file EMBR-24-e53408-s006.zip › Figure 5/5F/Fig 5F; 60x MAD2-shRNA, HOECHST-signal example image 3.tif]

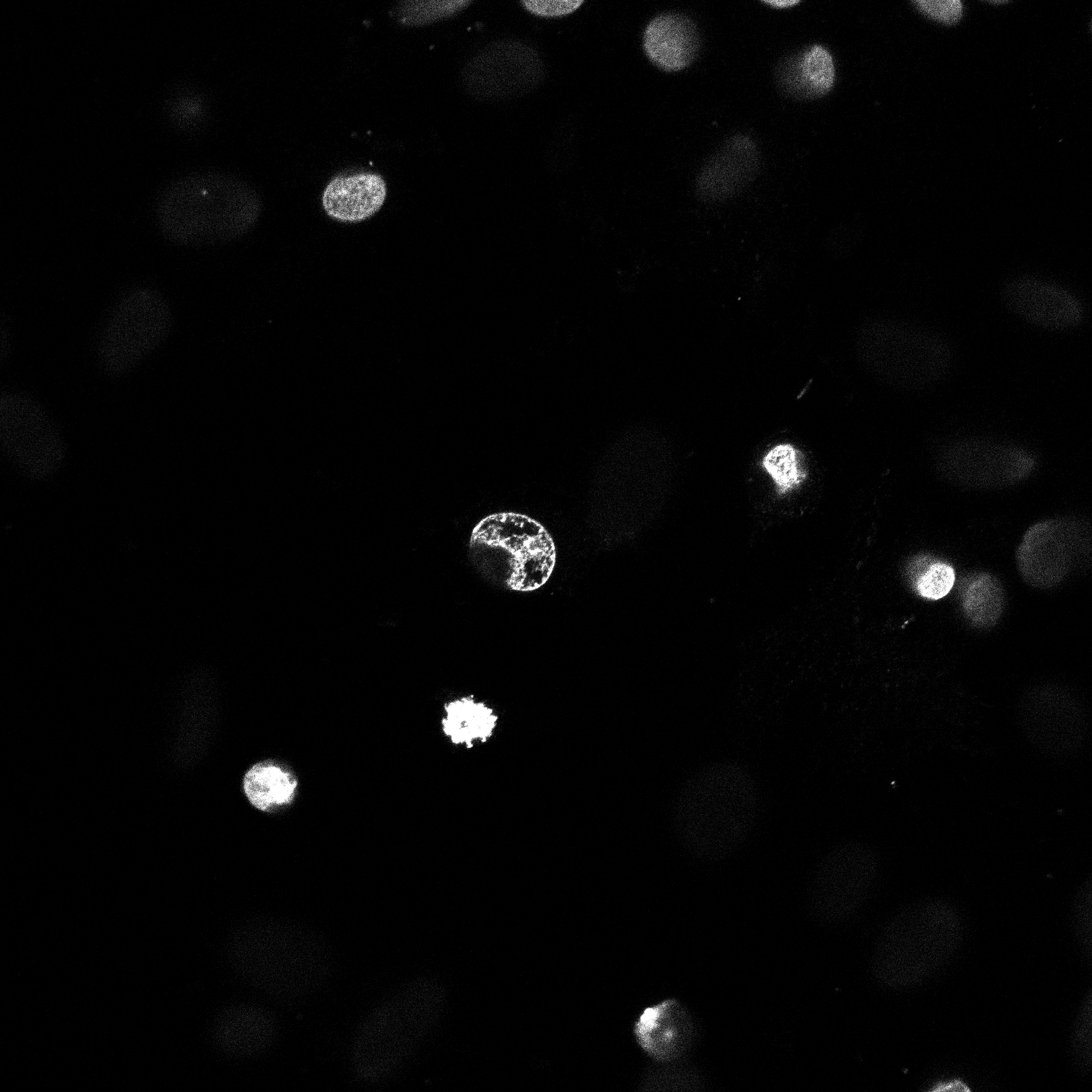

Supplement: Supplementary file 7 — Source Data for Figure 5 [file EMBR-24-e53408-s006.zip › Figure 5/5F/Fig 5F; 60x scramble-shRNA, HOECHST-signal example image 2.tif]

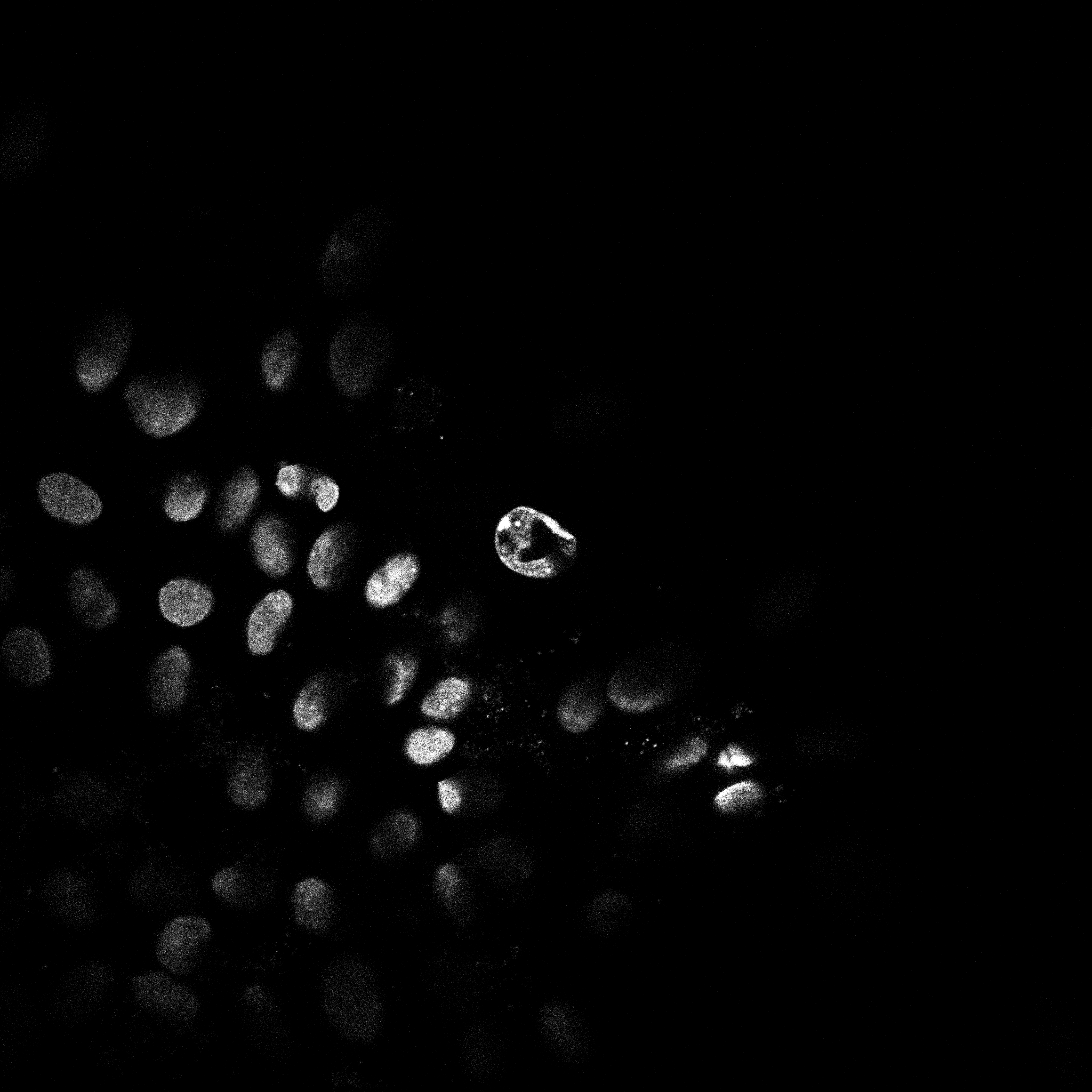

Supplement: Supplementary file 7 — Source Data for Figure 5 [file EMBR-24-e53408-s006.zip › Figure 5/5F/Fig 5F; 60x scramble-shRNA, HOECHST-signal example image 3.tif]

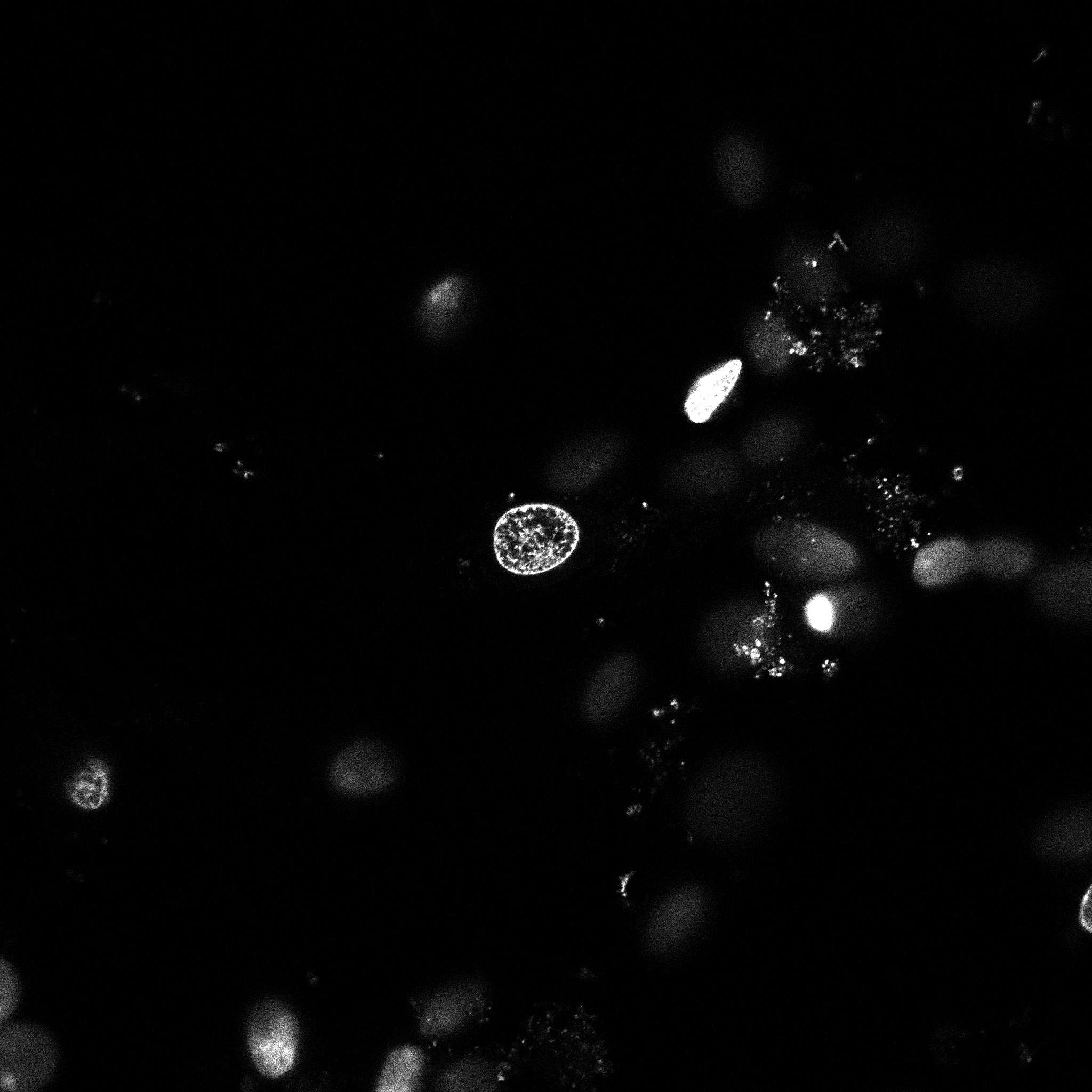

Supplement: Supplementary file 7 — Source Data for Figure 5 [file EMBR-24-e53408-s006.zip › Figure 5/5F/Fig 5F; 60x MAD2-shRNA, HOECHST-signal example image 2.tif]

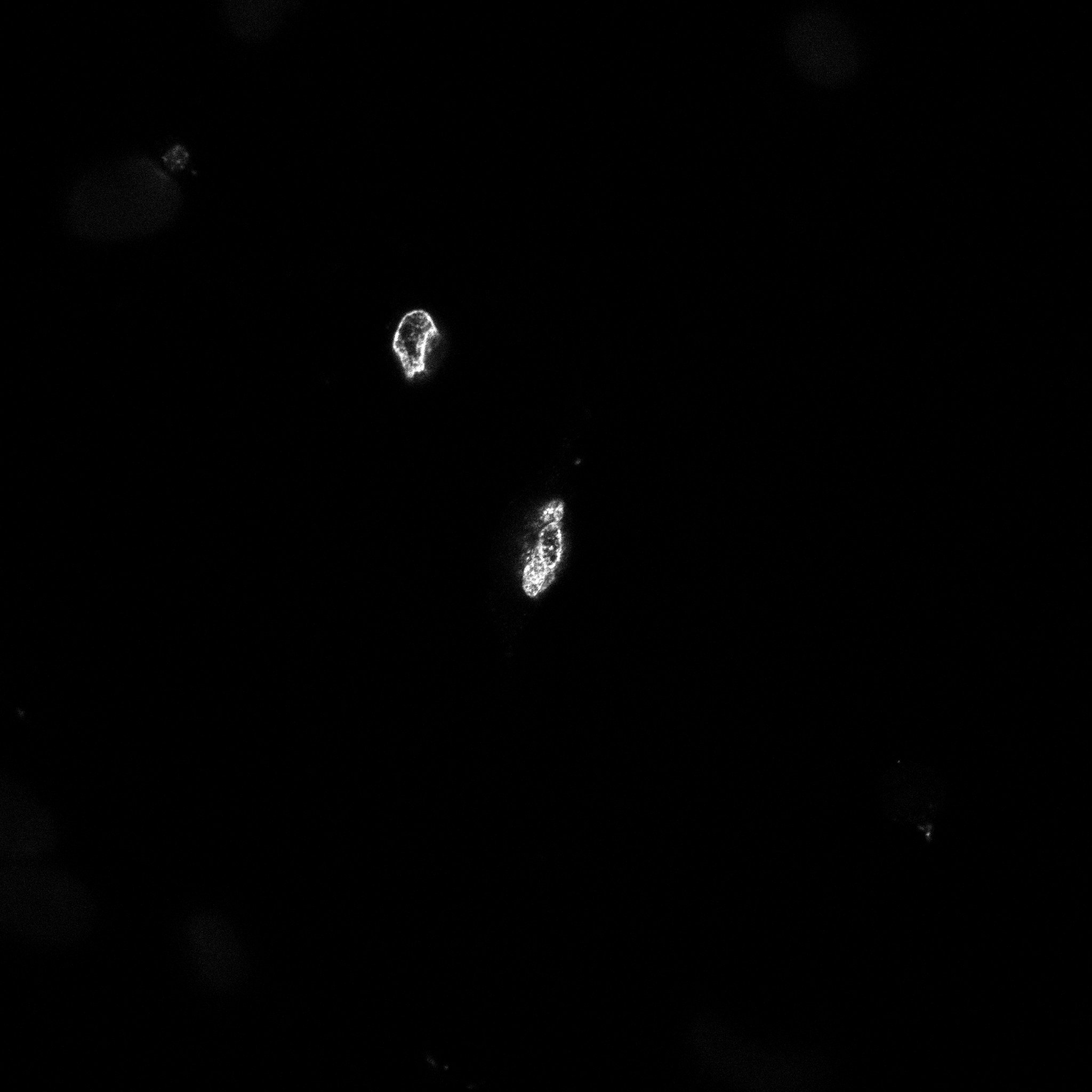

Supplement: Supplementary file 7 — Source Data for Figure 5 [file EMBR-24-e53408-s006.zip › Figure 5/5F/Fig 5F; 60x scramble-shRNA, HOECHST-signal example image 1.tif]

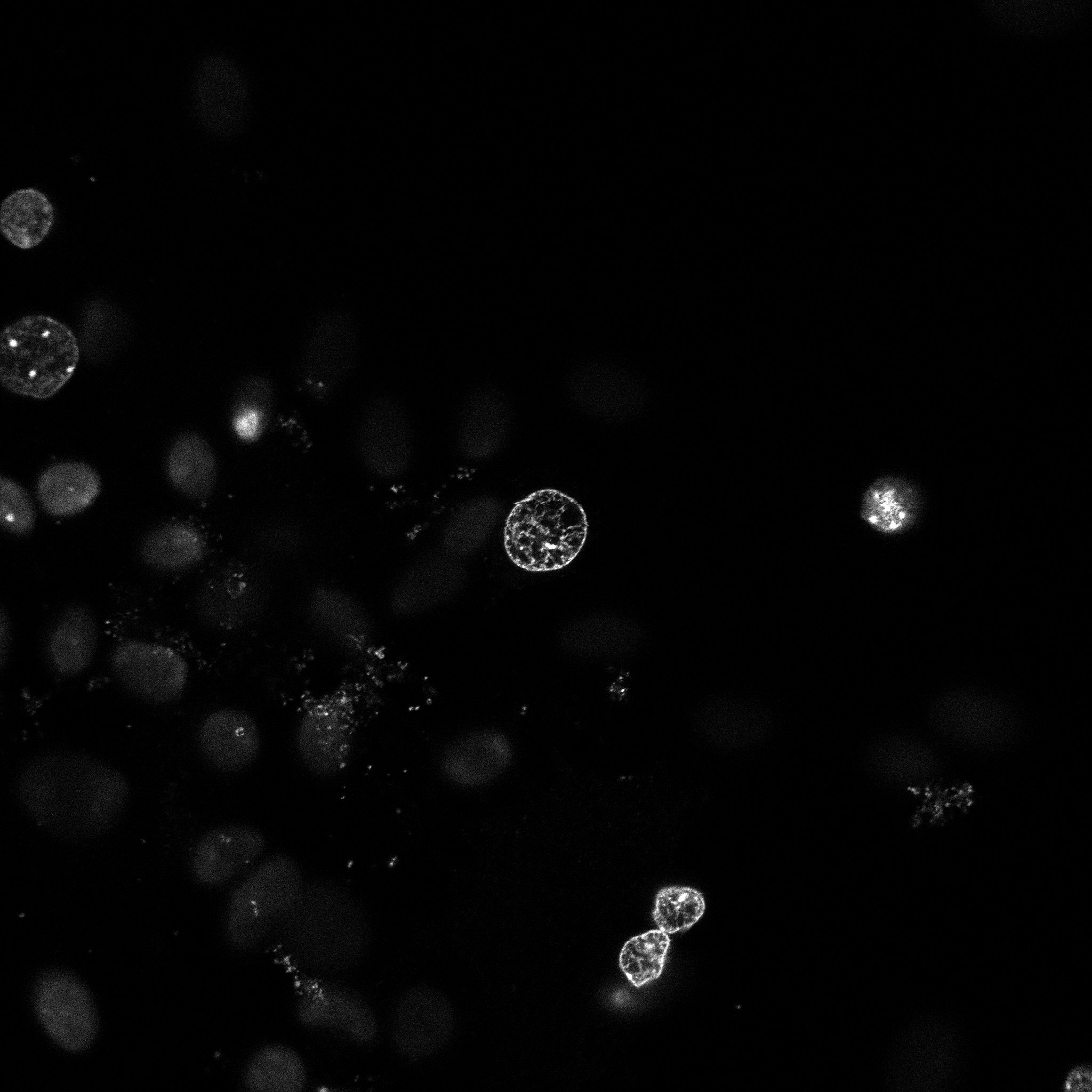

Supplement: Supplementary file 7 — Source Data for Figure 5 [file EMBR-24-e53408-s006.zip › Figure 5/5F/Fig 5F; 60x MAD2-shRNA, HOECHST-signal example image 1.tif]

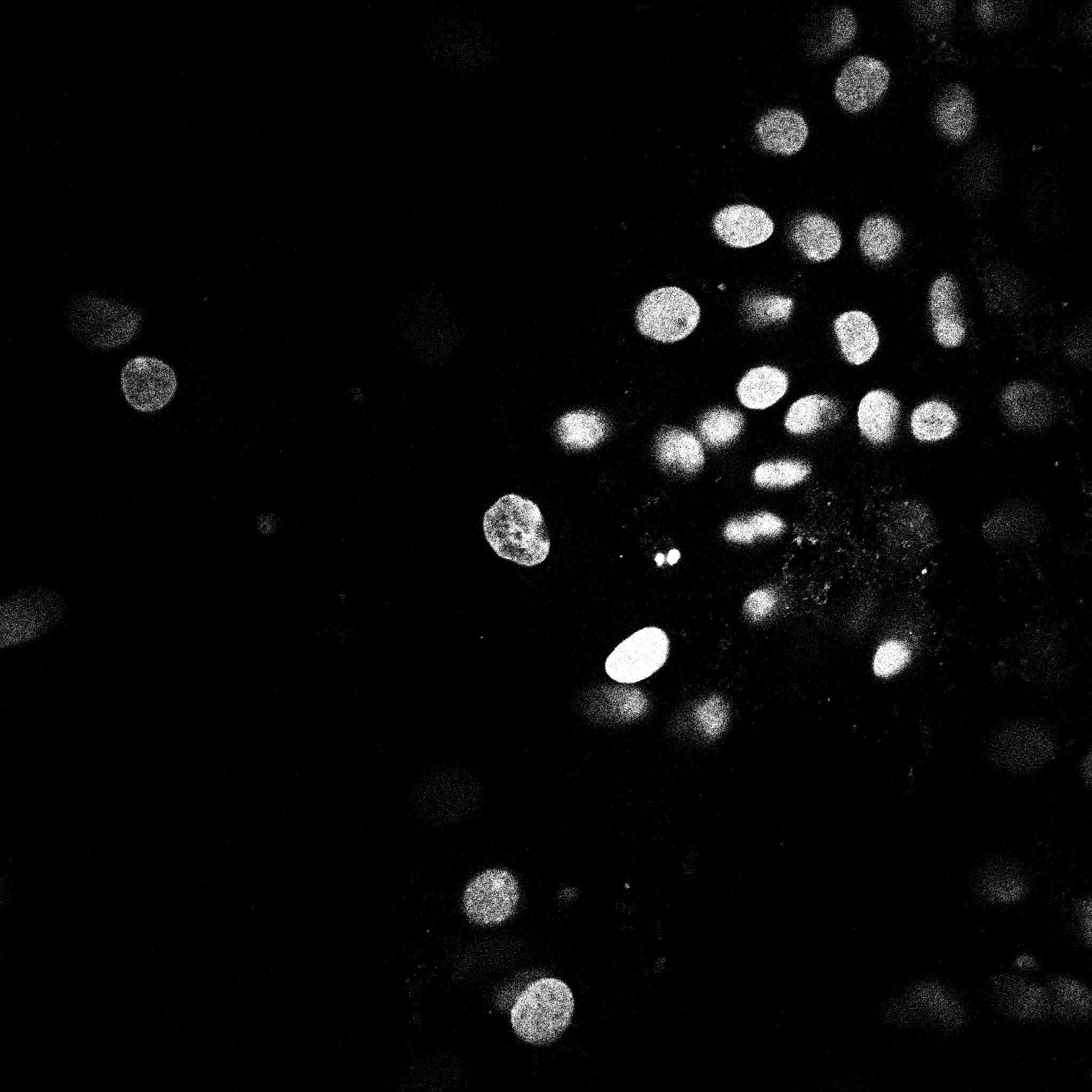

Supplement: Supplementary file 7 — Source Data for Figure 5 [file EMBR-24-e53408-s006.zip › Figure 5/5F/Fig 5F; 60x scramble-shRNA, HOECHST-signal example image 4.tif]

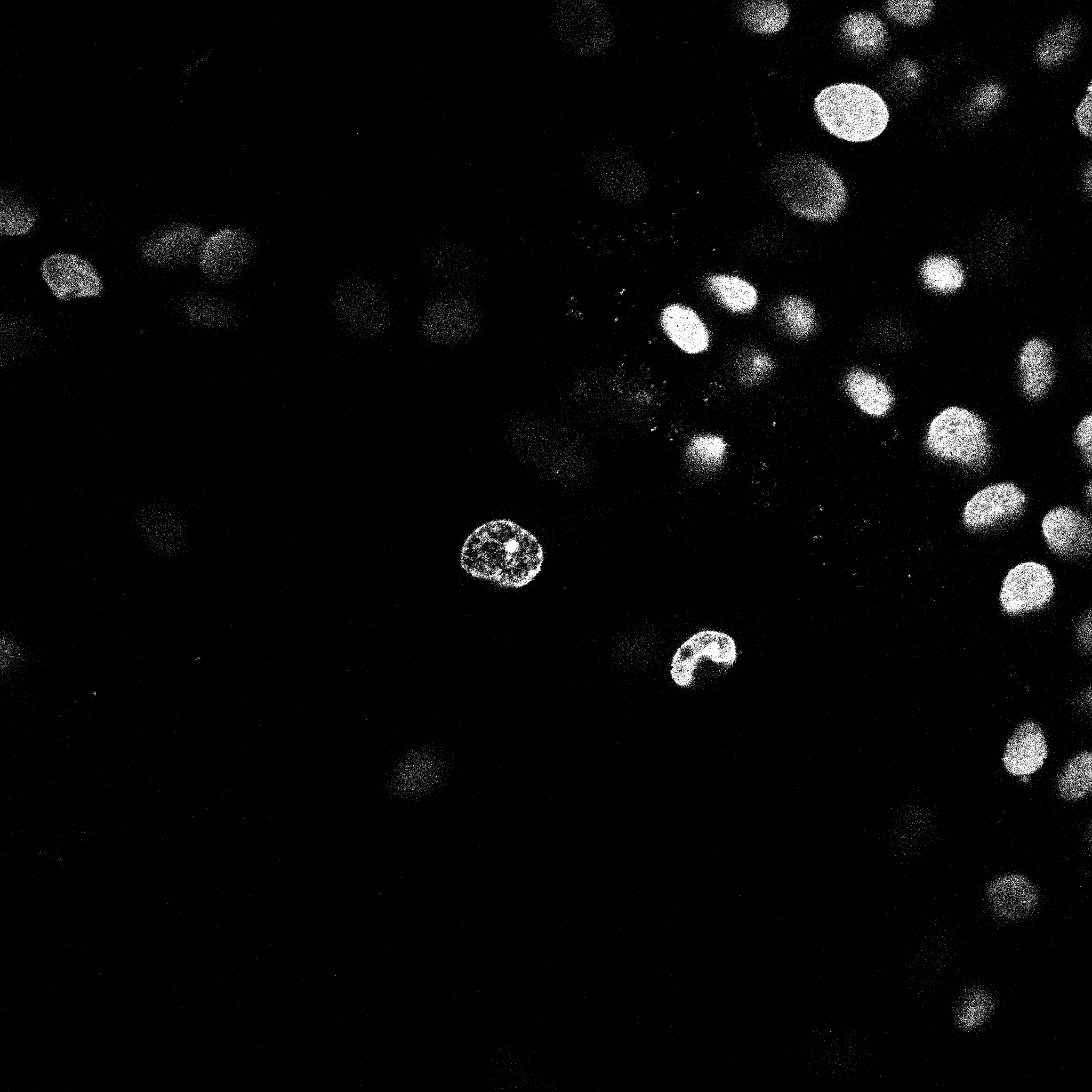

Supplement: Supplementary file 7 — Source Data for Figure 5 [file EMBR-24-e53408-s006.zip › Figure 5/5F/Fig 5F; 60x MAD2-shRNA, HOECHST-signal example image 4.tif]

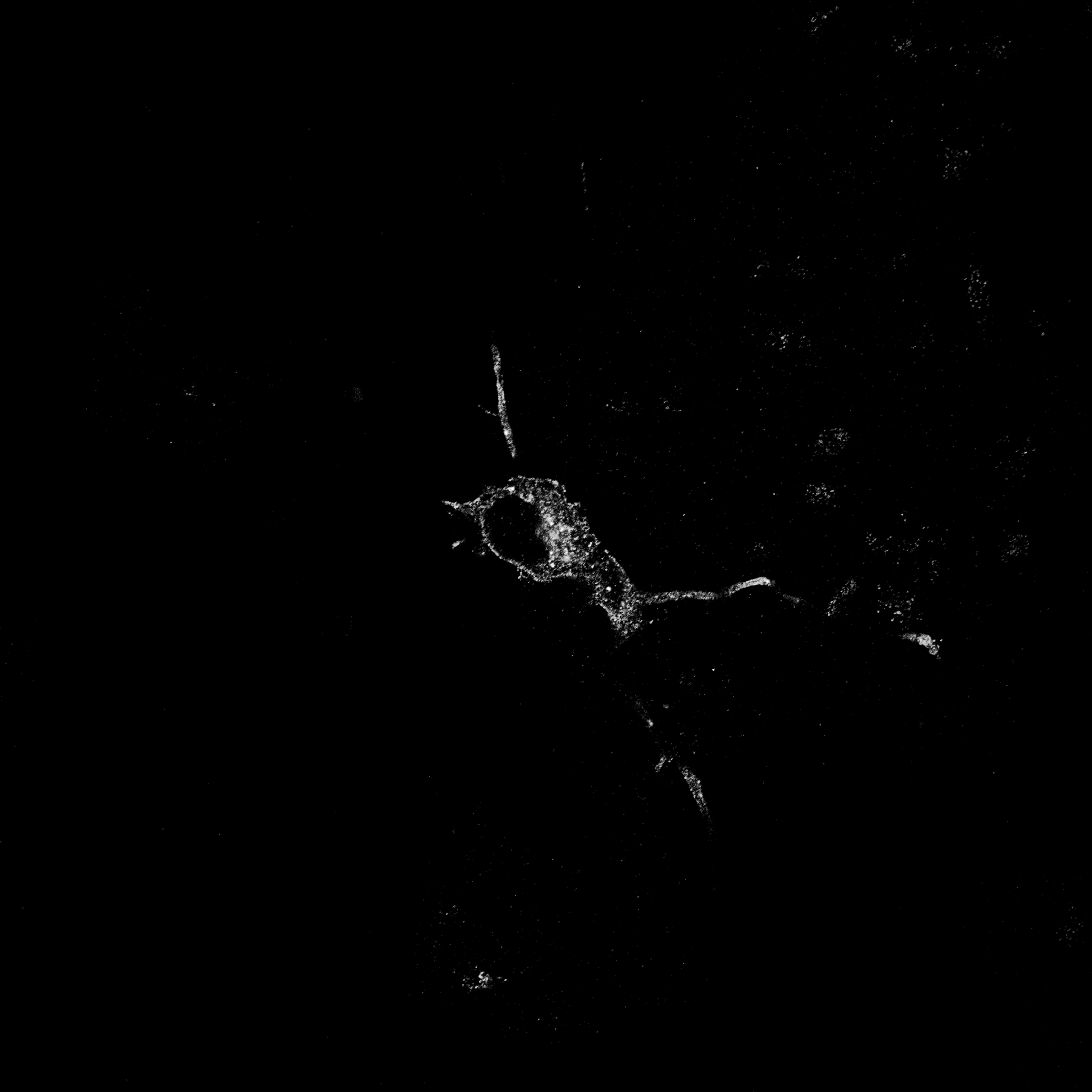

Supplement: Supplementary file 7 — Source Data for Figure 5 [file EMBR-24-e53408-s006.zip › Figure 5/5F/Fig 5F; 60x scramble-shRNA, SERT-signal example image 4.tif]

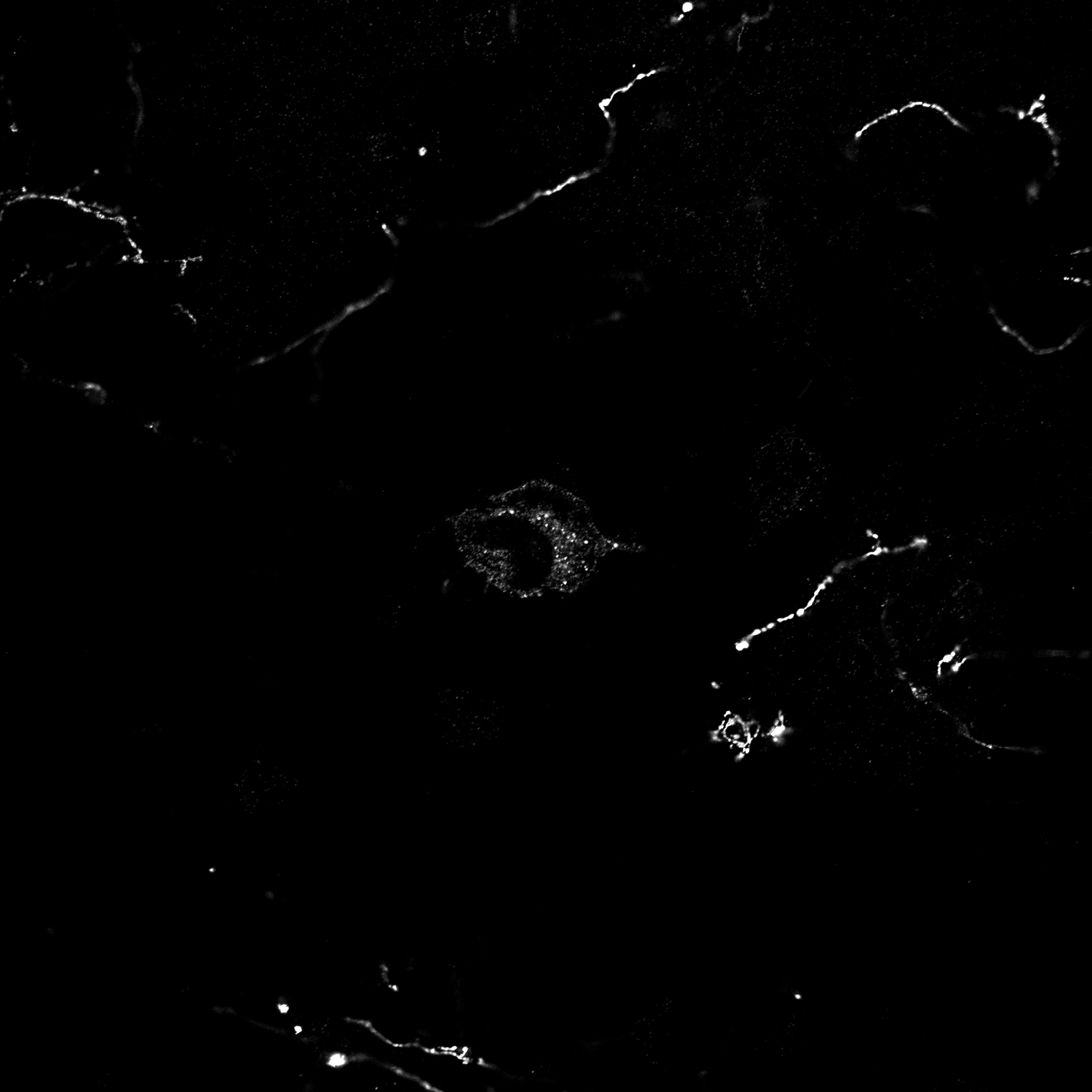

Supplement: Supplementary file 7 — Source Data for Figure 5 [file EMBR-24-e53408-s006.zip › Figure 5/5F/Fig 5F; 60x scramble-shRNA, SERT-signal example image 2.tif]

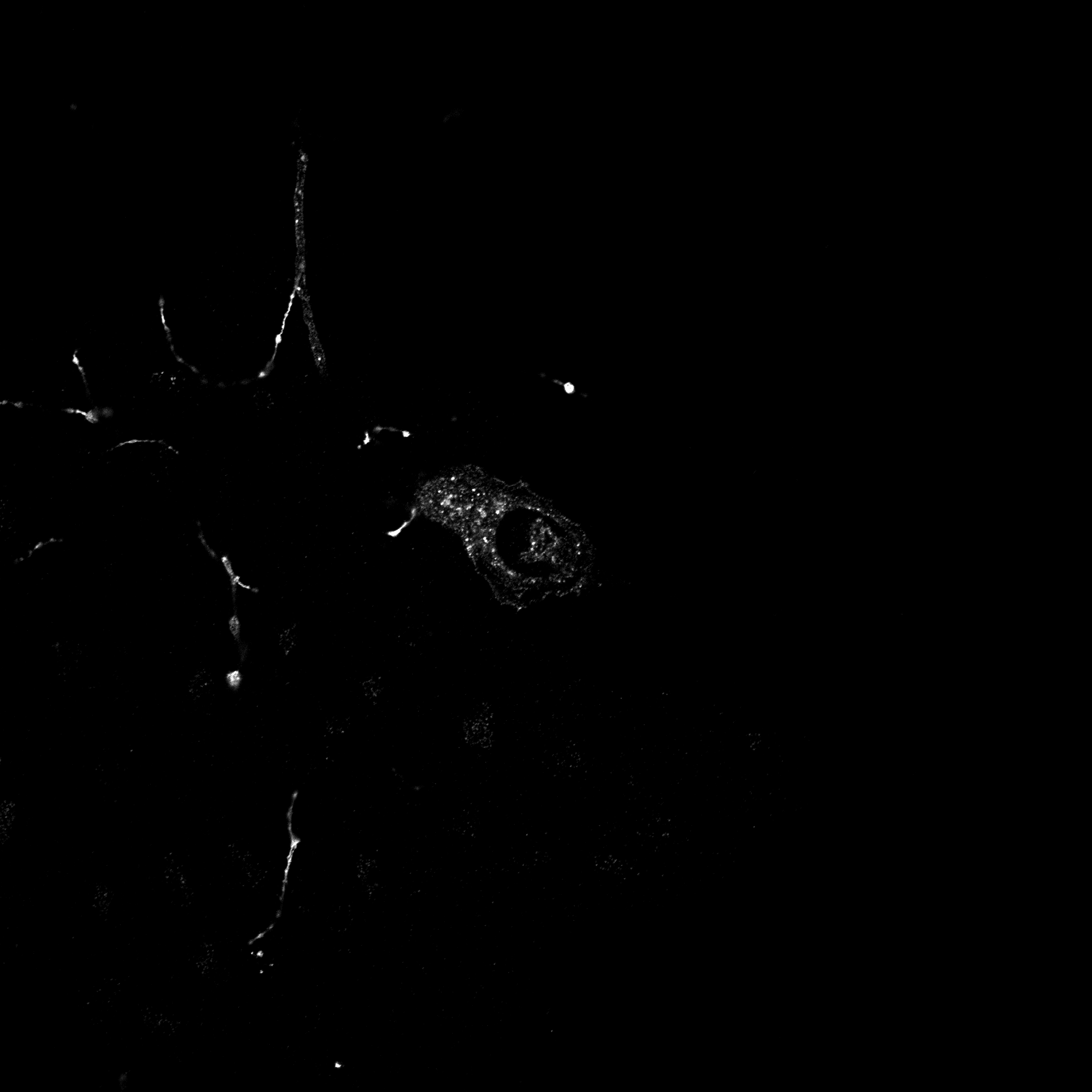

Supplement: Supplementary file 7 — Source Data for Figure 5 [file EMBR-24-e53408-s006.zip › Figure 5/5F/Fig 5F; 60x scramble-shRNA, SERT-signal example image 3.tif]

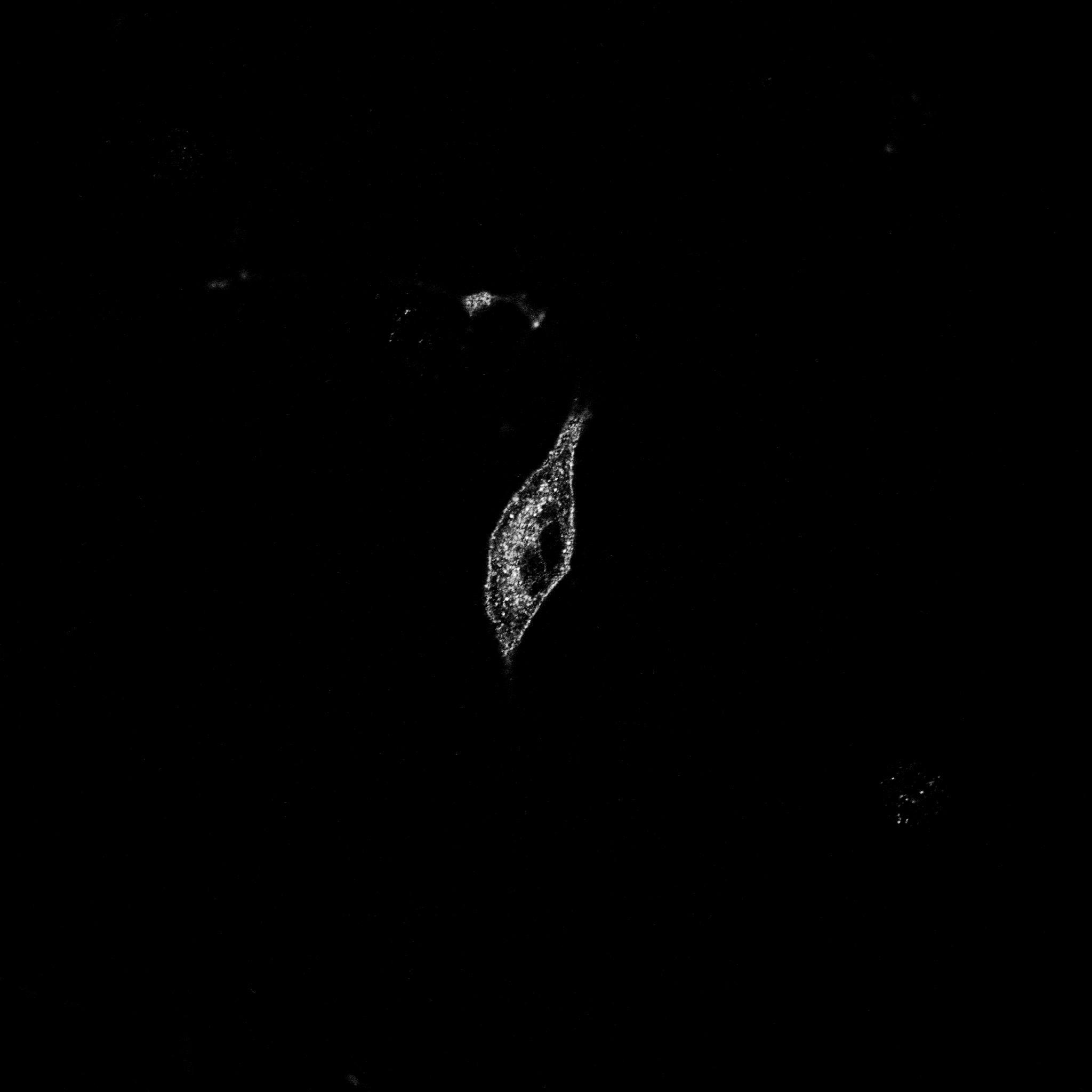

Supplement: Supplementary file 7 — Source Data for Figure 5 [file EMBR-24-e53408-s006.zip › Figure 5/5F/Fig 5F; 60x scramble-shRNA, SERT-signal example image 1.tif]

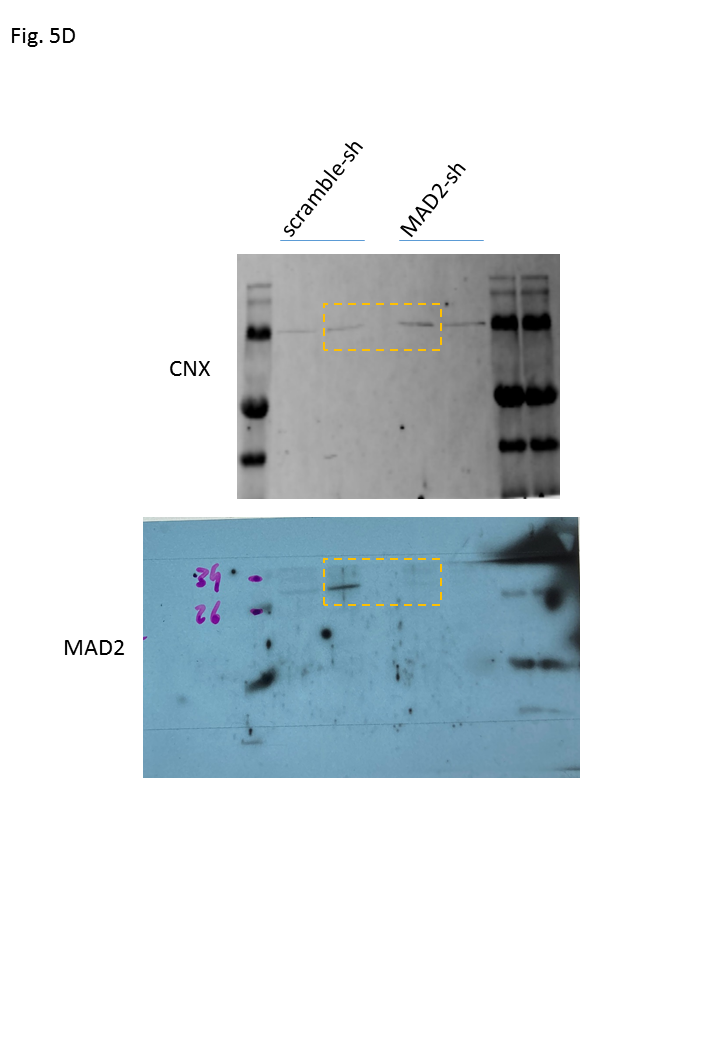

Supplement: Supplementary file 7 — Source Data for Figure 5 [file EMBR-24-e53408-s006.zip › Figure 5/5D/Immunoblot rMAD2 + rCNX.tif]

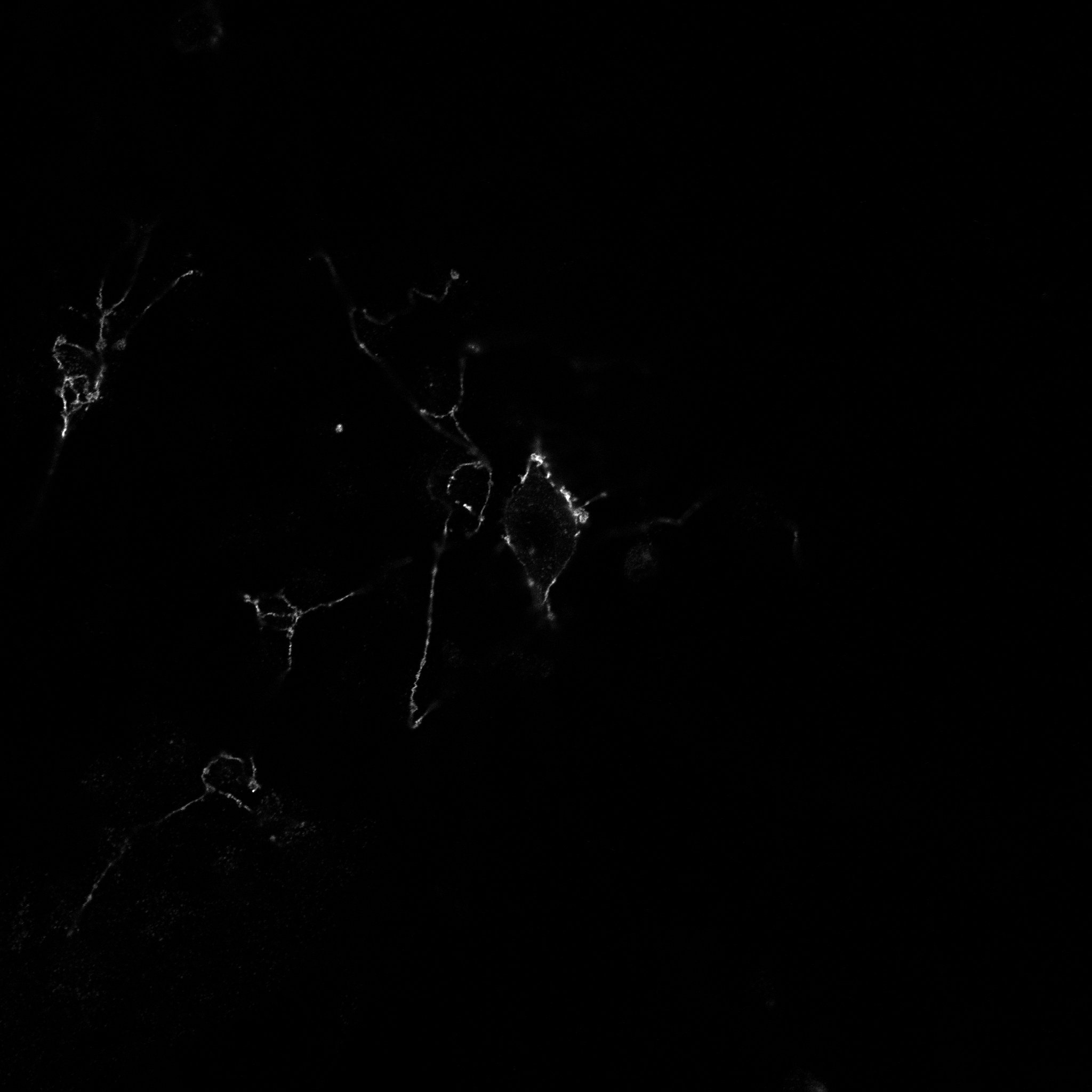

Supplement: Supplementary file 7 — Source Data for Figure 5 [file EMBR-24-e53408-s006.zip › Figure 5/5E/Fig 5E; 60x MAD2-shRNA, SERT-signal.tif]

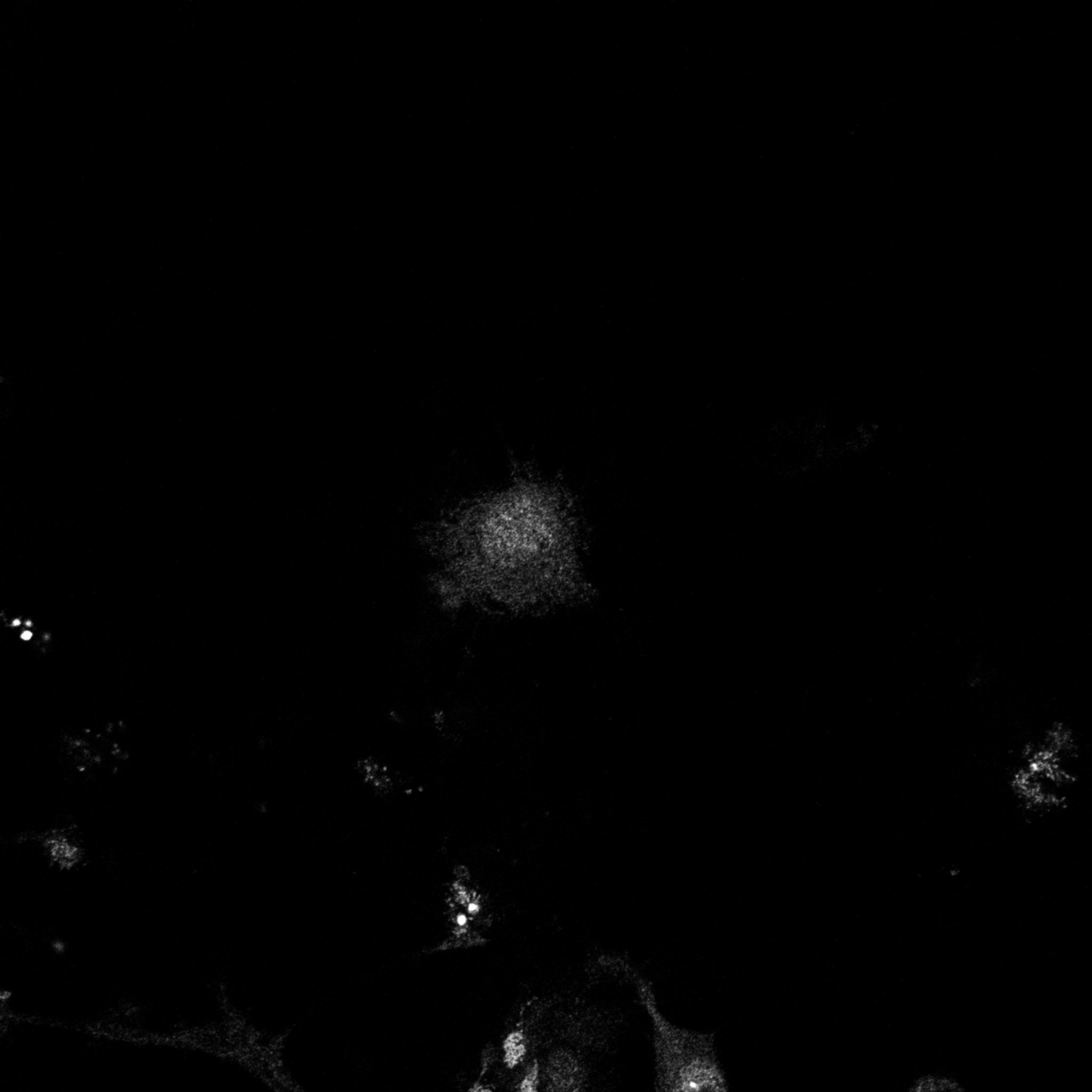

Supplement: Supplementary file 7 — Source Data for Figure 5 [file EMBR-24-e53408-s006.zip › Figure 5/5E/Fig 5E; 60x scramble-shRNA, MAD2-signal.tif]

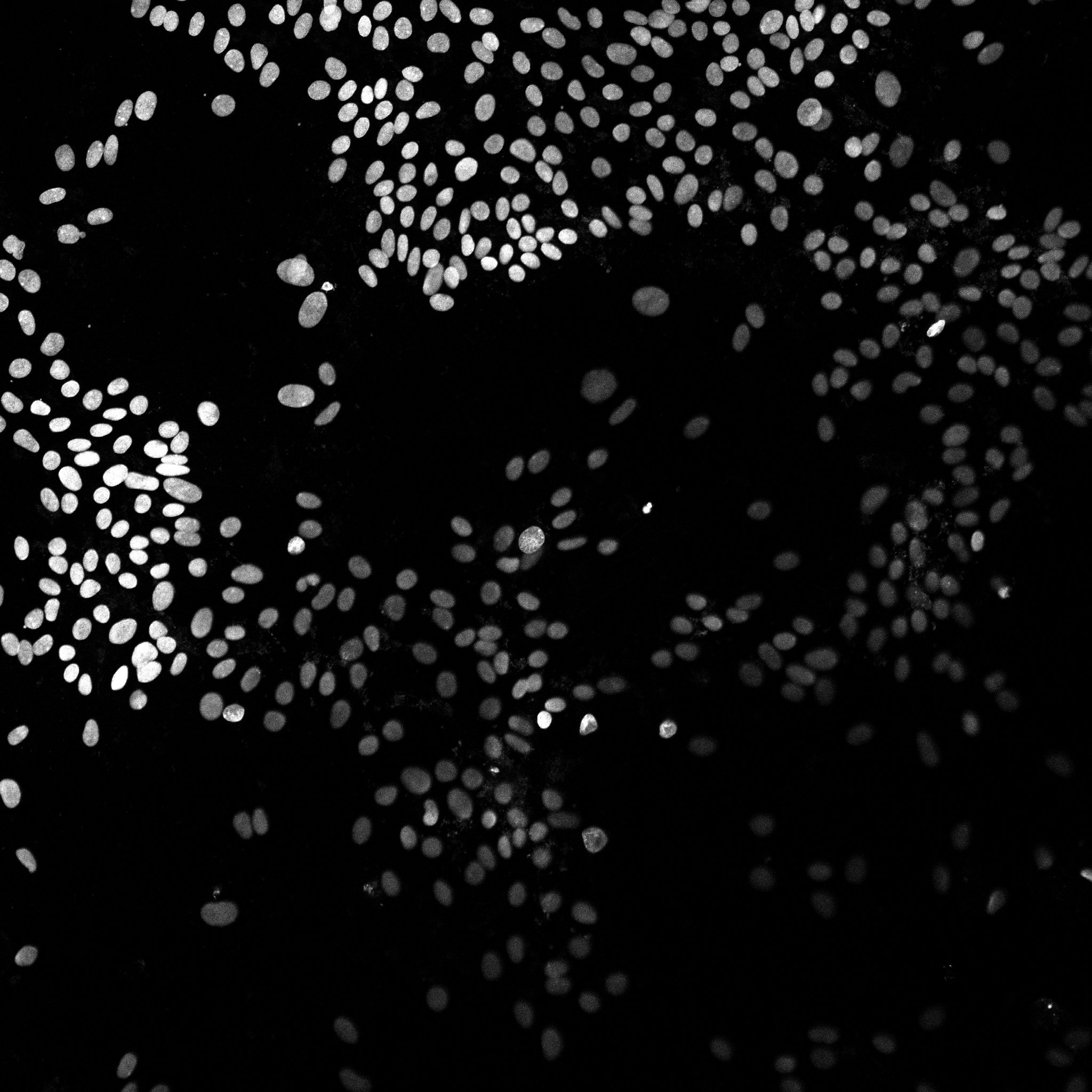

Supplement: Supplementary file 7 — Source Data for Figure 5 [file EMBR-24-e53408-s006.zip › Figure 5/5E/Fig 5E; 20x scramble-shRNA, Hoechst-signal.tif]

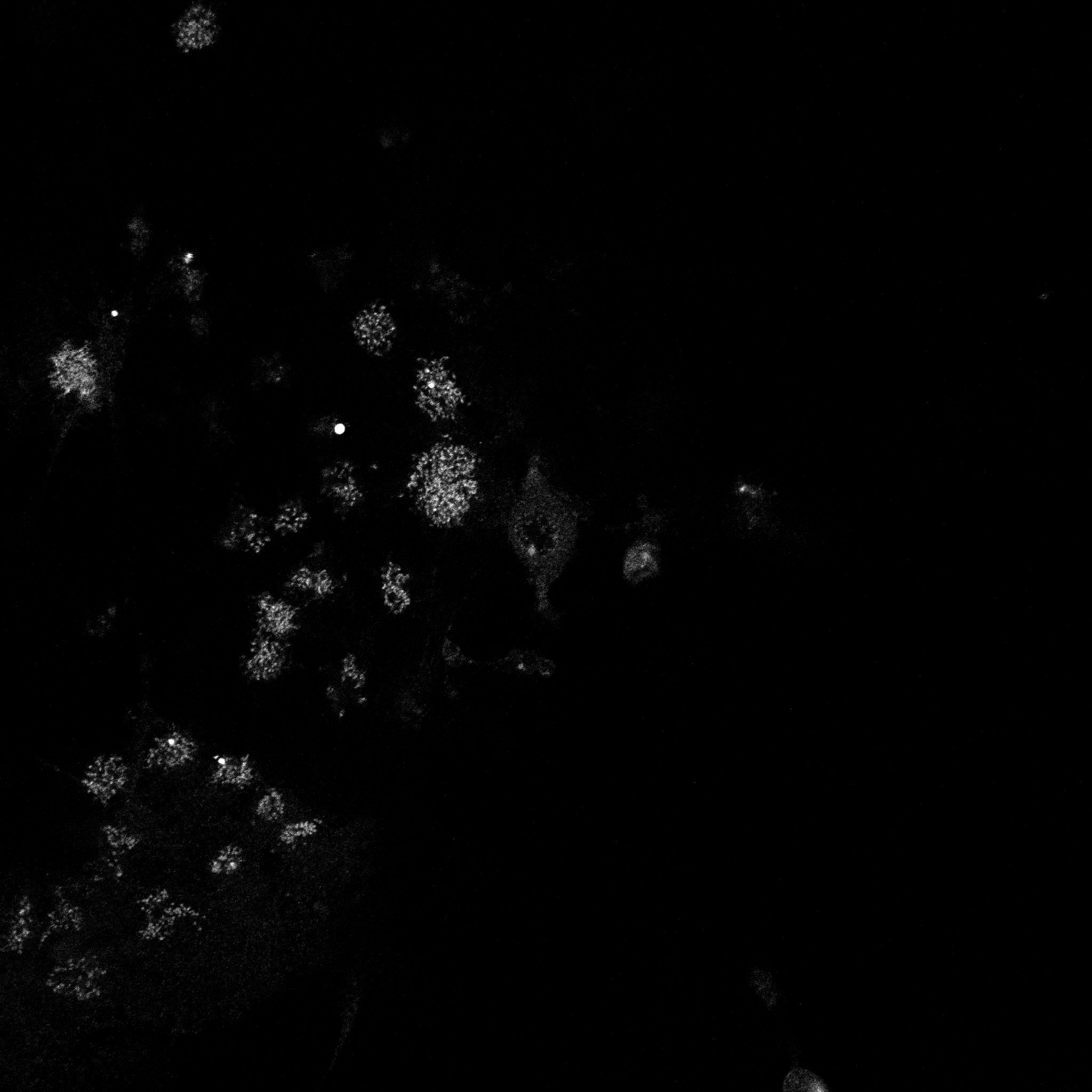

Supplement: Supplementary file 7 — Source Data for Figure 5 [file EMBR-24-e53408-s006.zip › Figure 5/5E/Fig 5E; 60x MAD2-shRNA, MAD2-signal.tif]

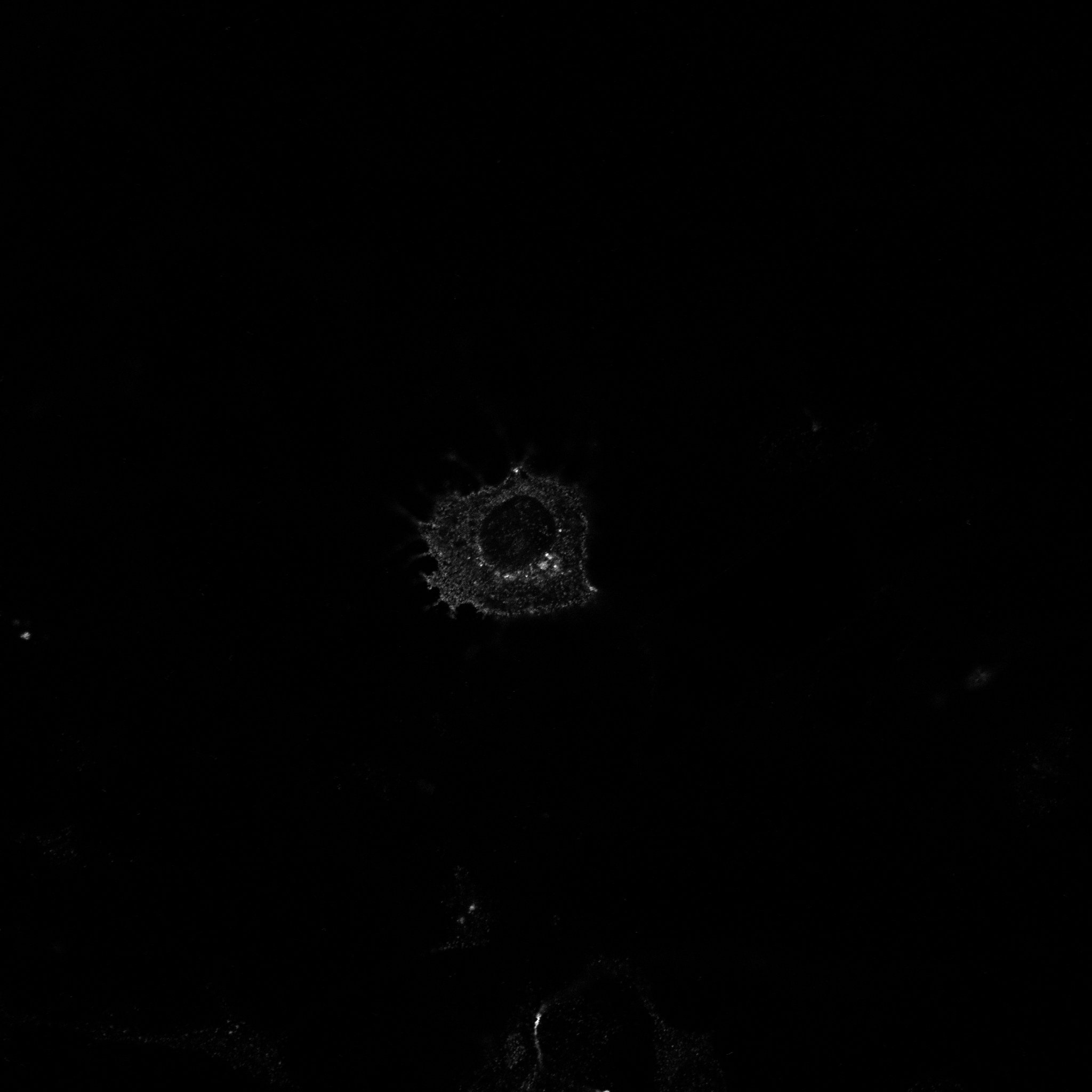

Supplement: Supplementary file 7 — Source Data for Figure 5 [file EMBR-24-e53408-s006.zip › Figure 5/5E/Fig 5E; 60x scramble-shRNA, SERT-signal.tif]

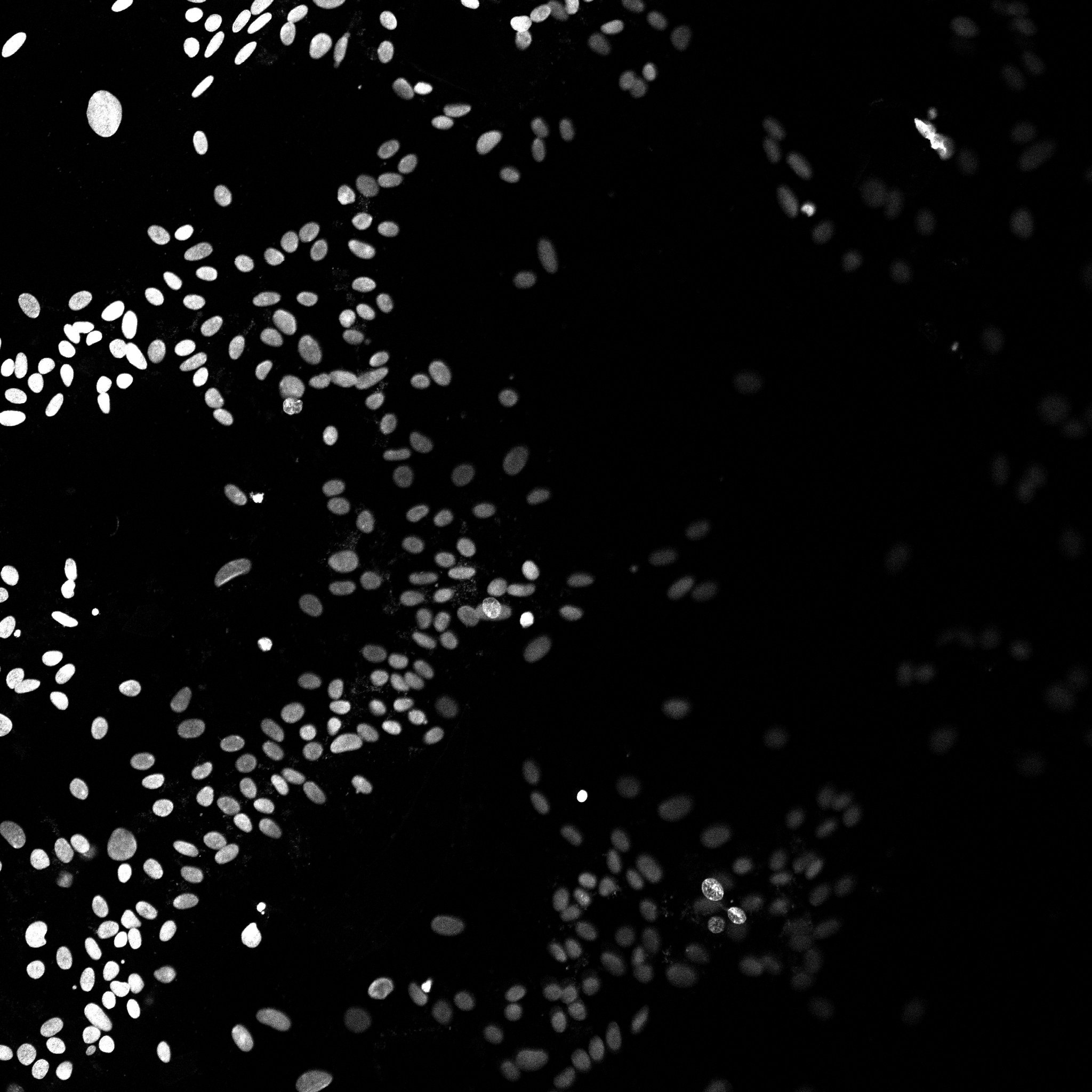

Supplement: Supplementary file 7 — Source Data for Figure 5 [file EMBR-24-e53408-s006.zip › Figure 5/5E/Fig 5E; 20x MAD2-shRNA, Hoechst signal.tif]

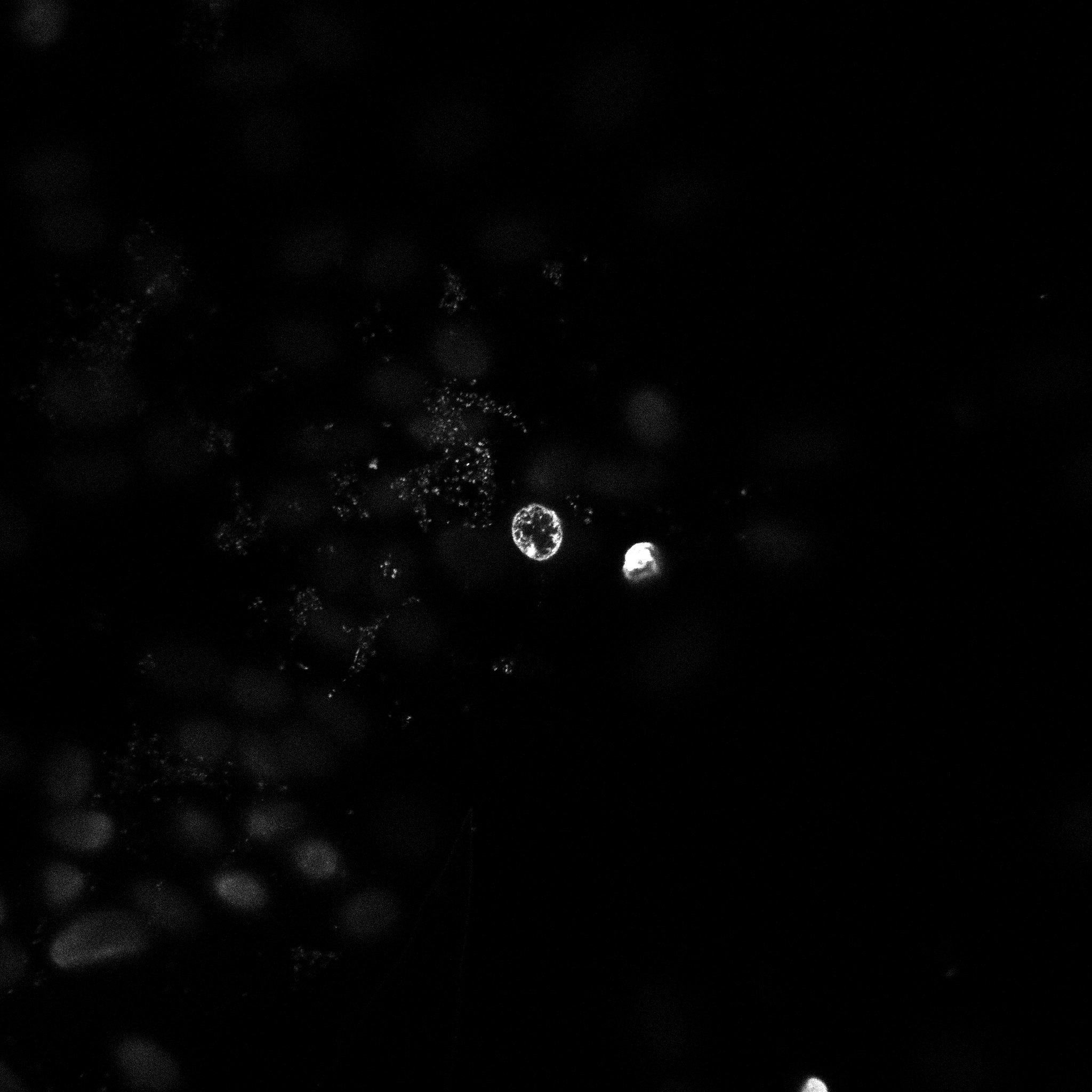

Supplement: Supplementary file 7 — Source Data for Figure 5 [file EMBR-24-e53408-s006.zip › Figure 5/5E/Fig 5E; 60x MAD2-shRNA, Hoechst-signal.tif]

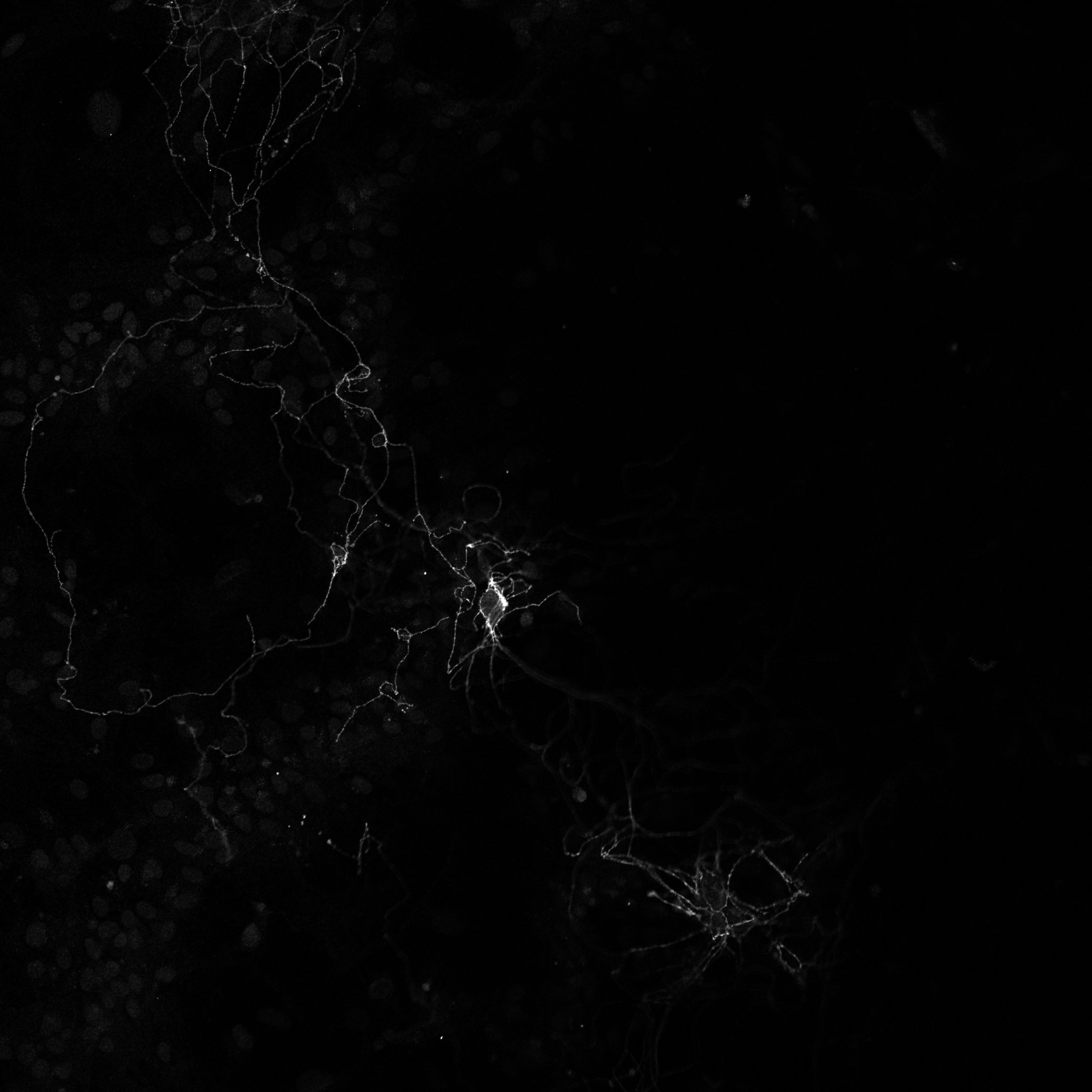

Supplement: Supplementary file 7 — Source Data for Figure 5 [file EMBR-24-e53408-s006.zip › Figure 5/5E/Fig 5E; 20x MAD2-shRNA, SERT-signal.tif]

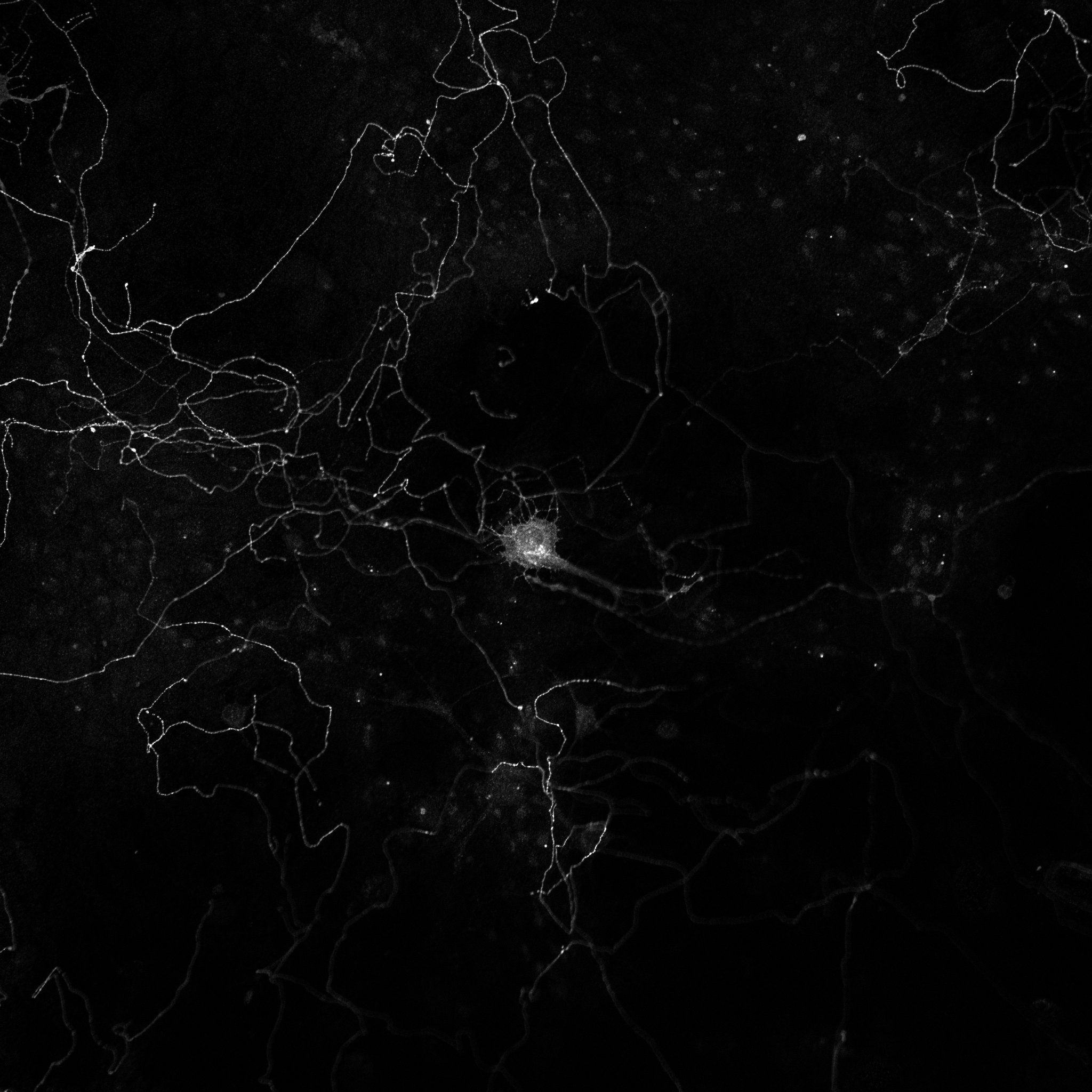

Supplement: Supplementary file 7 — Source Data for Figure 5 [file EMBR-24-e53408-s006.zip › Figure 5/5E/Fig 5E; 20x scramble-shRNA SERT-signal.tif]

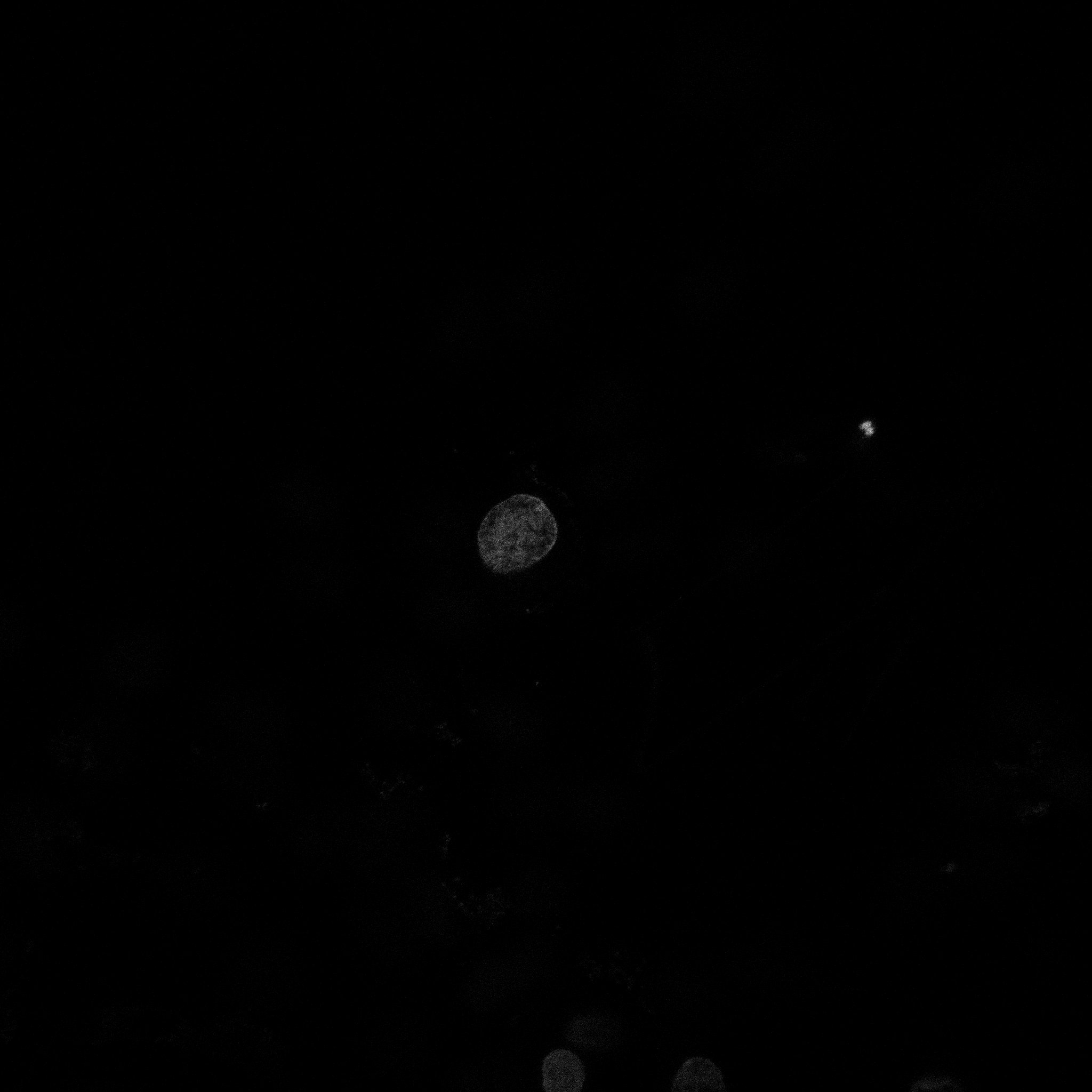

Supplement: Supplementary file 7 — Source Data for Figure 5 [file EMBR-24-e53408-s006.zip › Figure 5/5E/Fig 5E; 60x scramble-shRNA, Hoechst-signal.tif]
