# Supplementary material for: The cell cycle protein MAD2 facilitates endocytosis of the serotonin transporter in the neuronal soma
Source: EMBO Rep. 2023 Aug 2;24(10):e53408. doi: 10.15252/embr.202153408 (PMC10561363; doi:10.15252/embr.202153408)
Supplement: Supplementary file 8 [file EMBR-24-e53408-s001.pdf]

EMBO reports

The cell cycle protein MAD2 facilitates endocytosis of the serotonin transporter in the neuronal soma

Florian Koban and Michael Freissmuth
DOI: 10.15252/embr.202153408

Corresponding author(s): Florian Koban (florian.koban@meduniwien.ac.at)

Review Timeline:

Submission Date:	9th Jun 21
Editorial Decision:	15th Jun 21
Resubmission:	19th Jan 23
Editorial Decision:	10th Mar 23
Revision Received:	26th Apr 23
Editorial Decision:	12th Jun 23
Revision Received:	23rd Jun 23
Accepted:	14th Jul 23

Editor: Martina Rembold

Transaction Report:

(Note: With the exception of the correction of typographical or spelling errors that could be a source of ambiguity, letters and reports are not edited. Depending on transfer agreements, referee reports obtained elsewhere may or may not be included in this compilation. Referee reports are anonymous unless the Referee chooses to sign their reports.)

1st Editorial Decision **15th Jun 2021**

Dear Dr. Koban,

Thank you for the submission of your manuscript to EMBO reports. I have now read and discussed your work with my colleagues here, and I regret to say that we all agree that it is not well suited for our journal.

We appreciate that your study reports that the neurotransmitter transporters SERT, NET and DAT interact with MAD2 via a short motif in their C-terminus. You provide evidence that MAD2 recruits BubR1 and AP2 to induce clathrin-mediated endocytosis of these transporters.

We agree that these are potentially interesting observations that extend earlier data on the role of SAC proteins in the endocytosis of the insulin receptor. However, we also note that the data largely rely on experiments using overexpressed and tagged proteins in HEK293 cells, providing limited insight into the occurrence and function of MAD2-mediated endocytosis in neurons, insight that would be required for potential publication by EMBO reports. As the manuscript stands, we have therefore decided not to proceed with in-depth peer review.

In the interest of your manuscript and your time, I am providing you with an editorial decision on your manuscript that will allow you to submit it elsewhere without further delay. I am sorry to disappoint you on this occasion, and I thank you once more for your interest in our journal.

Yours sincerely,

Martina Rembold, PhD
Senior Editor
EMBO reports

** As a service to authors, EMBO Press provides authors with the ability to transfer a manuscript that one journal cannot offer to publish to another journal, without the author having to upload the manuscript data again. To transfer your manuscript to another EMBO Press journal using this service, please click on
Link Not Available

Dear Dr. Pulverer,

Please find enclosed the manuscript entitled “The mitotic checkpoint protein MAD2 delivers the serotonin transporter to endocytosis“.

The present work is a revised version of our previous submission (EMBOR-2021-53408V1). In brief, our study shows that the cell cycle protein MAD2 mediates endocytic internalization of the neuronal serotonin transporter/SERT. This is the first description of the mechanism, by which a monoamine transporter undergoes endocytosis. Monoamine transporters (and other members of the SLC6 family have long been known to undergo clathrin-mediated endocytosis), but the link to the clathrin coat has remained enigmatic. In addition, our work sheds light on another enigma, i.e. why cell cycle proteins should be present in post-mitotic neurons.

In your previous assessment of our first submission you (or the editorial office) recommended that experiments should be done in primary neurons to substantiate the relevance for endogenously expressed SERT: accordingly, in the current submission, we included a series of experiments on rat primary serotonergic neurons which conclusively confirm the hypothesis that MAD2 promotes native SERT-endocytosis. In addition, we focused on SERT (and omitted data on the transporters for dopamine and norepinephrine). Thus the analysis is more detailed and coherent than in the previous version.

We very much hope that our work is now judged suitable to be reviewed for publication in EMBO reports.

Yours sincerely,

Florian Koban

Dear Florian,

Thank you for providing feedback to the referee reports, which you find again copied below my signature.

As discussed earlier today, we would like to invite you to revise your manuscript along the lines outlined in your point-by-point response. Please clearly describe and discuss that the observed MAD2-dependent endocytosis occurs in the soma of neurons rather than at the synapse. Please also address the remaining referee concerns as outlined in your revision plan.

Please note that acceptance of the manuscript will depend on a positive outcome of a second round of review. It is EMBO reports policy to allow a single round of revision only and acceptance or rejection of the manuscript will therefore depend on the completeness of your responses included in the next, final version of the manuscript.

We realize that it is difficult to revise to a specific deadline. In the interest of protecting the conceptual advance provided by the work, we recommend a revision within 3 months (June 10th). Please discuss the revision progress ahead of this time with the editor if you require more time to complete the revisions.

You can either publish the study as a short report or as a full article. For short reports, the revised manuscript should not exceed 27,000 characters (including spaces but excluding materials & methods and references) and 5 main plus 5 expanded view figures. The results and discussion sections must further be combined, which will help to shorten the manuscript text by eliminating some redundancy that is inevitable when discussing the same experiments twice. For a normal article there are no length limitations, but it should have more than 5 main figures and the results and discussion sections must be separate. In both cases, the entire materials and methods must be included in the main manuscript file.

*****IMPORTANT NOTE:
We perform an initial quality control of all revised manuscripts before re-review. Your manuscript will FAIL this control and the handling will be DELAYED if the following APPLIES:

1) A data availability section providing access to data deposited in public databases is missing. If you have not deposited any data, please add a sentence to the data availability section that explains that.

2) Your manuscript contains statistics and error bars based on n=2. Please use scatter blots in these cases. No statistics should be calculated if n=2.

When submitting your revised manuscript, please carefully review the instructions that follow below. Failure to include requested items will delay the evaluation of your revision.*****

When submitting your revised manuscript, we will require:

1) a .docx formatted version of the manuscript text (including legends for main figures, EV figures and tables). Please make sure that the changes are highlighted to be clearly visible.

2) individual production quality figure files as .eps, .tif, .jpg (one file per figure).
Please download our Figure Preparation Guidelines (figure preparation pdf) from our Author Guidelines pages https://www.embopress.org/page/journal/14693178/authorguide for more info on how to prepare your figures.

3) a .docx formatted letter INCLUDING the reviewers' reports and your detailed point-by-point responses to their comments. As part of the EMBO Press transparent editorial process, the point-by-point response is part of the Review Process File (RPF), which will be published alongside your paper.

4) a complete author checklist, which you can download from our author guidelines (). Please insert information in the checklist that is also reflected in the manuscript. The completed author checklist will also be part of the RPF.

5) Please note that all corresponding authors are required to supply an ORCID ID for their name upon submission of a revised manuscript (). Please find instructions on how to link your ORCID ID to your account in our manuscript tracking system in our Author guidelines
()

6) We replaced Supplementary Information with Expanded View (EV) Figures and Tables that are collapsible/expandable online. A maximum of 5 EV Figures can be typeset. EV Figures should be cited as 'Figure EV1, Figure EV2" etc... in the text and their respective legends should be included in the main text after the legends of regular figures.

- For the figures that you do NOT wish to display as Expanded View figures, they should be bundled together with their legends in a single PDF file called *Appendix*, which should start with a short Table of Content. Appendix figures should be referred to in the main text as: "Appendix Figure S1, Appendix Figure S2" etc. See detailed instructions regarding expanded view here:

- Additional Tables/Datasets should be labeled and referred to as Table EV1, Dataset EV1, etc. Legends have to be provided in a separate tab in case of .xls files. Alternatively, the legend can be supplied as a separate text file (README) and zipped together with the Table/Dataset file.

7) Please note that a Data Availability section at the end of Materials and Methods is now mandatory. In case you have no data that requires deposition in a public database, please state so instead of refereeing to the database.
See also < https://www.embopress.org/page/journal/14693178/authorguide#dataavailability>). Please note that the Data Availability Section is restricted to new primary data that are part of this study.

8) At EMBO Press we ask authors to provide source data for the main figures. Our source data coordinator will contact you to discuss which figure panels we would need source data for and will also provide you with helpful tips on how to upload and organize the files.

Additional information on source data and instruction on how to label the files are available .

9) The journal requires a statement specifying whether or not authors have competing interests (defined as all potential or actual interests that could be perceived to influence the presentation or interpretation of an article). In case of competing interests, this must be specified in your disclosure statement. Further information: https://www.embopress.org/competing-interests

10) Figure legends and data quantification:
The following points must be specified in each figure legend:

- the name of the statistical test used to generate error bars and P values,
- the number (n) of independent experiments (please specify technical or biological replicates) underlying each data point,
- the nature of the bars and error bars (s.d., s.e.m.)

- If the data are obtained from n {less than or equal to} 5, show the individual data points in addition to the SD or SEM.
- If the data are obtained from n {less than or equal to} 2, use scatter blots showing the individual data points.

Discussion of statistical methodology can be reported in the materials and methods section, but figure legends should contain a basic description of n, P and the test applied.

See also the guidelines for figure legend preparation:
https://www.embopress.org/page/journal/14693178/authorguide#figureformat

- Please also include scale bars in all microscopy images.

11) Our journal encourages inclusion of *data citations in the reference list* to directly cite datasets that were re-used and obtained from public databases. Data citations in the article text are distinct from normal bibliographical citations and should directly link to the database records from which the data can be accessed. In the main text, data citations are formatted as follows: "Data ref: Smith et al, 2001" or "Data ref: NCBI Sequence Read Archive PRJNA342805, 2017". In the Reference list, data citations must be labeled with "[DATASET]". A data reference must provide the database name, accession number/identifiers and a resolvable link to the landing page from which the data can be accessed at the end of the reference. Further instructions are available at .

12) As part of the EMBO publication's Transparent Editorial Process, EMBO reports publishes online a Review Process File to accompany accepted manuscripts. This File will be published in conjunction with your paper and will include the referee reports, your point-by-point response and all pertinent correspondence relating to the manuscript.

You are able to opt out of this by letting the editorial office know (emboreports@embo.org). If you do opt out, the Review Process File link will point to the following statement: "No Review Process File is available with this article, as the authors have chosen not to make the review process public in this case."

We would also welcome the submission of cover suggestions, or motifs to be used by our Graphics Illustrator in designing a cover.

I look forward to seeing a revised form of your manuscript when it is ready. Please use this link to submit your revision:
https://embor.msubmit.net/cgi-bin/main.plex

Kind regards,

Martina

Martina Rembold, PhD
Senior Editor
EMBO reports

****

Referee #1:

I have reviewed "The mitotic checkpoint 1 protein MAD2 delivers the serotonin transporter to endocytosis" by Koban and Freismuth. The authors first make the provocative observation that the C-termini of SLC6 transporters appear to include MAD2-interaction motifs (MIMs) and, importantly, that this motif in DAT resides within a region previously shown to be required for endocytosis. The co-localization of MAD2 and TPH to some of the same cells in the raphe (Fig 1b) is convincing. The confocal images showing that some MAD2 puncta are near the cell surface is reasonable (Fig 1d), but not that useful without a co-label for SERT. The absence of this co-label is not really of concern, since GST pull downs show an interaction between MAD2 and SERT in Fig 2. Ablating the interaction with a mutant strengthens this finding. Moreover, further experiments show that a specific conformation appears to be required for the interaction, reenforcing the idea that binding occurs via a mechanism previously established for other proteins. Since other MAD2 binding proteins exist as part of a complex with BubR1 and p31comet, the finding that both are expressed in raphe neurons (Fig 3) supports the idea that SERT and MAD2 are involved in a similar complex. Co-IP assays using heterologously expressed cells (Fig 3C) lend further support to this idea. However, GAT and NET appear to bind MAD2 to a lesser extent that DAT and SERT and this should be briefly discussed (see minor concerns). Disruption of at least some elements of the complex with siRNA to SERT in Fig 3 D-G is a nice idea. The lack of a significant effect for comet is not really concerning - these are tough experiments. The experiments in Fig 4 showing that MAD2 knock down alters the cell surface localization and the Vmax of SERT significantly elevates the impact of the paper since it demonstrates that the interaction of the MAD2 complex with SERT is functionally significant. The decrease in co-localization to rab5 and 7 is a nice addition. It is impressive that the authors extended their findings to cultured neurons in Fig 5 and the data from serotonergic cells are convincingly presented. Indeed, Fig 5E is arguably the most important piece of data in the paper.

Concerns and overall evaluation
The Co-IP results for GAT and NET in Fig 3C seem to show lower binding of MAD2 and adaptin compared to SERT and NET. Comet binding to NET may also be less robust binding to Hsp70 is higher. These differences should be briefly discussed, possibly in relation to differences in the MAD2 binding site shown in Fig EV1.

The data shown in EV3 and 4 are pretty and seem to represent a significant amount of work. However, the relative localization to endocytic rather than secretory pathway compartments is a relatively soft call, and the conclusion about SERT trafficking should be softened.

The data using the serotonergic neurons in 5E, F seems to negate the need for the data shown in 5A,B, but this, too is a minor concern.

The rest of paper is both convincing and important. It addresses a major issue in the function of SLC6 neurotransmitter transporter that has remain unresolved and will be of interest to every lab that studies these proteins in animal models. The data are also translationally relevant since SLC6 transporters are the targets of a widely used class of pharmaceuticals. This point, and the idea that MAD2 complex components could represent novel drug targets might be mentioned in the discussion to broaden the potential readership of the paper. On the other hand, this remains highly speculative and might distract from the elegance of the basic science.

Typos and stylistic points
At the end of the introduction, consider substituting a different word (or phrase) for "preclude" since MAD2 reduced, but did not eliminate endocytosis.

Results line 199 consider, substitute "By contrast" for "Whereas"

Rewrite Fig legend EVIC substituting "with or without as indicated" for "omitting"

Consider rephrasing the sentence "Simplified, two types of endocytosis can be distinguished, i.e. clathrin-dependent and -independent endocytosis."

Referee #2:

The present study by F. Koban and M. Freissmuth describes a new molecular mechanism for the recruitment of serotonin-transporters (SERT) to endocytosis. The authors propose that a specialized MAD2-interaction motif (MIM) in the C-terminal domain of SERT recruits MAD2 (as well as BubR1 and p31comet) and subsequently AP-2 for clathrin-mediated endocytosis. Additionally they provide insights into the role of spindle assembly checkpoint proteins (SAC) in non-dividing tissue such as the brain. The evidence for this was collected by a variety of biochemical analysis, confocal imaging and siRNA-mediated knockdown of MAD2 in cell cultures of HEK-293 and cultured neurons of the dorsal raphe nucleus. By making use of these techniques, the authors provide the following evidence: i) MAD2, BubR1 and p31comet are co-expressed in cell cultures of neurons of the dorsal raphe nucleus. ii) MAD2 interacts with the C-Terminus of SERT measured by using a GST-pull down assay in HEK-293 cells. iii) SERT, MAD2, BubR1 and p31comet form a complex as revealed by immunoprecipitation from HEK-293 cells. iv) Internalization of SERT, measured by enzymatic assays and confocal imaging is dependent on MAD2 in HEK-293 cells and in cultured neurons of the dorsal raphe nucleus. v) Subcellular distribution of SERT is MAD2-dependend: siRNA-mediated knockdown of MAD2 increases the amount of SERT at the surface of the cell membrane and in parallel by reducing the amount of endosome-localized SERT.
A particular strength of the present study is the very detailed biochemical analysis of the interaction of SERT and MAD2 not only in HEK-293 cells but also in the physiologically more relevant model system of cultures neurons of the dorsal raphe nucleus. Compelling evidence is provided by the fact that knockdown of MAD2 decreases internalization of SERT. However, we have the following concerns.

The studied mechanisms have been investigated exclusively at the soma and not directly at synapses. However, the mechanisms of endocytosis at synapses might be different from those occurring at the soma. The authors could validate their conclusions by measuring endocytosis at synapses. Alternatively, we suggest to prominently mention the caveat that the conclusions might not apply to synapses.

Line 72: Non-clathrin-dependend mechanisms for endocytosis of dopamine transporters (DAT) have been described (https://www.nature.com/articles/nn.2781). The authors could discuss the role of non-clathrin-dependend mechanisms for endocytosis of serotonin transporters.

Line 318 - 323: The argument is not completely clear to us. Please rephrase these two sentences.

Referee #3:

Summary:
1. Does this manuscript report a single key finding? YES
The paper describes a novel mechanism of internalization of the serotonin transporter.

2. Is the reported work of significance (YES), or does it describe a confirmatory finding or one that has already been documented using other methods or in other organisms etc (NO)? YES

3. Is it of general interest to the molecular biology community? YES
The mechanisms of internalization of monoamine transporters are poorly understood and are key to neuronal functions.

4. Is the single major finding robustly documented using independent lines of experimental evidence (YES), or is it really just a preliminary report requiring significant further data to become convincing, and thus more suited to a longer format article (NO)? NO

The paper by Koban and Freissmuth describes a novel mechanism of internalization of the serotonin transporter (SERT). The authors find that MAD2, a small spindle assembly checkpoint protein is expressed in postmitotic neurons. Co-immunoprecipitation experiments carried out in HEK cells identified a biochemical interaction between MAD2 and SERT that was proposed from sequence analysis. Co-IPs also revealed the participation in the interaction of AP2, the clathrin adaptor complex. This finding led the authors to find a co-localization of SERT with endosomal markers. Decrease of MAD2 levels using RNAi increased surface levels of SERT in cultured dorsal raphe neurons, which is interpreted as an indication of the relevance of MAD2 in SERT endocytosis.
The manuscript combines biochemistry and cell biology to shed light on the mechanisms of monoamine receptor internalization. Figures 1,2 and 3 suggest the presence of a biologically relevant interaction based on overexpression strategies in a cell line but experiments using neurons do not reveal if a MAD2/AP2/SERT interaction is indeed involved in the trafficking of the transporter. The quality of the confocal images presented, and the associated analysis is below the necessary standards to provide a mechanistic insight. The functional implications of the described biochemical interaction requires the use of a complete different range of techniques, such as live imaging of SERT to visualize in real-time the trafficking of the protein and/or optical microscopy

of improved resolution for doing co-localization experiments. These sorts of experiments require a considerable amount of time and effort that will substantially change the structure of the paper but, are completely necessary to validate biochemical findings.

Major points:

1. The quality of images is below the necessary standards to sustain the claims of the paper. For example, Fig. 1D shows a staining of MAD2 that should provide a clear description of the distribution of the protein but, I found difficult to interpret. The image shows a somatic region and a neurite. A thorough description of MAD2 distribution is necessary. For example, co-staining with axonal, synaptic or dendritic markers is required. What are these punctate structures (line 156)? Is MAD2 concentrated in the Golgi? Should MAD2 be present in the nucleus? These aspects should be clarified to backup an interaction with SERT. The presentation of the images must improve. For example, the line profile in Fig. 1D should be expressed in micrometers. Image resolution must be increased.
2. Figure 4. The quantification of SERT present in the membrane and cytosol is not convincing. HEK cells are not the best experimental platform to carry out this type of analysis. Membrane fluorescent signals appear to be saturated. If this is the case, the quantification is compromised. Data shown in Figure 4 is not convincing. Alternative strategies can be used, for example, change to a different cell line or even better, use cultured neurons. All imaging experiments should be carried out in neurons.
3. Figures EV3 and EV4 are not convincing. They are obtained in somatic regions. Images taken at the level of synapses, or at least in dendritic/axonal locations are required. The authors cannot bring to discussion a possible role of the proposed interaction in the AIS (lines 420-427) if they do not image this neuronal compartment.
4. Images do not backup the interaction of MAD2 and SERT with AP2. I would like to see an image revealing the co-localization of these three proteins in a neuronal compartment.
5. Trafficking of SERT. Fig.5E should be carried out using live imaging. For example, looking at the recycling of SERT-phluorin with endogenous and decreased levels of MAD2. This could be an important experiment to demonstrate that the proposed interaction is relevant to the internalization of SERT.

Minor comments:
1. Presence of MAD2 in glial cells. Lines 317-322 identifies MAD2 is in glial cells, however, line 387 makes an opposite statement based on the literature "MAD2 was present in neurons but not in glial cells". Please clarify.
2. Figure legends of images: uM to um.
3. Fig. EV2. Line fits should begin at 0,0. The offset in the y axes cannot be interpreted.

Point-by-point reply:

We thank the referees for their positive comments and for their constructive criticism. We address the points, which were raised, as follows:

Referee #1:

I have reviewed "The mitotic checkpoint 1 protein MAD2 delivers the serotonin transporter to endocytosis" by Koban and Freismuth. The authors first make the provocative observation that the C-termini of SLC6 transporters appear to include MAD2-interaction motifs (MIMs) and, importantly, that this motif in DAT resides within a region previously shown to be required for endocytosis. The co-localization of MAD2 and TPH to some of the same cells in the raphe (Fig 1b) is convincing. The confocal images showing that some MAD2 puncta are near the cell surface is reasonable (Fig 1d), but not that useful without a co-label for SERT. The absence of this co-label is not really of concern, since GST pull downs show an interaction between MAD2 and SERT in Fig 2. Ablating the interaction with a mutant strengthens this finding. Moreover, further experiments show that a specific conformation appears to be required for the interaction, reenforcing the idea that binding occurs via a mechanism previously established for other proteins. Since other MAD2 binding proteins exist as part of a complex with BubR1 and p31comet, the finding that both are expressed in raphe neurons (Fig 3) supports the idea that SERT and MAD2 are involved in a similar complex. Co-IP assays using heterologously expressed cells (Fig 3C) lend further support to this idea. However, GAT and NET appear to bind MAD2 to a lesser extent that DAT and SERT and this should be briefly discussed (see minor concerns). Disruption of at least some elements of the complex with siRNA to SERT in Fig 3 D-G is a nice idea. The lack of a significant effect for comet is not really concerning - these are tough experiments. The experiments in Fig 4 showing that MAD2 knock down alters the cell surface localization and the Vmax of SERT significantly elevates the impact of the paper since it demonstrates that the interaction of the MAD2 complex with SERT is functionally significant. The decrease in co-localization to rab5 and 7 is a nice addition. It is impressive that the authors extended their findings to cultured neurons in Fig 5 and the data from serotonergic cells are convincingly presented. Indeed, Fig 5E is arguably the most important piece of data in the paper.

Concerns and overall evaluation
The Co-IP results for GAT and NET in Fig 3C seem to show lower binding of MAD2 and adaptin compared to SERT and NET. Comet binding to NET may also be less robust binding to Hsp70 is higher. These differences should be briefly discussed, possibly in relation to differences in the MAD2 binding site shown in Fig EV1.

Reply:
We should like to point out that the correlations shown in Fig. EV2 are based on the systematic quantification of the proteins (i.e., MAD2, p31Comet, □-adaptin, HSP70) co-immunoprecipitated in complex with individual transporters (GAT-1, NET, DAT, SERT). Hence, the differences are in fact the very basis of the correlations in Fig. EV2.
We can also provide a rational explanation for the higher levels of HSP70, which were retrieved with NET: Firstly, the levels of HSP70 reflect the amount of nascent (ER-resident immature) transporter, which is clearly highest in NET. Secondly, the pertinent site in the C-terminus of NET interacts with HSP70 with higher apparent affinity than that of DAT and SERT: this statement is based on GST pull-down experiments, which are not shown in the manuscript, but provided below for the referee's perusal.

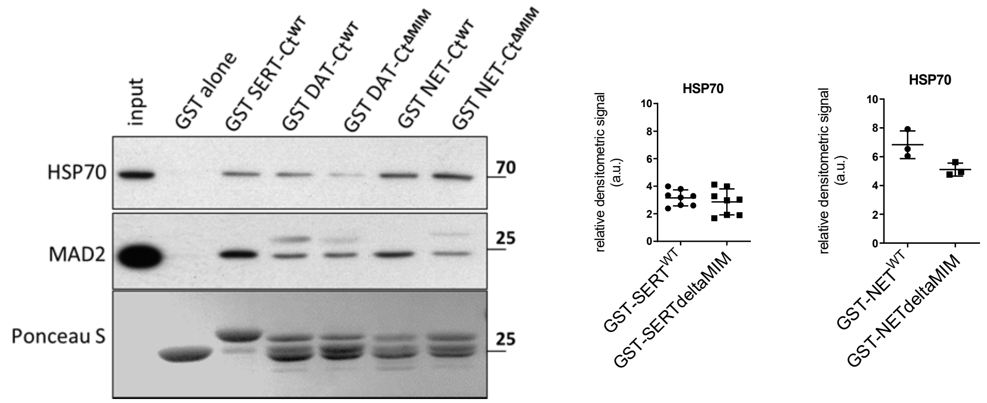

Referee response Fig. 1: Glutathione S-transferase- (GST-)tagged transporter C-termini and deltaMIM mutants were purified and used in GST-protein based interaction assays as outlined under "Materials and Methods" in the main manuscript.

As the referee points out, variations in the MAD2 binding sequence may account – in part – for the differences in MAD2 interaction. In fact, GAT1 harbors a glutamine at a position which is occupied by a hydrophobic residue in conventional MAD2 interaction motifs (Fig. EV1A). The putative MIM of NET and DAT appear similar, but the transporters differ substantially in MAD2 binding: DAT co-immunoprecipitates more MAD2 than NET, whereas the GST-pull-down assay shown above would suggest otherwise. Hence, the MIM-sequence in the transporters per se obviously does not suffice to explain the actual quantity of MAD2-association in a cellular system. However, we should like to refrain from expanding on the differences in MAD2 binding and MIMs for space reasons and because we would very much prefer to base a discussion on a more detailed systematic investigation of the binding-kinetics between MAD2 and transporter MIMs.

The data shown in EV3 and 4 are pretty and seem to represent a significant amount of work. However, the relative localization to endocytic rather than secretory pathway compartments is a relatively soft call, and the conclusion about SERT trafficking should be softened.

Reply:
We are grateful for the referee's laudatory comments. We also agree with the referee's point. The respective sentence now reads (on. p. 12, lines 315-318):
"Taken together, these data show that, in rat dorsal raphe neurons, a substantial fraction of intracellular SERT resides in endocytic compartments (Fig. 5B), which support recycling rather than degradation."

Instead of:

"Importantly, the findings suggest that, in rat dorsal raphe neurons, the bulk of intracellular accumulating SERT is not derived from the secretory pathway but from endocytic compartments (Fig. 5D), which presumably mediate SERT recycling rather than degradation."

*We would also like to point out that, in the meantime, we further substantiated our findings by additional experiments and a statistical evaluation of our co-localization study is included as **new Fig. 5B**.*

The data using the serotonergic neurons in 5E, F seems to negate the need for the data shown in 5A,B, but this, too is a minor concern.

Reply:
We agree that Fig. 5E and F suffice to show the reduction in neuronal MAD2 levels resulting from virally mediated knock-down of MAD2. However, we believe that the data in new Fig. 5C and D (former Fig. 5A and B) are nevertheless valuable, because they provide an independent confirmation (by PCR and immunoblotting), which also allows for quantifying the efficiency of the gene knock-down.

The rest of paper is both convincing and important. It addresses a major issue in the function of SLC6 neurotransmitter transporter that has remain unresolved and will be of interest to every lab that studies these proteins in animal models. The data are also translationally relevant since SLC6 transporters are the targets of a widely used class of pharmaceuticals. This point, and the idea that MAD2 complex components could represent novel drug targets might be mentioned in the discussion to broaden the potential readership of the paper. On the other hand, this remains highly speculative and might distract from the elegance of the basic science.

Reply:
We again thank the referee for appreciating our work. We refrained from speculating on targeting the interface in MAD2 complex components because of the space limit (the current version of the manuscript just meets the space limit of 27,000 characters).

Typos and stylistic points
At the end of the introduction, consider substituting a different word (or phrase) for "preclude" since MAD2 reduced, but did not eliminate endocytosis.

Reply:
Corrected as suggested
The sentence now reads (lines 104-105): "Accordingly, lentivirus-mediated depletion of MAD2 in cultured serotonergic rat neurons reduced SERT endocytosis."

Results line 199 consider, substitute "By contrast" for "Whereas"
Reply:
Corrected as suggested (line 188)

Rewrite Fig legend EVIC substituting "with or without as indicated" for "omitting"
Reply:
Corrected as suggested (lines 957-958)

Consider rephrasing the sentence "Simplified, two types of endocytosis can be distinguished, i.e. clathrin-dependent and -independent endocytosis."

Reply:
The sentence was deleted in order to meet the space limit.

Referee #2:

The present study by F. Koban and M. Freissmuth describes a new molecular mechanism for the

recruitment of serotonin-transporters (SERT) to endocytosis. The authors propose that a specialized MAD2-interaction motif (MIM) in the C-terminal domain of SERT recruits MAD2 (as well as BubR1 and p31comet) and subsequently AP-2 for clathrin-mediated endocytosis. Additionally they provide insights into the role of spindle assembly checkpoint proteins (SAC) in non-dividing tissue such as the brain. The evidence for this was collected by a variety of biochemical analysis, confocal imaging and siRNA-mediated knockdown of MAD2 in cell cultures of HEK-293 and cultured neurons of the dorsal raphe nucleus. By making use of these techniques, the authors provide the following evidence: i) MAD2, BubR1 and p31comet are co-expressed in cell cultures of neurons of the dorsal raphe nucleus. ii) MAD2 interacts with the C-Terminus of SERT measured by using a GST-pull down assay in HEK-293 cells. iii) SERT, MAD2, BubR1 and p31comet form a complex as revealed by immunoprecipitation from HEK-293 cells. iv) Internalization of SERT, measured by enzymatic assays and confocal imaging is dependent on MAD2 in HEK-293 cells and in cultured neurons of the dorsal raphe nucleus. v) Subcellular distribution of SERT is MAD2-dependend: siRNA-mediated knockdown of MAD2 increases the amount of SERT at the surface of the cell membrane and in parallel by reducing the amount of endosome-localized SERT.
A particular strength of the present study is the very detailed biochemical analysis of the interaction of SERT and MAD2 not only in HEK-293 cells but also in the physiologically more relevant model system of cultures neurons of the dorsal raphe nucleus. Compelling evidence is provided by the fact that knockdown of MAD2 decreases internalization of SERT. However, we have the following concerns.

The studied mechanisms have been investigated exclusively at the soma and not directly at synapses. However, the mechanisms of endocytosis at synapses might be different from those occurring at the soma. The authors could validate their conclusions by measuring endocytosis at synapses. Alternatively, we suggest to prominently mention the caveat that the conclusions might not apply to synapses.

Reply:
The same point was raised by referee #3. Hence, here we reply to the criticism of both, referee #2 and referee #3. We consider this an important point and we are grateful for the opportunity to address it as follows:
The "business end" of SERT is to be delivered to the synaptic boutons, to accomplish its eponymous action (i.e., the retrieval of released serotonin). Thus, we understand that regulation of SERT at the presynaptic specializations ought to be of interest. However, it has long been known that neurotransmitter transporters of the SLC6 family (GAT1, DAT, NET, SERT, GlyT2) are also abundantly present in the neuronal soma. Here we focus on SERT in the neuronal soma for reasons outlined below. We did not intend to convey the impression that, in our experiments, the somatic membrane merely served as a "surrogate-compartment" in order to learn about the mechanisms of SERT endocytosis at the synapse. Several observations led us to expect that it is rather the neuronal soma at which MAD2-mediated endocytosis of SERT occurres. (i) As described by us (this study) and others (e. g., https://doi.org/10.1016/j.neuint.2008.12.004) the bulk of somatic SERT is intracellular. This is not only true in cultured rat dorsal raphe neurons but also in situ: we identified serotonergic neurons in slices prepared from the dorsal raphe of murine brain by staining for tryptophane hydroxylase (TPH; cf. top row in "Referee response Fig. 2" inserted below for the referee's perusal). The bulk of SERT immunoreactivity was visualized within the cytosol (second and third row in "Referee response Fig. 2"). MAD2 was also present in these neurons; finally, there was a modest extent of co-localization between MAD2 and SERT in some regions (fourth row in "Referee response Fig. 2").

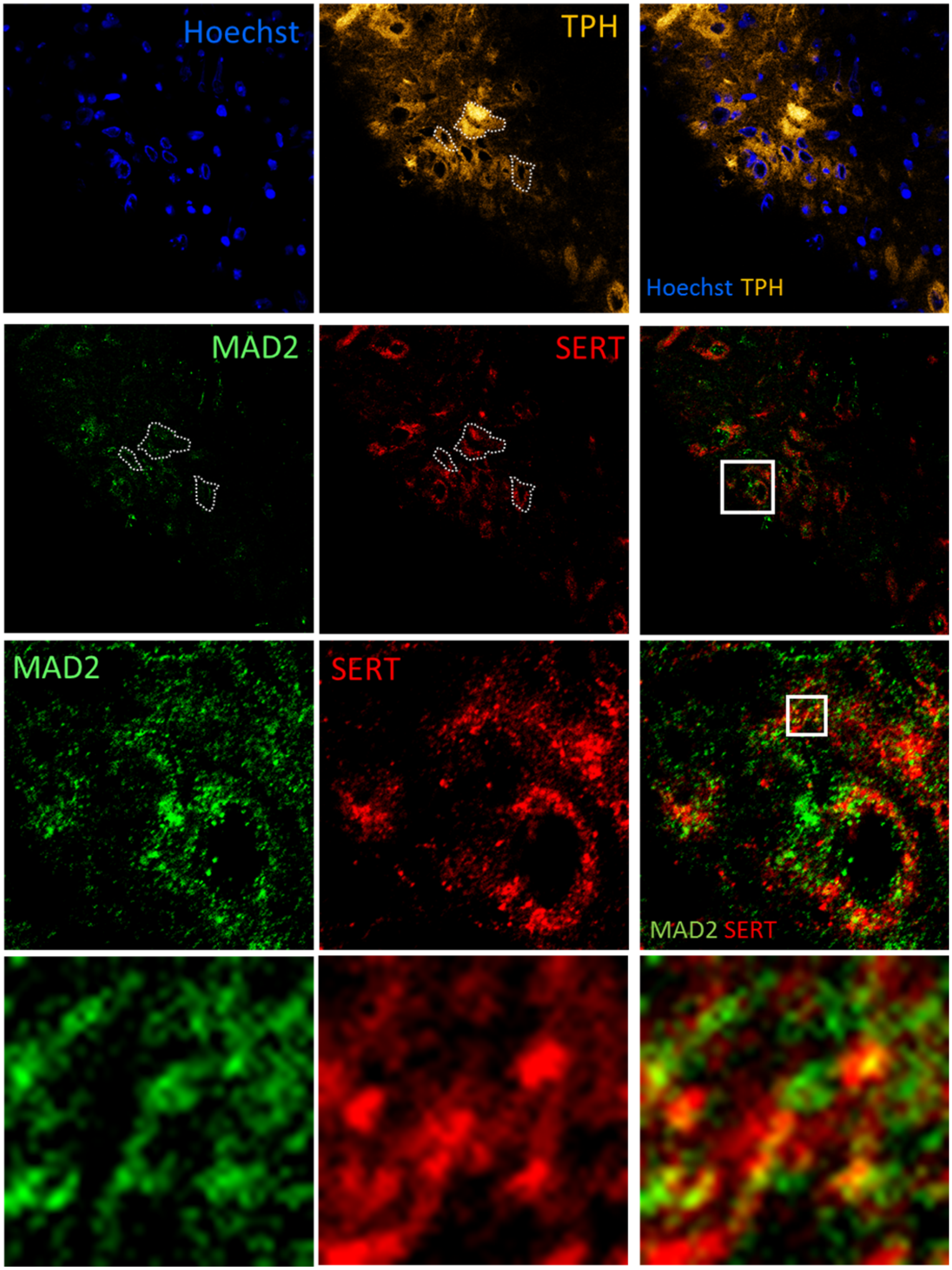

Referee response Fig. 2: Cryosections of the brain region containing the mouse dorsal raphe nucleus were fixed in acetone/methanol (1:1) and subjected to immunofluorescence staining using the indicated primary and appropriate secondary antibodies. Images were captured by confocal microscopy at a magnification of 20-fold and 60-fold for the first two and the third row, respectively. White boxes indicate the magnified area in the panel below. The right-hand column shows the overlay of the images displayed on the left-hand and in the middle column.

The fact that SERT co-localizes with endocytic markers like Rab11A or Rab7A (Fig. EV5 and new Fig. 5B) indicates that SERT endocytosis in the somatic compartment is of distinct biological significance.
*We included additional data (in new Fig. EV3), which demonstrate that endocytic compartments (Rab7+ late endosomes, Rab11+ recycling endosomes) are enriched within the soma of serotonergic neurons, whereas neurite extensions of serotonergic are virtually devoid of these compartments. In other studies (e. g., 10.1523/JNEUROSCI.1391-15.2015) very similar results were obtained for mouse brain dopaminergic neurons, in which endosomes and lysosomes were concentrated mostly within their cell bodies in the midbrain and virtually absent from their (striatal) axons. (iii) The distribution of MAD2 within serotonergic neurons corresponds to the distribution of endocytic markers, i. e. MAD2 is confined to the soma of serotonergic neurons and absent from the neurite extensions (**new Fig. EV3**)*

In addition, to the best of our knowledge, there is no published study, which has hitherto described SERT internalization at the synapse (or in the axonal compartment).

Hence, in our revised manuscript we followed up on the recommendation of referee #2 "to prominently mention the caveat that the conclusions might not apply to synapses."

The following changes were made:

The following 2 paragraphs were inserted into the section "Results and Discussion":

(i) p. 10/11, lines 296-304:
"Next, we examined the role of MAD2 in endocytosis of endogenous SERT in cultured rat dorsal raphe neurons. Two lines of evidence indicated that only the neuronal soma qualified as the putative compartment of MAD2-mediated SERT endocytosis: (i) immunofluorescence staining demonstrated that, in cultured serotonergic neurons, the endosomal markers Rab7A and Rab11A were conspicuously concentrated in the soma but virtually undetectable in serotonergic neurite extensions (presumably representing axonal arborizations and dendrites) (Fig. EV3A and B). (ii) MAD2 was also confined to the soma (Fig. EV3C). Hence, in subsequent experiments, we focused on SERT trafficking in the neuronal soma."

(ii) on p. 13, lines 344-356:
"SERT (and all related neurotransmitter transporters) exert their eponymous action, which is the retrieval of released neurotransmitter, in the presynaptic specialization. Recent evidence indicates that DAT undergoes regulated endocytic recycling in synaptic boutons (Kearney et al., 2023). However, it remains to be shown that presynaptic SERT does undergo endocytosis. If this is the case, internalization is very likely to be independent of MAD2, because all neurite extension and thus the axonal compartment were devoid of MAD2 (Fig. EV3). Hence, alternative pathways are likely involved. Previously, flotillin-1 (Flot1) was shown to support regulated endocytosis of DAT required for endocytosis (Cremona et al, 2011). SERT also interacts with Flot1 (Reisinger et al, 2019). Flot1-mediated endocytosis is clathrin-independent (Glebov et al, 2006) (and thus presumably AP2/MAD2-independent). It is therefore conceivable that regulated endocytosis of presynaptic SERT also occurs via a Flot1-dependent mechanism."

The subtitle of the subsection, where these two paragraphs were included, was changed from:
"Endocytosis of SERT in cultured neurons requires MAD2"
to
"MAD2 facilitates SERT endocytosis in the soma of serotonergic neurons." (p. 10, line 295)

The title of the paper was changed from:
“The mitotic checkpoint protein MAD2 delivers the serotonin transporter to endocytosis”
to
“The cell cycle protein MAD2 facilitates endocytosis of the serotonin transporters in the neuronal soma.”

We hope that these changes (and the additional minor changes throughout the text) appropriately underscore the neuronal soma as the focus of the present study. We also hope that our wording is adequate to stress that other mechanisms are likely to support the (currently still putative) endocytosis of SERT in the presynaptic specialization.

Line 72: Non-clathrin-dependend mechanisms for endocytosis of dopamine transporters (DAT) have been described (https://www.nature.com/articles/nn.2781). The authors could discuss the role of non-clathrin-dependend mechanisms for endocytosis of serotonin transporters.

Reply:
This point was addressed in our response to the previous point (please see above).

Line 318 - 323: The argument is not completely clear to us. Please rephrase these two sentences.

Reply:
The sentences were reworded to explain that the shRNA-induced drop in neuronal MAD2 mRNA and protein levels cannot be quantified by extracting RNA and protein from mass cultures, because neurons must be cultured on a glial layer. The reworded text now reads (p. 11, lines 321-328):
“The suitability of this knock-down approach was first verified in a culture of pure rat cortical glial cells: depletion of the target gene product was documented by quantitative PCR and immunoblotting, which demonstrated a substantial reduction in MAD2 mRNA (Fig. 5C) and protein (Fig. 5D) five days after infection. We stress that high amounts of glial cells are present in and required for neuronal cultures. Hence, at the level of the mass culture, it is not possible to quantify the effect of lentivirally encoded shRNA on the bulk level of neuronal MAD2.”

Referee #3:

Summary:
1. Does this manuscript report a single key finding? YES
The paper describes a novel mechanism of internalization of the serotonin transporter.

2. Is the reported work of significance (YES), or does it describe a confirmatory finding or one that has already been documented using other methods or in other organisms etc (NO)? YES

3. Is it of general interest to the molecular biology community? YES
The mechanisms of internalization of monoamine transporters are poorly understood and are key to neuronal functions.

4. Is the single major finding robustly documented using independent lines of experimental evidence (YES), or is it really just a preliminary report requiring significant further data to become convincing, and thus more suited to a longer format article (NO)? NO

The paper by Koban and Freissmuth describes a novel mechanism of internalization of the serotonin transporter (SERT). The authors find that MAD2, a small spindle assembly checkpoint protein is expressed in postmitotic neurons. Co-immunoprecipitation experiments carried out in HEK cells

identified a biochemical interaction between MAD2 and SERT that was proposed from sequence analysis. Co-IPs also revealed the participation in the interaction of AP2, the clathrin adaptor complex. This finding led the authors to find a co-localization of SERT with endosomal markers. Decrease of MAD2 levels using RNAi increased surface levels of SERT in cultured dorsal raphe neurons, which is interpreted as an indication of the relevance of MAD2 in SERT endocytosis.
The manuscript combines biochemistry and cell biology to shed light on the mechanisms of monoamine receptor internalization. Figures 1,2 and 3 suggest the presence of a biologically relevant interaction based on overexpression strategies in a cell line but experiments using neurons do not reveal if a MAD2/AP2/SERT interaction is indeed involved in the trafficking of the transporter. The quality of the confocal images presented, and the associated analysis is below the necessary standards to provide a mechanistic insight. The functional implications of the described biochemical interaction requires the use of a complete different range of techniques, such as live imaging of SERT to visualize in real-time the trafficking of the protein and/or optical microscopy of improved resolution for doing co-localization experiments. These sorts of experiments require a considerable amount of time and effort that will substantially change the structure of the paper but, are completely necessary to validate biochemical findings.

Reply:
The goal of the present study was to provide evidence for the general requirement of MAD2 during endocytosis of the serotonin transporter. We agree that a detailed elaboration of the mechanism of all discovered interaction partners (MAD2/p31comet/BubR1/AP2) for SERT endocytosis would be highly desirable. However, we again wish to emphasize that the scope of the current work is rather fundamental than mechanistic as it includes: i) a detailed biochemical characterization of MAD2 binding to SERT (including the binding site on SERT and the required MAD2-conformation); ii) the discovery of mitotic checkpoint proteins in post-mitotic raphe neurons and iii) the evidence for the relevance of MAD2 for SERT endocytosis in HEK-293 cells and in primary neurons.
We agree that live imaging provides information in real-time but, in our opinion, this addresses kinetic issues, which are beyond the scope of the current paper.

*In addition, we also like to draw the referee's attention to an important technical obstacle. We detected MAD2 (together with the main endocytic compartments) exclusively within the soma of serotonergic neurons, but not in the neurite extensions; a pertinent additional figure (**new Fig. EV3**) has been included in the revised version of the manuscript. As shown in Fig. EV4 (formerly EV3) and EV5 (formerly EV4) the somatic membrane of serotonergic neurons is essentially devoid of SERT, presumably because SERT, which reaches the somatic membrane, is rapidly internalized. Thus, the dwell time of SERT on the surface of the neuronal soma is short. Accordingly, finding complexes between SERT/MAD2/AP2 in serotonergic neurons would also be extremely challenging from a stochastic point of view. Thus, even the entire absence of visualizable SERT/MAD2/AP2-puncta at the plasma membrane of cultured dorsal raphe neurons does not diminish the findings and conclusions of our study.*

Major points:

1. The quality of images is below the necessary standards to sustain the claims of the paper. For example, Fig. 1D shows a staining of MAD2 that should provide a clear description of the distribution of the protein but, I found difficult to interpret. The image shows a somatic region and a neurite. A thorough description of MAD2 distribution is necessary. For example, co-staining with axonal, synaptic or dendritic markers is required. What are these punctate structures (line 156)? Is MAD2 concentrated in the Golgi? Should MAD2 be present in the nucleus? These aspects should be clarified to backup an interaction with SERT.
The presentation of the images must improve. For example, the line profile in Fig. 1D should be expressed in micrometers. Image resolution must be increased.

Reply:
(i) Fig. 1D shows that (i) MAD2 is present in the neuronal soma of a TPH-positive (i.e., serotonergic) neuron and (ii) that a fraction of MAD2 is found in apposition to the membrane (this latter point is the rationale for showing the images). Since the image derives from a cryosection of mouse brain and not a neuronal culture it is not possible to visualize the soma and the corresponding (distant) neurite extensions together. As an alternative, we included ***(new) Fig. EV3*** *to show that MAD2 is confined to the neuronal soma. Distant neurite extensions containing SERT (and thus presumably reflecting axonal arborizations and dendrites) are devoid of MAD2 (and of the endocytic markers Rab7A and RAB11A). Hence, in our opinion, a distinction between axons and dendrites is not required.*
We do not understand why MAD2 should concentrate at the Golgi. MAD2 is a protein of 23 kDa, which is likely to freely diffuse into the nucleus. Furthermore, MAD2 does interact with Tpr, a protein located at the nuclear pore complex (10.1101/gad.1677208). Hence the presence of MAD2 within the nucleus is predictable rather than a source of concern.
A pertinent sentence was included in the manuscript (on p. 6, lines 147-149):
"As expected, a fraction of MAD2 was located in the nucleus; this may occur by passive diffusion through the nuclear pore complex (NPC) and/or by association with the NPC-protein Tpr (Lee et al, 2008)."

(ii) We changed the x-axis in the line-profile from pixels to micrometers. We also decided to use an average projection rather than a single image of the Z-stack and to change the colors.

2. Figure 4. The quantification of SERT present in the membrane and cytosol is not convincing. HEK cells are not the best experimental platform to carry out this type of analysis. Membrane fluorescent signals appear to be saturated. If this is the case, the quantification is compromised. Data shown in Figure 4 is not convincing. Alternative strategies can be used, for example, change to a different cell line or even better, use cultured neurons. All imaging experiments should be carried out in neurons.

Reply:
(i) We should like to point out that the level of "membrane fluorescent signals" is immaterial to the experiment: this membrane signal was not quantified. Only the intracellular SERT signal was quantified and this signal was normalized to the intracellular area, over which the signal was captured. Importantly, MAD2 knockdown significantly decreased the intracellular SERT pool in absolute terms.
(ii) We do not understand, why a transformed cell line other than HEK-293 would be superior. In fact, a neuronal origin of HEK-293 was even discussed in the past (10.1096/fj.01-0995fje).
(iii) The effect of MAD2 knockdown (on SERT distribution) is recapitulated in neurons. These data are shown in Fig. 5.

3. Figures EV3 and EV4 are not convincing. They are obtained in somatic regions. Images taken at the level of synapses, or at least in dendritic/axonal locations are required. The authors cannot bring to discussion a possible role of the proposed interaction in the AIS (lines 420-427) if they do not image this neuronal compartment.

Reply:
(i) We addressed the first part of the criticism in our reply to the first point of referee #2 (please see above)

(ii) Concerning the proposed interaction in the AIS:
The wording of the pertinent sentences was changed; it does not refer to the term axonal initial segment (AIS) anymore but refers to the problem of sorting and rerouting of membrane proteins in general terms (on p. 12/13, lines 364-370).

“In fact, a recent study describes endocytosis as a key mechanism for the maintenance of neuronal polarity (Eichel et al, 2022): axonal surface proteins, which aberrantly diffuse into the soma, and conversely somatodendritic surface proteins, which enter the axon, are efficiently endocytosed to preserve the neuronal architecture. A mechanism is readily conceivable, by which MAD2 mediates endocytosis of somatic SERT in order to support its rerouting into the axon.”

4. Images do not backup the interaction of MAD2 and SERT with AP2. I would like to see an image revealing the co-localization of these three proteins in a neuronal compartment.

Reply:
As mentioned above (see reply to the general comments inserted directly above “Major points”), the dwell time of SERT on the surface of the neuronal soma is short. In fact, under basal conditions (i.e. without MAD2 knockdown), we find only a very minute fraction of SERT on the surface (cf. Fig. EV4 & EV5). Hence, we consider it highly unlikely that native complexes between SERT, MAD2 and AP2 can be visualized in neurons.

To address the referee’s point, we conducted additional experiments in JAR cells, a cell line of placental origin, which endogenously expresses SERT and where evidence for SERT internalization has been obtained (see e.g. doi: 10.1074/jbc.M113.495754). We visualized the co-localization of mCherry-SERT, YFP-MAD2 and β2-adaptin in JAR cells; a pertinent figure is inserted below for the referee’s perusal (“Referee response Fig. 3”): several puncta were detected in which mCherry-SERT, YFP-MAD2 and β2-adaptin co-localized.

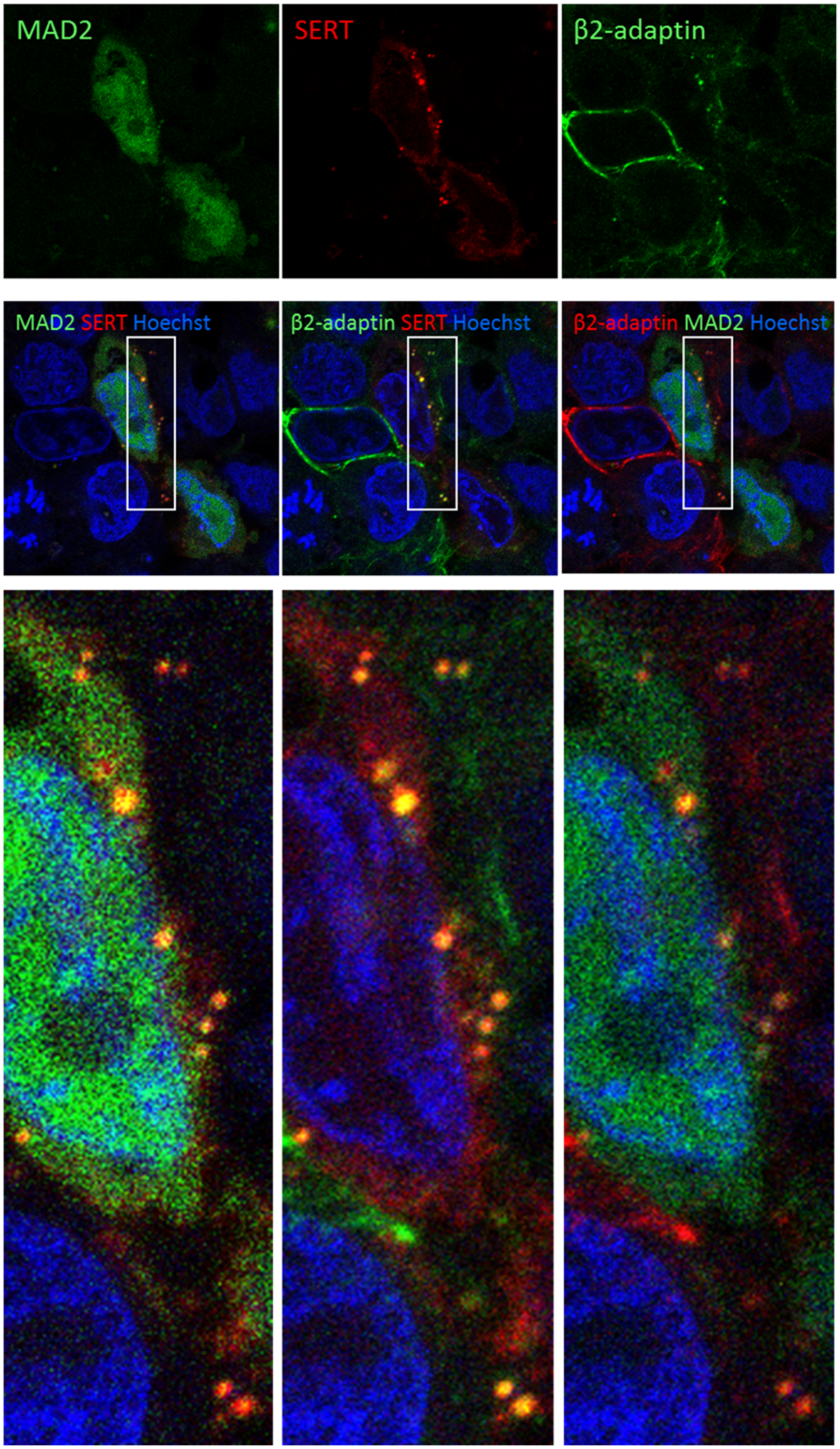

Referee response Fig. 3: JAR-cells were co-transfected with mCherry-SERT and YFP-MAD2 and (on the following day) incubated for 10 min in a solution containing 10 µM 5-HT and 40 µM of the endocytosis inhibitor dynasore. Subsequently, cells were fixed in 4% PFA and AP2 was labelled with mouse-anti-β2-adaptin, which was subsequently detected with anti-mouse AF647. Images were captured by confocal laser scanning microscopy (at 60-fold magnification). The rectangle delineates the area, which was magnified to visualize punctate co-localization.

This methodological approach is, in principle, applicable to primary neuronal cultures, but it is of limited value for the following reasons: (i) cultures prepared from dorsal raphe nuclei are invariably a mixture of many types of neurons (and of glial cells). Transfection of exogenous SERT would necessarily result in its expression in all cell types of the culture and not only serotonergic neurons, which would render the latter unidentifiable. (ii) Even if this obstacle can be overcome, exogenous SERT (tagged by a fluorescent protein) must behave like endogenous SERT for the experiment to be meaningful. Hence, in serotonergic neurons, exogenous fluorescently SERT also ought to reside intracellularly in the soma, which rules out co-localization with AP2 at the plasma membrane.
We agree that visual detection of SERT/MAD2/AP2 complexes in neurons would be confirmatory for our findings. However, we emphasize again that the inability to detect these complexes due to their very transient nature does not diminish the findings of our study.

5. Trafficking of SERT. Fig.5E should be carried out using live imaging. For example, looking at the recycling of SERT-phluorin with endogenous and decreased levels of MAD2. This could be an important experiment to demonstrate that the proposed interaction is relevant to the internalization of SERT.

Reply:
If we understand this point correctly, referee #3 recommends that a transgenic animal be generated with the SERT-locus replaced by SERT-pHluorin, presumably (super)ratiometric pHluorin-2 rather than (super)ecliplitic pHluorin. Then, cultures of serotonergic neurons should be obtained from these animals. Endocytosis of this tagged SERT should then be investigated with a protein-tag (pHluorin) which changes the ratiometric fluorescence signal upon internalization due to the decreased pH.
With all due respect, we think that this set of experiments is beyond the scope of the current study. We also stress that we do not consider transfection/transduction of SERT-pHluorin into native neuronal cultures an appropriate approach: cultures prepared from dorsal raphe nuclei are invariably a mixture of many types of neurons. Thus, a simple trans-/infection experiment is unlikely to provide reliable data, because a mixture of heterogeneous neurons will express SERT-pHluorin and there is no way to tell in live-cell imaging, which neuron is natively serotonergic (see also reply to point 4).
In addition, we would like to point out that both: the C- and the N-terminus of SERT (and all related transporters) are cytosolic and hence inaccessible to the pH-changes between the extracellular and the endocytic compartment. The pHluorin-tag would thus need to be attached to an artificial 13th transmembrane domain or within an extracellular protein-region - presumably extracellular loop-2 (EL2) or EL4, because the other loops are too short to tolerate an insertion. However, the N-terminus of SERT (and of DAT) is required for the switching from the forward cycling mode into the exchange mode (i.e. the basis for amphetamine-induced transmitter release - see doi: 10.1074/jbc.M116.771360 & doi: 10.1074/jbc.M109.083154) and the (free) C-terminus is required for proper transporter folding (doi: 10.1074/jbc.M115.641357). Similarly, the movement of EL2 and EL4 play important roles in the transport cycle of SERT (doi: 10.1016/j.jbc.2021.100863). Hence, these constructs are very likely to suffer from deficiencies, which make them unlikely to reflect the characteristics of wild type SERT.

Minor comments:
1. Presence of MAD2 in glial cells. Lines 317-322 identifies MAD2 is in glial cells, however, line 387 makes an opposite statement based on the literature "MAD2 was present in neurons but not in glial cells". Please clarify.

Reply:
We thank referee #3 for pointing out the discrepancy between our observations (on rat glial cultures) and the human protein atlas. This sentence was deleted (as were many other sentences) to meet the space requirements. We think that it is of modest interest to address the discrepancy (in glial

expression of MAD2) between our findings and the data in the human protein atlas (which are presumably more coarse-grain).

2. Figure legends of images: uM to um.

Reply: we apologize for the mistake and thank the referee for pointing it out. All legends now refer to µm.

3. Fig. EV2. Line fits should begin at 0,0. The offset in the y axes cannot be interpreted.

Reply:
(i) If we force the lines through 0, the slopes of the correlation lines in panels B, C and D of EV2 become steeper (and the correlation is enhanced). We do not consider this a legitimate approach.
(ii) We agree with the second statement: we do not have any firm evidence, which allows for interpreting the offset (y-intercept): it may reflect (i) the noise in the experiments (presumably most relevant in panels B, C and D), (ii) the portion of binding, which is independent of MAD2 (panel E). However, we consider this issue of modest interest and thus should prefer not to have to discuss it.

1st Revision - Editorial Decision 12th Jun 2023

Dear Florian,

Thank you for the submission of your revised manuscript to EMBO reports. We have now received the full set of referee reports that is copied below.

As you will see, all referees are very positive about the study and request only minor changes to clarify text and figures and to improve data and image presentation.

From the editorial side, there are also a few things that we need before we can proceed with the official acceptance of your study.

- You use rats and mice to establish primary neuronal cultures, tissue sections and brain extracts. Please fill in the relevant sections in our Author Checklist regarding the use of experimental animals and authority granting approval and list this information in the Methods section.

- Please add a 'Disclosure and competing interests statement'. For more information see https://www.embopress.org/page/journal/14693178/authorguide#conflictsofinterest

- Please ensure that you have entered and completed all information on author contributions in our online submission system. This information will be automatically retrieved and typeset into your article.

- We recommend arranging the figure panels in a manner that they can be called out in an alphabetical order. Currently you callout Fig. 1B after Fig. 1C. I think these panels could easily swapped. If you swap these, please relabel the source data for Fig. 1 accordingly.

- Please add callouts to the following figure panels in the text: Fig. 3H, 5G, EV1C, EV4A.

- Please supply the synopsis image in either PNG or JPG format. The final dimensions are: 550x300-600 pixels (width x height). Please ensure that the text is eligible at this final size. It might be better - given the size limitations - to remove the brain schematic and focus on the right part of the illustration.

- Please also supply a draft for a short summary (1-2 sentences) and 2-3 bullet points highlighting key results.

- I attach to this email a related manuscript file with comments by our data editors (mainly in the figure legends). Please address all comments and upload a revised file with tracked changes with your final manuscript submission.

We look forward to seeing a final version of your manuscript as soon as possible.

With kind regards,

Martina

Martina Rembold, PhD
Senior Editor
EMBO reports

****

Referee #1:

The authors have done an admirable job addressing all of the reviewers concerns with additional data and changes to the text. Further experiments beyond those included in the revision would be beyond the scope of this brief but important report.

Referee #2:

Koban and Freissmuth addressed our concerns convincingly. The authors modified the manuscript which improves it substantially in our regards. We only would like to suggest the following minor point.

page 11, lines 309:
The statement that Rab7a/Rab11a are "virtually undetectable" in neurite extensions seems not correct (see for example, Fig. EV3A and neuron on the right side in Fig. EV3B). We would suggest that the authors phrase this conclusion more cautiously.

Referee #3:

The authors have improved the manuscript and now conveys a clearer message. The previous title "The mitotic checkpoint protein MAD2 delivers the serotonin transporter to endocytosis" has been substituted by "The cell cycle protein MAD2 facilitates endocytosis of the serotonin transporter in the neuronal soma". The biochemical findings are complemented with cell biology experiments in HEK cells and cultured dorsal raphe neurons. I am convinced by biochemical and cell biology data obtained in HEK cells, however, I still find that images should be improved to better illustrate the results of quantifications. I also would like to see in more detail data from cultured neurons. The authors can easily address these issues by improving data presentation.

Major comment:
Figure 5 shows images of two neurons (panel E) to illustrate results. Images on the left (20x objective) have a different appearance from images on the right (60x objective). According to the legend, we are looking at single sections. The 20x image for MAD-sh shows a cytoplasmic staining of SERT in the soma that cannot be appreciated in the 60x image. The reason could be a larger optical section for the 20x objective. If so, the legend should provide details. I cannot see the method used to quantify surface/intracellular SERT in confocal images of cultured neurons. Was it done using line profiles or ROIs? This is a key aspect to sustain the claims of the paper. The authors should provide more visual examples of SERT distribution in the soma in the experimental conditions used since this observation is directly related to the title of the paper. The image of one neuron/condition is not enough.

Minor comments:
Figure 4 images (C, E and G). Cells should be shown at a higher magnification, for example using insets, to illustrate the reduced presence of YFP-SERT in the cytoplasm after treatment with MAD2 siRNA.
Figure 5E. Does the scale bar in central images indicate arbitrary units of fluorescence? If so, please explain in legend.
An intriguing question that is not discussed in the paper is how the proposed mechanism relates to the recycling of SERT in non-somatic locations.

Thank you for the submission of your revised manuscript to EMBO reports. We have now received the full set of referee reports that is copied below.

As you will see, all referees are very positive about the study and request only minor changes to clarify text and figures and to improve data and image presentation.

From the editorial side, there are also a few things that we need before we can proceed with the official acceptance of your study.

- You use rats and mice to establish primary neuronal cultures, tissue sections and brain extracts. Please fill in the relevant sections in our Author Checklist regarding the use of experimental animals and authority granting approval and list this information in the Methods section.

- Please add a 'Disclosure and competing interests statement'. For more information see https://www.embopress.org/page/journal/14693178/authorguide#conflictsofinterest

- Please ensure that you have entered and completed all information on author contributions in our online submission system. This information will be automatically retrieved and typeset into your article.

- We recommend arranging the figure panels in a manner that they can be called out in an alphabetical order. Currently you callout Fig. 1B after Fig. 1C. I think these panels could easily swapped. If you swap these, please relabel the source data for Fig. 1 accordingly.

- Please add callouts to the following figure panels in the text: Fig. 3H, 5G, EV1C, EV4A.

- Please supply the synopsis image in either PNG or JPG format. The final dimensions are: 550x300-600 pixels (width x height). Please ensure that the text is eligible at this final size. It might be better - given the size limitations - to remove the brain schematic and focus on the right part of the illustration.

- Please also supply a draft for a short summary (1-2 sentences) and 2-3 bullet points highlighting key results.

- I attach to this email a related manuscript file with comments by our data editors (mainly in the figure legends). Please address all comments and upload a revised file with tracked changes with your final manuscript submission.

We look forward to seeing a final version of your manuscript as soon as possible.

With kind regards,

Martina

Martina Rembold, PhD
Senior Editor
EMBO reports

****

Referee #1:

The authors have done an admirable job addressing all of the reviewers concerns with additional data and changes to the text. Further experiments beyond those included in the revision would be beyond the scope of this brief but important report.

Referee #2:

Koban and Freissmuth addressed our concerns convincingly. The authors modified the manuscript which improves it substantially in our regards. We only would like to suggest the following minor point.

page 11, lines 309:
The statement that Rab7a/Rab11a are "virtually undetectable" in neurite extensions seems not correct (see for example, Fig. EV3A and neuron on the right side in Fig. EV3B). We would suggest that the authors phrase this conclusion more cautiously.

Referee #3:

The authors have improved the manuscript and now conveys a clearer message. The previous title "The mitotic checkpoint protein MAD2 delivers the serotonin transporter to endocytosis" has been substituted by "The cell cycle protein MAD2 facilitates endocytosis of the serotonin transporter in the neuronal soma". The biochemical findings are complemented with cell biology experiments in HEK cells and cultured dorsal raphe neurons. I am convinced by biochemical and cell biology data obtained in HEK cells, however, I still find that images should be improved to better illustrate the results of quantifications. I also would like to see in more detail data from cultured neurons. The authors can easily address these issues by improving data presentation.

Major comment:
Figure 5 shows images of two neurons (panel E) to illustrate results. Images on the left (20x objective) have a different appearance from images on the right (60x objective). According to the legend, we are looking at single sections. The 20x image for MAD-sh shows a cytoplasmic staining of SERT in the soma that cannot be appreciated in the 60x image. The reason could be a larger optical section for the 20x objective. If so, the legend should provide details. I cannot see the method used to quantify surface/intracellular SERT in confocal images of cultured neurons. Was it done using line profiles or ROIs? This is a key aspect to sustain the claims of the paper. The authors should provide more visual examples of SERT distribution in the soma in the experimental conditions used since this observation is directly related to the title of the paper. The image of one neuron/condition is not enough.

Minor comments:
Figure 4 images (C, E and G). Cells should be shown at a higher magnification, for example using insets, to illustrate the reduced presence of YFP-SERT in the cytoplasm after treatment with MAD2 siRNA.
Figure 5E. Does the scale bar in central images indicate arbitrary units of fluorescence? If so, please explain in legend.
An intriguing question that is not discussed in the paper is how the proposed mechanism relates to the recycling of SERT in non-somatic locations.

Comment to referee #3:
Referee #3 comments on the fact that in “Fig. 5E MAD2-sh 20x” SERT appears in the cytosol, which cannot be appreciated in the 60x image. The referee attributes this to a larger optical section for the 20x objective. This is the case. As the pinhole width was left constant over all magnifications optical sections grow thicker (by roughly 3x) as the numerical aperture (NA) and the refractive index (n) decrease (60x: NA = 1.4, n = 1.5(oil) vs. 20x: NA = 0.75, n = 1 (air)).
Furthermore, in the pertinent figure, SERT looks cytosolic mainly because of the high color

saturation. It should be noted, that the 20x magnification in Fig 5 is of rather illustrative character, in order to show that the 60x images derive from intact widely ramified serotonergic neurons. The original data files of these images (with the original color saturation) also convincingly convey the impression that SERT concentrates at the surface upon MAD2-depletion and that the alleged cytosolic SERT-signal rather seems to derive from neuronal extensions which happen to wrap over the cell body.
Overall, we do not think that these details are of much interest to the readership of EMBO reports. Also, because of space constraints, we should rather prefer not to discuss this aspect.

Instead, the current figure legend contains the sentence: “Due to imaging conditions the optical section thickness is increased at 20× compared to 60× magnification.”

Text changes:

1. „Author contributions“ were removed and the according boxes checked in the online submission system.
2. „Competing interest statement“ was included below the acknowledgments.
3. Keywords: Serotonin transporter/ raphe neurons/ mitotic checkpoint/ endocytosis/ recycling endosome.
4. The paragraphs describing Fig. 1B and Fig. 1C were swapped in the main text, so that Fig. 1B is now described before Fig. 1C.
5. Fig. EV1C, 3H, EV4A and 5G are now called out in the main text.
6. The statement that Rab7 and Rab11 are “virtually undetectable in serotonergic neurite extensions” was softened to “virtually undetectable in distant serotonergic neurite extensions”, to account for the fact that both proteins can be found in extensions of serotonergic neurons which are rather close to the soma. However, the distant greater network of serotonergic extensions is, in fact, completely devoid of Rab7 and Rab11 (please see comment by referee #2).
7. The “Materials and Methods” section now contains an “Animals”-paragraph, containing information on animal welfare and handling. The author checklist was updated accordingly.
8. The legend for fig 5C now specifies ****p < 0.0001 in an unpaired two-tailed t-test.
9. The legend for fig. 5E now specifies that “Calibration bars in images showing rMAD2 represent arbitrary fluorescence units” (please see comment by referee #3).
10. The legend for fig. 5F now specifies that “regions of interests (ROIs)” were used for quantification (please see comment by referee #3).
11. Scale bars were added to the figures according to the editorial comments. The figure legends were changed accordingly. Error bars for the uptake saturation curve in Fig. 4A were specified as SEM.
12. For figure legend EV2A the number of biological replicates was provided. But note: as figures B – E are the correlative representation of fig. A, the number of replicates is identical (exemption: as MAD2 is the correlation partner for all the other proteins, n for MAD2 does not appear separately in figure legends for B – E but only in the figure legend for A).

Figure changes:

13. Fig. 1: Scale bars were added as required and their thickness increased for better visibility.
14. Fig. 3: Scale bars were added as required and their thickness increased for better visibility. Fig. 3C was re-labeled to read: TCL; untransf.; YFP-hDAT and YFP-hSERT instead of: TCL (HEK-293); empty 293; YFP-DAT and YFP-SERT.
    In Fig. 3E a "significance star" was added to correctly indicate p < 0.0001.
15. Fig. 4: Inserts of zoomed areas were added including scale bars. Figure legends were changed accordingly (please see comment by referee #3). Fig. 4B was re-labeled.
16. Fig. 5: In Fig. 5F additional representative images were inserted showing serotonergic neurons expressing either scramble-shRNA or MAD2-shRNA. (Please see comment by referee #3). The figure legend was changed accordingly.
    In Fig. 5G the "brain part" was removed and the scheme of the serotonin transporter was revised in order to better reflect the molecular architecture of the protein.
17. Fig. EV1: Scale bars were added as required.
18. Fig. EV4 and EV5: Scale bars were added as required.

Changes to original data files:

19. In the Readme files for Fig. 1B, 3A and 3B a typo was corrected.
20. Original files for the 8 representative images in Fig. 5F were added.
21. Some of the original image files for fig. 5E and F could only be opened with ImageJ software but were completely black when opened with preinstalled software (i.e., by double-clicking on the image) – the reason is unknown.
    The problem was solved by converting the respective images to JPEG followed by re-conversion to TIFF. The original files were updated accordingly.
22. Subfolders called _MACOSX still appear in the folders for Fig. 1, 3 and 5, obviously because I updated these folders on my MacBook. I should be happy and grateful if the EMBO reports-team could just delete these folders.

Dr. Florian Koban
Medical University of Vienna
Institute of Pharmacology
Medical University of Vienna
Währinger Str. 13a
Vienna, A-1090 Vienna A-1090
Austria

Dear Florian,

I am very pleased to accept your manuscript for publication in the next available issue of EMBO reports. Thank you for your contribution to our journal.

At the end of this email I include important information about how to proceed. Please ensure that you take the time to read the information and complete and return the necessary forms to allow us to publish your manuscript as quickly as possible.

As part of the EMBO publication's Transparent Editorial Process, EMBO reports publishes online a Review Process File to accompany accepted manuscripts. As you are aware, this File will be published in conjunction with your paper and will include the referee reports, your point-by-point response and all pertinent correspondence relating to the manuscript.

If you do NOT want this File to be published, please inform the editorial office within 2 days, if you have not done so already, otherwise the File will be published by default [contact: emboreports@embo.org]. If you do opt out, the Review Process File link will point to the following statement: "No Review Process File is available with this article, as the authors have chosen not to make the review process public in this case."

Should you be planning a Press Release on your article, please get in contact with emboreports@wiley.com as early as possible, in order to coordinate publication and release dates.

On a different note, I would like to alert you that EMBO Press offers a new format for a video-synopsis of work published with us, which essentially is a short, author-generated film explaining the core findings in hand drawings, and, as we believe, can be very useful to increase visibility of the work. This has proven to offer a nice opportunity for exposure i.p. for the first author(s) of the study. Please see the following link for representative examples and their integration into the article web page:
https://www.embopress.org/video_synopses
https://www.embopress.org/doi/full/10.15252/embj.2019103932
Please let me know, should you be interested to engage in commissioning a similar video synopsis for your work. According operation instructions are available and intuitive.

Thank you again for your contribution to EMBO reports and congratulations on a successful publication. Please consider us again in the future for your most exciting work.

Kind regards,

Martina

Martina Rembold, PhD
Senior Editor
EMBO reports

****

THINGS TO DO NOW:

Please note that you will be contacted by Wiley Author Services to complete licensing and payment information. The required 'Page Charges Authorization Form' is available here: https://www.embopress.org/pb-assets/embo-site/er_apc.pdf - please download and complete the form and return to embopressproduction@wiley.com

EMBO Press participates in many Publish and Read agreements that allow authors to publish Open Access with reduced/no publication charges. Check your eligibility: https://authorservices.wiley.com/author-resources/Journal-Authors/open-access/affiliation-policies-payments/index.html

You will receive proofs by e-mail approximately 2-3 weeks after all relevant files have been sent to our Production Office; you should return your corrections within 2 days of receiving the proofs.

Please inform us if there is likely to be any difficulty in reaching you at the above address at that time. Failure to meet our deadlines may result in a delay of publication, or publication without your corrections.

All further communications concerning your paper should quote reference number EMBOR-2021-53408V4 and be addressed to emboreports@wiley.com.

Should you be planning a Press Release on your article, please get in contact with emboreports@wiley.com as early as possible, in order to coordinate publication and release dates.

EMBO Press Author Checklist

Corresponding Author Name: Florian Koban	
Journal Submitted to: EMBO Reports	
Manuscript Number: EMBOR-2021-53408V2-Q	

USEFUL LINKS FOR COMPLETING THIS FORM
The EMBO Journal - Author Guidelines
EMBO Reports - Author Guidelines
Molecular Systems Biology - Author Guidelines
EMBO Molecular Medicine - Author Guidelines

Reporting Checklist for Life Science Articles (updated January 2022)
This checklist is adapted from Materials Design Analysis Reporting (MDAR) Checklist for Authors. MDAR establishes a minimum set of requirements in transparent reporting in the life sciences (see Statement of Task: 10.31222/osf.io/9sm4x). Please follow the journal's guidelines in preparing your manuscript.
Please note that a copy of this checklist will be published alongside your article.

Abridged guidelines for figures
1. Data
The data shown in figures should satisfy the following conditions:
- the data were obtained and processed according to the field's best practice and are presented to reflect the results of the experiments in an accurate and unbiased manner.
- ideally, figure panels should include only measurements that are directly comparable to each other and obtained with the same assay.
- plots include clearly labeled error bars for independent experiments and sample sizes. Unless justified, error bars should not be shown for technical replicates.
- if n<5, the individual data points from each experiment should be plotted. Any statistical test employed should be justified.
- Source Data should be included to report the data underlying figures according to the guidelines set out in the authorship guidelines on Data Presentation.

2. Captions
Each figure caption should contain the following information, for each panel where they are relevant:
- a specification of the experimental system investigated (eg cell line, species name).
- the assay(s) and method(s) used to carry out the reported observations and measurements.
- an explicit mention of the biological and chemical entity(ies) that are being measured.
- an explicit mention of the biological and chemical entity(ies) that are altered/varied/perturbed in a controlled manner.
- the exact sample size (n) for each experimental group/condition, given as a number, not a range;
- a description of the sample collection allowing the reader to understand whether the samples represent technical or biological replicates (including how many animals, litters, cultures, etc.).
- a statement of how many times the experiment shown was independently replicated in the laboratory.
- definitions of statistical methods and measures:
  - common tests, such as t-test (please specify whether paired vs. unpaired), simple χ2 tests, Wilcoxon and Mann-Whitney tests, can be unambiguously identified by name only, but more complex techniques should be described in the methods section;
  - are tests one-sided or two-sided?
  - are there adjustments for multiple comparisons?
  - exact statistical test results, e.g., P values = x but not P values < x;
  - definition of 'center values' as median or average;
  - definition of error bars as s.d. or s.e.m.

Please complete ALL of the questions below.
Select "Not Applicable" only when the requested information is not relevant for your study.

Materials

Newly Created Materials	Information included in the manuscript?	In which section is the information available? (Reagents and Tools Table, Materials and Methods, Figures, Data Availability Section)
New materials and reagents need to be available; do any restrictions apply?	Not Applicable	

Antibodies	Information included in the manuscript?	In which section is the information available? (Reagents and Tools Table, Materials and Methods, Figures, Data Availability Section)
For **antibodies** provide the following information:<br>- Commercial antibodies: RRID (if possible) or supplier name, catalogue number and or/clone number<br>- Non-commercial: RRID or citation	Yes	Materials and Methods

DNA and RNA sequences	Information included in the manuscript?	In which section is the information available? (Reagents and Tools Table, Materials and Methods, Figures, Data Availability Section)
**Short novel DNA or RNA including primers, probes**: provide the sequences.	Yes	Materials and Methods

Cell materials	Information included in the manuscript?	In which section is the information available? (Reagents and Tools Table, Materials and Methods, Figures, Data Availability Section)
**Cell lines:** Provide species information, strain. Provide accession number in repository **OR** supplier name, catalog number, clone number, and/**OR** RRID.	Yes	Main text/Materials and Methods/Figures
**Primary cultures:** Provide species, strain, sex of origin, genetic modification status.	Yes	Main text/Materials and Methods/Figures
Report if the cell lines were recently **authenticated** (e.g., by STR profiling) and tested for mycoplasma contamination.	Yes	Cell lines were free of mycoplasma contamination

Experimental animals	Information included in the manuscript?	In which section is the information available? (Reagents and Tools Table, Materials and Methods, Figures, Data Availability Section)
**Laboratory animals or Model organisms:** Provide species, strain, sex, age, genetic modification status. Provide accession number in repository **OR** supplier name, catalog number, clone number, **OR** RRID.	Yes	Materials and Methods
**Animal observed in or captured from the field:** Provide species, sex, and age where possible.	Not Applicable	
Please detail **housing and husbandry conditions**.	Yes	Materials and Methods

Plants and microbes	Information included in the manuscript?	In which section is the information available? (Reagents and Tools Table, Materials and Methods, Figures, Data Availability Section)
**Plants:** provide species and strain, ecotype and cultivar where relevant, unique accession number if available, and source (including location for collected wild specimens).	Not Applicable	
**Microbes:** provide species and strain, unique accession number if available, and source.	Not Applicable	

Human research participants	Information included in the manuscript?	In which section is the information available? (Reagents and Tools Table, Materials and Methods, Figures, Data Availability Section)
If collected and within the bounds of privacy constraints report on age, sex and gender or ethnicity for all study participants.	Not Applicable	

Core facilities	Information included in the manuscript?	In which section is the information available? (Reagents and Tools Table, Materials and Methods, Figures, Data Availability Section)
If your work benefited from core facilities, was their service mentioned in the acknowledgments section?	Not Applicable	

Design

Study protocol	Information included in the manuscript?	In which section is the information available? (Reagents and Tools Table, Materials and Methods, Figures, Data Availability Section)
If study protocol has been **pre-registered, provide DOI in the manuscript**. For clinical trials, provide the trial registration number **OR** cite DOI.	Not Applicable	
Report the **clinical trial registration number** (at ClinicalTrials.gov or equivalent), where applicable.	Not Applicable	

Laboratory protocol	Information included in the manuscript?	In which section is the information available? (Reagents and Tools Table, Materials and Methods, Figures, Data Availability Section)
Provide DOI OR other citation details if **external detailed step-by-step protocols** are available.	Not Applicable	

Experimental study design and statistics	Information included in the manuscript?	In which section is the information available? (Reagents and Tools Table, Materials and Methods, Figures, Data Availability Section)
Include a statement about **sample size** estimate even if no statistical methods were used.	Not Applicable	
Were any steps taken to minimize the effects of subjective bias when allocating animals/samples to treatment (e.g. **randomization procedure**)? If yes, have they been described?	Not Applicable	
Include a statement about **blinding** even if no blinding was done.	Not Applicable	
Describe **inclusion/exclusion criteria** if samples or animals were excluded from the analysis. Were the criteria pre-established?<br><br>If sample or data points were omitted from analysis, report if this was due to attrition or intentional exclusion and provide justification.	Not Applicable	
For every figure, are **statistical tests** justified as appropriate? Do the data meet the assumptions of the tests (e.g., normal distribution)? Describe any methods used to assess it. Is there an estimate of variation within each group of data? Is the variance similar between the groups that are being statistically compared?	Yes	Figures/Material and Methods

Sample definition and in-laboratory replication	Information included in the manuscript?	In which section is the information available? (Reagents and Tools Table, Materials and Methods, Figures, Data Availability Section)
In the figure legends: state number of times the experiment was **replicated** in laboratory.	Yes	Figure legends
In the figure legends: define whether data describe **technical or biological replicates**.	Yes	Figure legends/Materials and Methods

Ethics

Ethics	Information included in the manuscript?	In which section is the information available? (Reagents and Tools Table, Materials and Methods, Figures, Data Availability Section)
Studies involving **human participants**: State details of **authority granting ethics approval** (IRB or equivalent committee(s), provide reference number for approval.	Not Applicable	
Studies involving **human participants**: Include a statement confirming that **informed consent** was obtained from all subjects and that the experiments conformed to the principles set out in the WMA Declaration of Helsinki and the Department of Health and Human Services Belmont Report.	Not Applicable	
Studies involving **human participants:** For publication of **patient photos**, include a statement confirming that consent to publish was obtained.	Not Applicable	
Studies involving experimental **animals**: State details of **authority granting ethics approva**l (IRB or equivalent committee(s), provide reference number for approval. Include a statement of compliance with ethical regulations.	Not Applicable	Materials and Methods
Studies involving **specimen and field samples:** State if relevant **permits** obtained, provide details of authority approving study; if none were required, explain why.	Not Applicable	

Dual Use Research of Concern (DURC)	Information included in the manuscript?	In which section is the information available? (Reagents and Tools Table, Materials and Methods, Figures, Data Availability Section)
Could your study fall under dual use research restrictions? Please check biosecurity documents and list of **select agents and toxins** (CDC): https://www.selectagents.gov/sat/list.htm	Not Applicable	
If you used a select agent, is the security level of the lab appropriate and reported in the manuscript?	Not Applicable	
If a study is subject to dual use research of concern regulations, is the name of the **authority granting approval and reference number** for the regulatory approval provided in the manuscript?	Not Applicable	

Reporting

The MDAR framework recommends adoption of discipline-specific guidelines, established and endorsed through community initiatives. Journals have their own policy about requiring specific guidelines and recommendations to complement MDAR.

Adherence to community standards	Information included in the manuscript?	In which section is the information available? (Reagents and Tools Table, Materials and Methods, Figures, Data Availability Section)
State if relevant guidelines or checklists (e.g., **ICMJE, MIBBI, ARRIVE, PRISMA**) have been followed or provided.	Not Applicable	
For **tumor marker prognostic studies,** we recommend that you follow the **REMARK** reporting guidelines (see link list at top right). See author guidelines, under 'Reporting Guidelines'. Please confirm you have followed these guidelines.	Not Applicable	
For **phase II and III randomized controlled trials**, please refer to the **CONSORT** flow diagram (see link list at top right) and submit the CONSORT checklist (see link list at top right) with your submission. See author guidelines, under 'Reporting Guidelines'. Please confirm you have submitted this list.	Not Applicable	

Data Availability

Data availability	Information included in the manuscript?	In which section is the information available? (Reagents and Tools Table, Materials and Methods, Figures, Data Availability Section)
Have **primary datasets** been deposited according to the journal's guidelines (see 'Data Deposition' section) and the respective accession numbers provided in the Data Availability Section?	Not Applicable	
Were **human clinical and genomic datasets** deposited in a public access-controlled repository in accordance to ethical obligations to the patients and to the applicable consent agreement?	Not Applicable	
Are **computational models** that are central and integral to a study available without restrictions in a machine-readable form? Were the relevant accession numbers or links provided?	Not Applicable	
If publicly available data were reused, provide the respective **data citations in the reference list**.	Not Applicable	
